# Mechanistic Divergence in Sulfur‐Ligated Iron(III)‐Alkylperoxo Reactivity: Aldehyde Oxidation Prevails over Deformylation

**DOI:** 10.1002/anie.202512839

**Published:** 2025-09-04

**Authors:** Jagnyesh K. Satpathy, Rolly Yadav, Payal Panwar, Vijaya Thangaraj, Maheswaran Shanmugam, Chivukula V. Sastri, Sam P. de Visser

**Affiliations:** ^1^ Department of Chemistry Indian Institute of Technology Guwahati Assam 781039 India; ^2^ Department of Chemistry Indian Institute of Technology Bombay, Powai Mumbai Maharashtra 400076 India; ^3^ The Manchester Institute of Biotechnology and Department of Chemical Engineering The University of Manchester 131 Princess Street Manchester M1 7DN UK

**Keywords:** Biomimetic models, DFT calculations, Inorganic reaction mechanisms, Kinetics, Sulfoxidation

## Abstract

Metalloenzymes activate molecular oxygen within their catalytic cycles to generate a reactive species capable of substrate transformation. In many iron‐containing enzymes, it is a high‐valent iron(IV)‐oxo complex that is synthesized from an iron(III)‐alkylperoxo intermediate, although direct observation and characterization of such species have remained elusive, leaving their mechanistic role uncertain. To address this gap in our understanding, we present here the synthesis, comprehensive characterization, and reactivity of a novel thioether‐ligated iron(III)‐alkylperoxo complex supported by the ligand 2‐((2‐(pyridin‐2‐yl)ethyl)thio)‐*N*,*N*‐bis(pyridin‐2‐ylmethyl)ethan‐1‐amine. Characterization was done using UV–vis spectroscopy, resonance Raman spectroscopy, electron paramagnetic resonance spectroscopy, and electrospray ionization mass spectrometry. Reactivity studies reveal that this complex exhibits electrophilic oxidation of model substrates, including dimethylsulfide, triphenylphosphine, and cyclohexanecarboxaldehyde. Notably, the latter substrate reacts via the unusual aldehyde C─H bond abstraction leading to cyclohexanecarboxylic acid, which is explained by favorable aldehyde C─H abstraction transition states due to stabilizing interactions between the ligand framework and the substrate. Moreover, the reaction is initiated with a homolytic O─O bond cleavage in the iron(III)‐alkylperoxo group that yields a reactive iron(IV)‐oxo species that mediates substrate oxidation. To our knowledge, this work represents the first example of a mononuclear low‐spin (*S* = ½) nonheme iron(III)‐alkylperoxo complex displaying such unprecedented electrophilic reactivity.

## Graphical abstract

This study explores the mechanistic investigation into an unusual reactivity of a thioether‐ligated low‐spin Fe(III)‐alkylperoxide with cyclohexane–carboxaldehyde as a mimic of nonheme iron(II) enzyme intermediates and highlights that only efficient catalysis happens from a high‐valent iron(IV)‐oxo species.

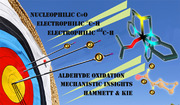

## Introduction

The activation and use of dioxygen and its associated compounds, like hydrogen peroxide and superoxide, hold significant importance for many mononuclear heme and nonheme iron‐containing enzymes.^[^ ^1^, ^2^, ^3^, ^4^, ^5^, ^6^, ^7^, ^8^, ^9^, ^10^, ^11^, ^12^, ^13^, ^14^, ^15^, ^16^, ^17^, ^18^, ^19^, ^20^, ^21^, ^22^, ^23^ ^]^ In the catalytic cycles of these iron‐containing enzymes typically an iron–oxygen adduct is generated after dioxygen binding in the form of an iron(III)‐superoxo species that then acts as a precursor for the biosynthesis of the active oxidant, which often is an iron(IV)‐oxo species. The latter then performs a catalytic reaction mechanism with the available substrate in the substrate‐binding pocket leading to oxygenation or desaturation of the substrate. This process in biology is a means to trigger the biodegradation of toxic compounds or the biosynthesis of natural products such as hormones.^[^ ^24^, ^25^, ^26^, ^27^ ^]^ For instance, the catalytically active species in the cytochrome P450s is Compound I or an iron(IV)‐oxo with a heme cation radical and it is formed from the resting state of the enzyme after water release from the heme, the reduction of the five‐coordinate iron(III)‐heme and followed by dioxygen binding, a further heme reduction and two proton transfer steps.^[^ ^1^, ^2^, ^3^, ^4^, ^5^, ^6^, ^7^, ^8^, ^9^, ^10^, ^11^, ^12^, ^13^ ^]^ By contrast, in the nonheme iron dioxygenases no redox partners and proton sources are used but instead a co‐substrate, such as α‐ketoglutarate, is utilized.^[^ ^14^, ^15^, ^16^, ^17^, ^18^, ^19^, ^20^, ^21^, ^22^, ^23^, ^24^, ^25^, ^26^, ^27^ ^]^ Nonheme iron dioxygenases play pivotal roles in the human body and take part in the biosynthesis of hydroxyproline in the skin,^[^ ^28^, ^29^, ^30^ ^]^ as well as the catabolism of toxic cysteine in the brain.^[^ ^31^, ^32^, ^33^, ^34^ ^]^ In the catalytic cycle of α‐ketoglutarate‐dependent nonheme iron dioxygenases, dioxygen binds on an iron(II) center and attacks the equatorially bound α‐ketoglutarate co‐substrate to produce an iron(III)‐alkylperoxo complex. The latter is believed to form an iron(IV)‐oxo species through homolytic O─O bond cleavage and release of CO_2_.^[^ ^14^, ^15^, ^16^, ^17^, ^18^, ^19^, ^20^, ^21^, ^22^, ^23^, ^24^, ^25^, ^26^, ^27^ ^]^ The iron(III)‐persuccinate complex is a fleeting intermediate in the catalytic cycle of the α‐ketoglutarate‐dependent nonheme iron dioxygenases and has never been characterized experimentally, although there may be spectroscopic evidence of an iron(III)‐superoxo intermediate in the catalytic cycle of cysteine dioxygenase; however, the data was inconclusive.^[^ ^34^ ^]^ Nevertheless, an iron(III)‐alkylperoxo intermediate has been trapped with crystallography for the enzyme homoprotocatechuate‐2,3‐dioxygenase, which, as far as we know, is the only enzymatic example of an alkylperoxo intermediate found in an enzyme.^[^ ^35^ ^]^

Another example of an oxygen‐activating metalloenzyme is superoxide reductase, which is present in anaerobic and microaerophilic organisms.^[^ ^36^, ^37^, ^38^, ^39^ ^]^ It functions by converting superoxide into hydrogen peroxide and plays a key role in the defense mechanism of the organism. In the superoxide reductase active site, the iron(II) ion is surrounded and bound to the side chains of four histidine‐derived nitrogen donors arranged in a planar fashion, whereas a cysteinate ligand is positioned on the axial site, designated as an N_4_S coordination environment of the iron(II) ion. This configuration results in a square pyramidal geometry around the iron center that resembles the one seen in heme enzymes, such as the cytochrome P450s, where the iron center is coordinated by four pyrrolyl nitrogen donors of the heme in the equatorial plane with a cysteinate ligand in the axial position. Both enzymes are believed to engage similar iron‐(hydro)peroxo intermediates during their catalytic processes.^[^ ^1^, ^2^, ^3^, ^4^, ^5^, ^6^, ^7^, ^8^, ^9^, ^10^, ^11^, ^12^, ^13^, ^36^ ^]^ However, these intermediates undergo significantly different outcomes: in superoxide reductase, the hydroperoxo ligand is protonated at the proximal oxygen atom to form H_2_O_2_ products, while in the P450, the protonation is on the distal oxygen atom and thereby leads through heterolytic O─O bond cleavage to the formation of Compound I and a water molecule. It is believed that the protonation machinery and the hydrogen bonding network inside the protein structure of the P450s avoid the “shunt” pathway leading to H_2_O_2_ and form Compound I only.^[^ ^1^, ^2^, ^3^, ^4^ ^]^

**Figure 1:**
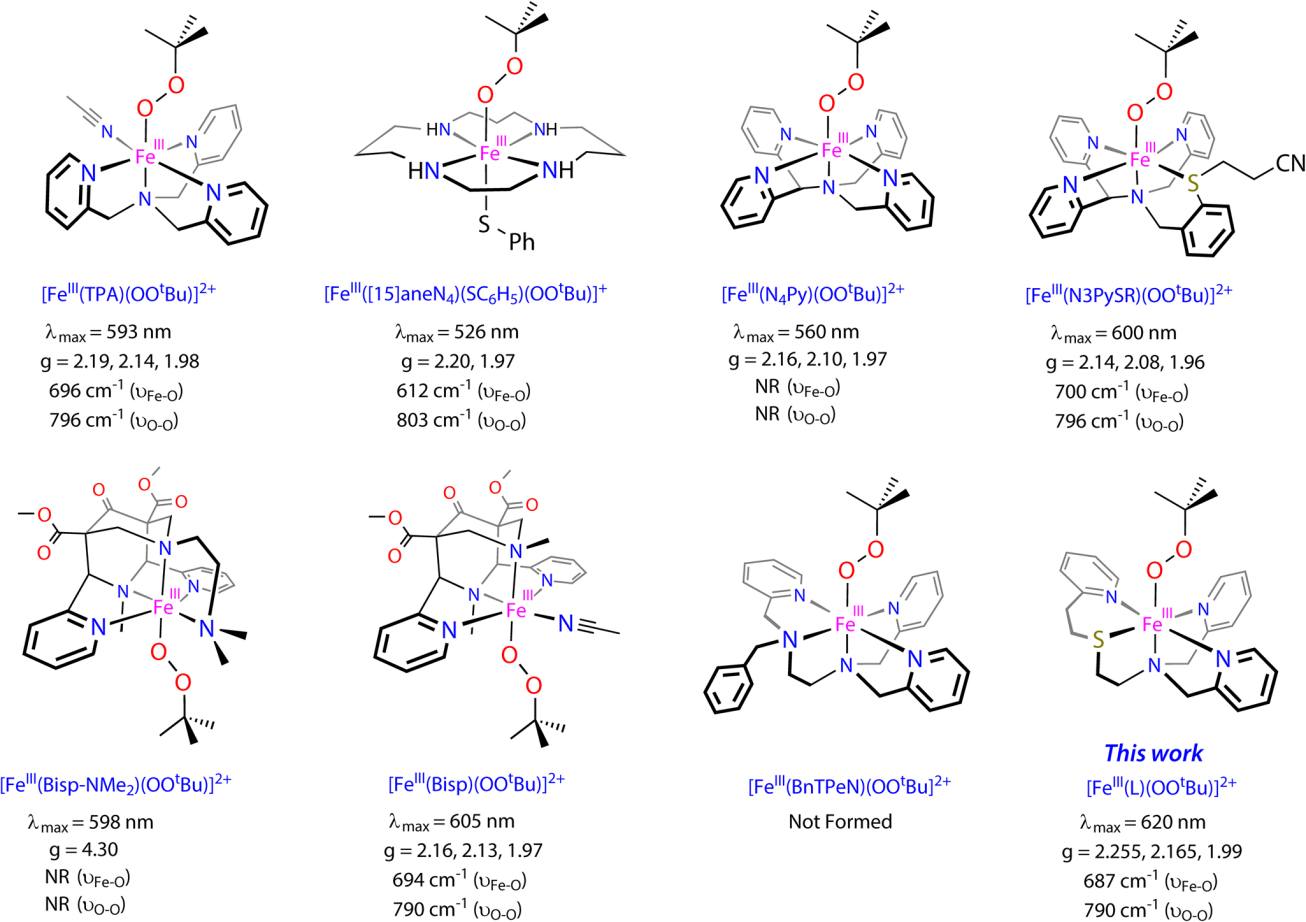
Structures and spectroscopic properties (UV–vis absorption *λ*_max_, EPR *g*‐values, and key stretching frequencies) of various nonheme iron(III)‐OOH/OO*^t^* Bu intermediates reported in the literature. Ligand names: TPA = tris(pyridyl‐2‐methyl)amine, N4Py = *N*,*N*‐bis(2‐pyridylmethyl)‐*N*‐bis(2‐pyridyl)methylamine, Bisp = Bispidine, L = 2‐((2‐(pyridin‐2‐yl)ethyl)thio)‐*N*,*N*‐bis(pyridin‐2‐ylmethyl)ethan‐1‐amine), BnTPen = *N*_1_‐benzyl‐*N*_1_,*N*_2_,*N*_2_‐tris(pyridine‐2‐ylmethyl) ethane‐1,2‐diamine. NR = Not Reported.

As enzymatic catalytic cycle intermediates are short‐lived, biomimetic models have been created that contain the first coordination sphere of the metal active site of the enzyme but are devoid of the protein environment.^[^ ^17^, ^21^, ^22^, ^23^, ^40^, ^41^, ^42^, ^43^, ^44^, ^45^, ^46^, ^47^, ^48^, ^49^, ^50^ ^]^ Biomimetic models enable studies on the structure, spectroscopic characterization, and chemical properties of short‐lived metal complexes and help with the identification and characterization of the analogous enzymatic catalytic cycle intermediates. There have been several reports in the literature on the spectroscopic characterization of biomimetic model complexes of nonheme iron(III)‐alkylperoxo complexes and their reactivity patterns and a representative set of complexes are shown in Figure 1. Interestingly, these iron(III)‐alkylperoxo complexes show distinct spectroscopic parameters depending on the metal‐ligand system, whereby the maximum absorption in the UV–vis spectrum varies from *λ*_max_ = 526–605 nm.^[^ ^51^, ^52^, ^53^, ^54^, ^55^ ^]^ Several of the complexes were also characterized with resonance Raman spectroscopy and their Fe─O and O─O stretch vibrations were identified, which span a range from 611 to 700 cm^−1^ for the *ν*_Fe‐O_ mode and from 790 to 868 cm^−1^ for the *ν*_O‐O_ vibration. Despite the fact that the heme iron(III)‐hydroperoxo complexes tend to be sluggish oxidants, the nonheme iron(III)‐hydroperoxo complexes, by contrast, have been shown to be reactive with a selection of substrates. It was hypothesized that this difference is as a result of homolytic O─O bond cleavage in nonheme iron(III)‐hydroperoxo, whereas in heme systems typically heterolytic O─O bond cleavage takes place.^[^ ^55^ ^]^ These studies have prompted the hypothesis that a low‐spin state in the iron(III)‐hydroperoxo intermediate in the P450s promotes heterolytic O─O bond cleavage, while the corresponding high‐spin intermediate in superoxide reductase facilitates Fe─O bond cleavage.^[^ ^53^ ^]^ Our group has recently demonstrated that an O─C bond cleavage in a nonheme iron(III)‐alkylperoxo complex can take place with a pentadentate bispidine ligand framework resulting in an iron(III)‐superoxo species.^[^ ^56^ ^]^ To exploit this success further and gain insight into the reactivity patterns, we decided to follow up the work with a detailed characterization and reactivity study.

**Figure 2:**
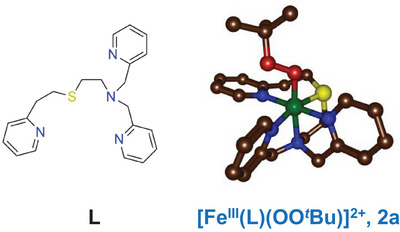
Ligand L and DFT‐optimized structure of the intermediate **2a** investigated in this work.

Many synthetic model systems have been reported toward understanding the potential reactivity of iron(III)‐hydroperoxo and iron(III)‐alkylperoxo complexes.^[^ ^51^, ^52^, ^53^, ^54^, ^55^, ^56^, ^57^, ^58^, ^59^, ^60^, ^61^, ^62^, ^63^, ^64^, ^65^, ^66^, ^67^, ^68^, ^69^, ^70^, ^71^, ^72^, ^73^, ^74^, ^75^, ^76^, ^77^ ^]^ Thus, the low‐spin [Fe^III^([15]aneN_4_)(SC_6_H_5_)(OO*^t^* Bu)]^+^, [Fe^III^(N3PySR)(OO*^t^* Bu)]^+^, and [Fe^III^(Bisp)(OO*^t^* Bu)]^2+^ complexes were found to be unreactive with model substrates under ambient conditions.^[^ ^57^, ^58^, ^59^, ^60^ ^]^ The precise mechanisms governing the reactivity and activity of iron(III)‐alkylperoxo complexes remain elusive due to their thermal instability. Moreover, the kinetic studies of such species are often ambiguous because of bifurcation reaction pathways leading to iron(IV)‐oxo complexes. Over the past decades, reports have emerged of either homolytic or heterolytic O─O bond cleavage in iron(III)‐alkylperoxo complexes.^[^ ^52^, ^56^, ^73^, ^74^, ^75^, ^76^, ^77^ ^]^ Several biomimetic model complexes with thiolate‐ligated nonheme iron(III)‐alkylperoxo have been characterized spectroscopically,^[^ ^52^, ^54^, ^56^ ^]^ however, none of these show any oxidative reactivity.^[^ ^53^, ^54^, ^73^, ^74^, ^75^, ^76^, ^77^ ^]^ Herein, we report the synthesis, characterization, and reaction kinetics of a low‐spin iron(III)‐alkylperoxo complex (Figure 2), which shows reactivity toward organic substrates driven by a general O─O bond cleavage pathway. We provide the first single turnover study of iron(III)‐alkylperoxo complexes and report reaction kinetics and show these complexes to even be able to perform the abstraction of a hydrogen atom from an aldehyde C─H group.

## Results and Discussion

The pentadentate ligand framework BnTPen (BnTPen = *N*_1_‐benzyl‐*N*_1_,*N*_2_,*N*_2_‐tris(pyridine‐2‐ylmethyl)ethane‐1,2‐diamine) is a well‐known ligand structure that has been shown to stabilize high‐valent iron(IV)‐oxo and iron(III)‐peroxo complexes.^[^ ^78^, ^79^, ^80^ ^]^ However, this ligand structure does not facilitate the generation of the iron(III)‐alkylperoxo species, whereas the structure has been reported for other well‐known ligand systems such as N4Py, N3PySR, and Bisp‐NMe_2_ (see Figure 1 for selected examples).^[^ ^51^, ^52^, ^53^, ^54^, ^55^ ^]^ In order to overcome this problem, we decided to modify the ligand architecture to enable greater stability to the iron(III)‐alkylperoxo intermediate. In particular, this was achieved by integrating a pendant arm into the ligand framework attached to a thioether group. Thus, we synthesized a new pentadentate ligand (designated ligand L and shown in Figure 2), namely L = 2‐((2‐(pyridin‐2‐yl)ethyl)thio)‐*N*,*N*‐bis(pyridin‐2‐ylmethyl)ethan‐1‐amine. The detailed synthetic procedure of L and its analytical characterization is provided in the Supporting Information, whereby NMR and electrospray ionization mass spectrometry (ESI‐MS) measurements on the ligand are provided in Figures S1–S3. The obtained spectra match those reported from the literature for ligand L well.^[^ ^81^ ^]^

**Figure 3:**
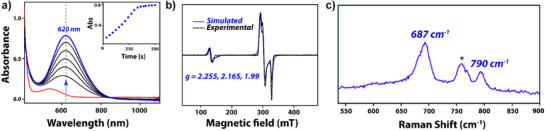
a) Time‐dependent UV–vis absorption spectrum for the formation of **2a** (in blue) from **1a** (in red) at 233 K in acetonitrile with 30 equiv of *^t^* BuOOH (the inset shows the time trace at the absorption maximum of 620 nm). b) EPR spectrum of **2a**, experimental (black dotted lines) and simulated (blue lines) in acetonitrile at 77 K. The EPR simulation was done using Easyspin. c) Resonance Raman spectrum of **2a** at 233 K at 638 nm excitation wavelength. * indicates a solvent peak.

Subsequently, the ligand L was reacted with [Fe^II^(CH_3_CN)_2_(OTf)_2_] with OTf = triflate, in dry acetonitrile under an inert atmosphere inside a glovebox that formed the corresponding iron(II) complex: [Fe^II^(L)(CH_3_CN)](OTf)_2_ (**1a**). The iron(II) complex **1a** was characterized with UV–vis absorption spectroscopy, cyclic voltammetry (CV), NMR spectroscopy, and ESI‐MS, see Figures S4–S8. The UV–vis spectrum of the iron(II) complex **1a** displays a ligand‐to‐metal charge‐transfer band with a molar extinction coefficient (*ε*) and *λ*_max_ values with *ε* **_1a_** = 2200 M^−1^ cm^−1^ (*λ*_max,_ **_1a_** = 358 nm) along with a smaller band at 540 nm, *ε* = 126 M^−1^ cm^−1^, which probably is due to sulfur‐to‐iron charge‐transfer, see Figures 3a and S7. Moreover, cyclic voltammetry studies of the iron(II) complex **1a** exhibited an irreversible or quasi‐irreversible Fe^III^/Fe^II^ couple. Therefore, a differential pulse voltametric experiment was performed to determine the redox potential of the Fe^III^/Fe^II^ couple, which is found to be at 1.12 V (Figure S8). In addition, we also performed a ^1^H NMR analysis of the complex in CD_3_CN (Figure S5), and these measurements establish the paramagnetic nature of **1a** at room temperature. The solution‐state magnetic moment was then determined using the modified Evans method. The ^2^H NMR Evans method allowed us to determine the magnetic moment of 4.87 *μ*_B_ for **1a** in a mixture of CD_3_CN and CDCl_3_ as markers at 298 K. The Evans measurement indicates that complex **1a** possesses an *S* = 2 ground state in solution (see Figure S6). This high‐spin character of the iron(II) complex verifies the irreversible/quasi‐irreversible Fe^III^/Fe^II^ couple during the cyclic voltametric study. The ESI‐MS spectrum of **1a** gives a prominent peak at *m*/*z* 569.06 corresponding to the [Fe^II^(L)(OTf)]^+^ molecular ion fragment of **1a**, which was confirmed with the isotopic distribution patterns of the fragment ions (Figure S4).

When the spectroscopic properties of **1a** are compared with those of the well‐established [Fe^II^(BnTPen)]^2+^ complex, it is evident that the inclusion of a sulfur atom in the primary coordination sphere of the complex has changed the ground state spin of the iron(II) complex from a multiplicity of *S* = 0 to *S* = 2. Furthermore, changes are observed in the features of the differential pulse voltammetry spectra for the Fe^II/III^ couple, whereby **1a** gives an irreversible peak that is shifted upwards by 250 mV as compared to [Fe^II^(BnTPen)]^2+^. This redox couple shift should bring about significant changes in the properties of high‐valent intermediates.

When **1a** was treated with *tert*‐butyl‐hydroperoxide (*^t^* BuOOH, 30 equiv in acetonitrile, 233 K), over time it gradually converted into a new blue species **2a**. The complex exhibits an intense absorption band at 620 nm (*ε* **_2a_** ≈ 840 M^−1^ cm^−1^, see Figure 3a). The absorption maximum and the molar absorption coefficient confirm the formation of [Fe^III^(L)(*^t^* BuOO)]^2+^ with values characteristic for an alkylperoxo to Fe^III^ LMCT band.^[^ ^82^, ^83^ ^]^ Thanks to the long half‐life of 3 h for **2a**, it exhibits sufficient stability for spectroscopic analyses with UV–vis, EPR, and resonance Raman spectroscopy, as well as ESI‐MS. The positive ion mode ESI‐MS spectrum of **2a** shows a major peak at *m*/*z* 658.11 that corresponds to the molecular ion with stoichiometry [Fe^III^(L)(*^t^* BuOO)(OTf)]^+^ (see Figures S9 and S10 with the assignments confirmed from their isotopic distribution patterns). Furthermore, the mass spectrum of **2a** matches well with simulated mass spectra.

The X‐band EPR spectrum of a frozen solution of **2a** was measured at 77 K. The EPR spectrum shows three well‐resolved signals in the *g* = 2 region, see Figure 3b. In addition, there is a weak signal observed around 150 mT that is attributed to the formation of a high‐spin iron(III)‐hydroxo species, presumably formed from the decomposition of **2a**. The intensity of the low‐field signal increases with the concomitant decrease in the EPR signals observed in the *g* = 2 region over a period of 2 h, see Figure S12. This indicates that the signal arising in the *g* = 2 region exclusively originates from the reactive species **2a**. Excellent agreement between the experimental spectrum of **2a** and its simulation was obtained for the following modelling conditions: *S* = ½, *g* = [2.255, 2.165, 1.990], and using a weight of 99.9% (Figure 3b).^[^ ^84^ ^]^ By contrast, the low‐field signal observed for the high‐spin iron(III)‐hydroxo complex could be simulated using a *g*‐value of 4.97 with a weightage of 0.1%. The spin Hamiltonian parameters derived for **2a** evidently suggest a low‐spin signature associated with the in situ formed iron(III)‐alkylperoxo complex (**2a**), which is consistent with earlier reports on analogous complexes.^[^ ^52^, ^53^, ^54^ ^]^

The resonance Raman spectrum of **2a** obtained at the 638 nm excitation wavelength features two resonantly enhanced bands at 687 and 790 cm^−1^ (Figure 3c) that are absent in the spectra for the parent iron(II) complex and the one for pure solvent. We assign the resonance Raman bands as originating from the Fe^III ^− O (687 cm^−1^) and O─O (790 cm^−1^) stretch vibrations. The band assignments were made based on comparisons with analogous low‐spin Fe(III)‐OOH/R complexes reported in the literature shown in Figure 1 above.^[^ ^52^, ^53^, ^54^ ^]^ The magnitude of these vibrations implicates the presence of a strong Fe─O bond and a weak O─O bond. Notably, the O─O stretching frequency was observed near 800 cm^−1^, a region characteristic of low‐spin Fe(III)‐OOH/R species.^[^ ^64^, ^65^, ^66^, ^67^, ^68^, ^69^, ^70^, ^71^, ^72^ ^]^ This observation, in conjunction with resonance Raman (rR) literature and the EPR data of **2a**, unequivocally confirms that **2a** exists in a low‐spin state (*S* = ^1^/_2_).

**Scheme 1:**
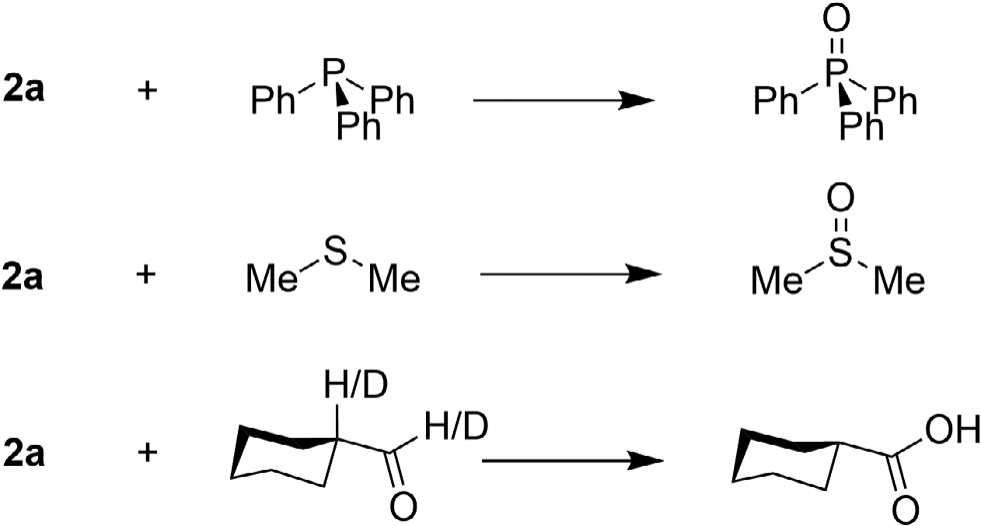
Reactions of **2a** with selected substrates studied in this work.

The stability of **2a** at 233 K encouraged us to investigate its reactivity with selected organic substrates, see Scheme 1. The addition of organic substrates to the mixture drastically increased the rate of decay of the corresponding LMCT band in several cases, thereby supporting the reaction of the substrates with complex **2a**.

We started the reactivity studies by adding triphenylphosphine as a model substrate for studies on oxygen atom transfer reactions as this substrate forms a strong P═O bond and consequently should be able to react with relatively weak oxidants.^[^ ^85^, ^86^ ^]^ The addition of the excess equivalent of triphenylphosphine to the reaction mixture leads to the decomposition of **2a** under pseudo‐first‐order reaction conditions, as monitored by the decay of the 620 nm band in the UV–vis spectrum (Figure S13). By studying the reaction of **2a** with different concentrations of triphenylphosphine, a linear dependence between *k*_obs_ and the triphenylphosphine concentration was obtained at 233 K in MeCN. From the correlation of the rate constants versus the concentration, the second‐order rate constant *k*_2_ = 1.48 M^−1^ s^−1^ was determined (Figure S14).

Subsequently, to test the substrate scope and oxidative capabilities of **2a**, we investigated its reaction with alternative substrates as shown in Scheme 1. No kinetic decay of the 620 nm band was detected when **2a** was mixed with thioanisole at 233 K under single turnover conditions; however, when the reaction was carried out under catalytic conditions at 298 K, we did observe the formation of sulfoxide as a product but were unable to measure rate constants (Figure S24). In order to obtain reaction kinetics parameters, we then tested dimethylsulfide as a model substrate for its reactivity with **2a** at 233 K and were able to extract kinetics information. Thus, the reaction of **2a** with dimethylsulfide was monitored with different substrate concentrations in the reaction mixture to obtain a linear dependence between *k*_obs_ and the concentration of dimethylsulfide at 233 K. A second‐order rate constant for the sulfoxidation reaction of **2a** with dimethylsulfide of *k*_2_ = 1.54 × 10^−2^ M^−1^ s^−1^ was determined (Figures S16 and S19). As such, **2a** reacts through heteroatom oxidation of triphenylphosphine and sulfides like dimethylsulfide.

**Figure 4:**
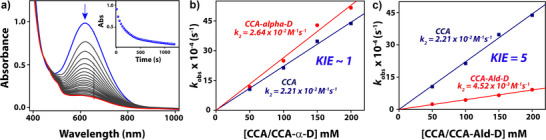
a) UV–vis spectral changes of **2a** (1 mM) upon addition of 100 equiv of CCA in MeCN at 233 K. The inset shows the decay profile of the 620 nm band. b) Second‐order rate constant determined for the reaction of **2a** (1 mM) with different concentrations of CCA and α‐[D_1_]‐CCA in MeCN at 233 K. c) Second‐order rate constant determined for the reaction of **2a** (1 mM) with different concentrations of CCA and Ald‐[D_1_]‐CCA in MeCN at 233 K.

**Scheme 2:**
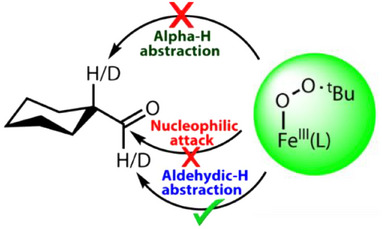
Possible reaction sites between CCA and **2a**.

Generally, metal‐dioxygen intermediates that include peroxide, hydroperoxide, and alkylperoxide complexes act as efficient aldehyde deformylating agents.^[^ ^87^, ^88^, ^89^, ^90^, ^91^, ^92^, ^93^, ^94^, ^95^, ^96^, ^97^, ^98^ ^]^ The majority of these aldehyde deformylation reactions proceed via direct nucleophilic attack by the oxidant at the electrophilic carbonyl carbon atom. However, an alternative electrophilic pathway was suggested for manganese(III)‐peroxo and copper(II)‐peroxo complexes wherein the rate‐limiting step was C_α_─H bond cleavage in the substrate instead.^[^ ^91^, ^92^, ^93^, ^94^, ^95^ ^]^ These studies proposed that after hydrogen atom abstraction a keto‐enol tautomerism may trigger a proton shuttle mechanism in the case of a hydrogen atom being present at the C_α_─H position to the carbonyl group. To establish the nature of reactivity of the iron(III)‐alkylperoxo complex in the current study, we performed a thorough kinetics investigation along with product analysis with cyclohexanecarboxaldehyde (CCA) as a model substrate. Upon addition of CCA to **2a** in MeCN at 233 K, the intermediate decayed rapidly (Figure 4a). The rate of decay of the 620 nm band in the UV–vis spectrum increased linearly as a function of CCA concentration, indicating a bimolecular reaction mechanism. The resulting second‐order rate constant (*k*_2_) for the oxidation of CCA by **2a** at 233 K is 2.21 × 10^−2^ M^−1^ s^−1^ (Figure 4b). The product analysis showed the formation of cyclohexanecarboxylic acid products, which would imply a reaction mechanism starting with aldehyde C─H abstraction followed by OH rebound to form cyclohexanecarboxylic acid products. As shown previously,^[^ ^95^ ^]^ the C_α_─H bond is weaker than the aldehyde C─H bond and hence C_α_─H abstraction should be the dominant pathway in the reaction of CCA with an oxidant. However, recent work with 2‐phenylpropionaldehyde showed that the stronger aldehyde C─H bond can be cleaved if the substrate‐oxidant noncovalent interactions stabilize the transition state structures.^[^ ^98^ ^]^ Therefore, we used kinetic isotope effect (KIE) measurements as a tool to mitigate the conflict of the reaction pathways. Replacement of the C_α_─H group in CCA by deuterium acts as a mechanistic probe to identify the plausible rate‐determining step. Under identical conditions when α‐[D_1_]‐CCA was used as a substrate, a second‐order rate constant of 2.64 × 10^−2^ M^−1^ s^−1^ was obtained (Figure 4b). As such, the ratio of the rate constants of **2a** with CCA and α‐[D_1_]‐CCA is close to 1. Therefore, the absence of a significant KIE for H/D replacement rules out the possibility of a rate‐determining electrophilic C_α_─H bond cleavage step during the reaction process (Scheme 2).

To explore the potential nucleophilic oxidation pathway for the oxidation of CCA and gain insight on the electronic configuration of the transition states, a series of *para*‐substituted benzaldehyde substrates were used to enable a Hammett analysis. In the nucleophilic pathway, the oxidant attacks the carbonyl group leading to the formation of the iron‐alkoxide adduct, which should result in a positive slope in a Hammett plot. However, upon addition of excess amounts of various *para*‐substituted benzaldehyde substrates to **2a** a Hammet plot with *negative* slope was obtained. Thus, the addition of substrate to the reactant mixture led to the disappearance of the band at 620 nm in the UV–vis spectrum and enabled us to determine a Hammett plot with a range of substrates. The plot of the reaction rates of **2a** with *para*‐substituted benzaldehyde substrates against their *para*‐substituent constants (*σ*_p_) produces a Hammett plot with a *negative* slope (*ρ* = −0.98). This result is opposite of what is typically observed for a nucleophilic attack of an oxidant at a carbonyl group (Figure S17). In fact, a negative Hammett slope typically represents an electrophilic reaction pathway instead.^[^ ^99^ ^]^ Therefore, the experiments presented in this work rule out a nucleophilic reaction mechanism between **2a** and aldehydes (Scheme 2).

To further establish the electrophilic nature of the oxidant, we also performed the reaction of **2a** with aliphatic aldehydes. In particular, the electrophilicity of **2a** in the reaction with aldehydes was investigated by employing a primary aldehyde (valeraldehyde for 1°‐CHO), a secondary aldehyde (2‐methylbutyraldehyde for 2°‐CHO), and a tertiary aldehyde (trimethylacetaldehyde for 3°‐CHO) and rate constants were measured for each system. The order of the reaction rates (*k*_2_) was observed to be 3°‐CHO > 2°‐CHO > 1°‐CHO, which supports the electrophilic character of **2a** in the oxidation reaction of aldehydes (see Figures S18 and S21; Table S2).

After we ruled out the electrophilic C_α_─H abstraction and the nucleophilic iron alkoxide adduct formation, the only plausible pathway for the electrophilic cyclohexane carboxaldehyde oxidation to form cyclohexane carboxylic acid would involve C─H abstraction of the aldehydic‐hydrogen atom. To confirm a rate‐determining aldehyde C─H abstraction pathway, we employed a substrate with deuterated aldehyde group, i.e., ald‐[D_1_]‐CCA. The rate constant ratio of **2a** with CCA versus ald‐[D_1_]‐CCA resulted in a classical KIE value of 5 under identical reaction conditions, see Figure 4c. Therefore, the reaction happens via a rate‐determining aldehyde C─H hydrogen atom abstraction. This is a surprising result as previous calculations on the C─H bond dissociation energies of the C_α_─H and C_ald_─H bonds of CCA in the gas phase found values of Δ*G* = 72.1 and 79.5 kcal mol^−1^,^[^ ^95^ ^]^ and hence under ideal circumstances the C_α_─H atom from CCA should be abstracted by the oxidant first. The fact that this does not happen here must be the result of stereochemical repulsions and poor access to the C_α_─H atom by the oxidant. The KIE values and rate constants obtained for CCA activation by **2a** reported here are very similar to those obtained for the oxidation of aldehydes by Fe^IV ^= O intermediates.^[^ ^96^, ^97^, ^98^ ^]^ To find out whether **2a** converts into an iron(IV)‐oxo species prior to its reaction with aldehydes radical trapping experiments were performed. Thus, we obtained the product, cyclohexane carboxylic acid, from the reaction of **2a** with cyclohexane carboxaldehyde in the presence of the radical trapping reagent TEMPO. The same products were obtained in a reaction without the radical trapping agent, see Figure S22. These results demonstrate unequivocally that the *tert*‐butoxy radical was not involved in the oxygen atom transfer reaction and reactivity must have originated from a short‐lived iron(IV)‐oxo species.

To gain further understanding into the mechanism of substrate oxidation and the electronic structure of the short‐lived intermediates originating from [Fe^III^(L)(OO*^t^* Bu)]^2+^ (**2a**), a series of computational studies were conducted. We started with geometry optimizations of complex **2a** in the doublet, quartet, and sextet spin states. At the UBP86/BS3 level of theory, the doublet spin state is the ground state in agreement with EPR measurements reported above in Figure 3b. Although all three spin states were considered for all mechanistic calculations reported in this work (see Supporting Information for details), we will focus on the lowest energy doublet spin results only as experimental work assigned complex **2a** as a doublet spin ground state. In the doublet spin state (^2^ **2a**), the system has a single singly occupied molecular orbital designated π*_yz_ that represents the antibonding interactions of the iron 3d_yz_ orbital with the π*_OO_ orbital on the peroxo group. As a consequence, single occupation of π*_yz_ in ^2^ **2a** gives considerable unpaired spin density on the two oxygen atoms. The Fe─O_p_ distance is 1.799 Å in ^2^ **2a**, while the O─O distance and Fe─S distances calculated at UBP86‐GD3BJ/BS3 level of theory are 1.455 and 2.272 Å, respectively. These distances are typical for calculated Fe─S interactions and seen before in, e.g., cytochrome P450 Compound I or cysteine dioxygenase enzyme calculations.^[^ ^100^, ^101^, ^102^, ^103^, ^104^, ^105^, ^106^, ^107^, ^108^, ^109^, ^110^, ^111^, ^112^, ^113^, ^114^, ^115^ ^]^

**Figure 5:**
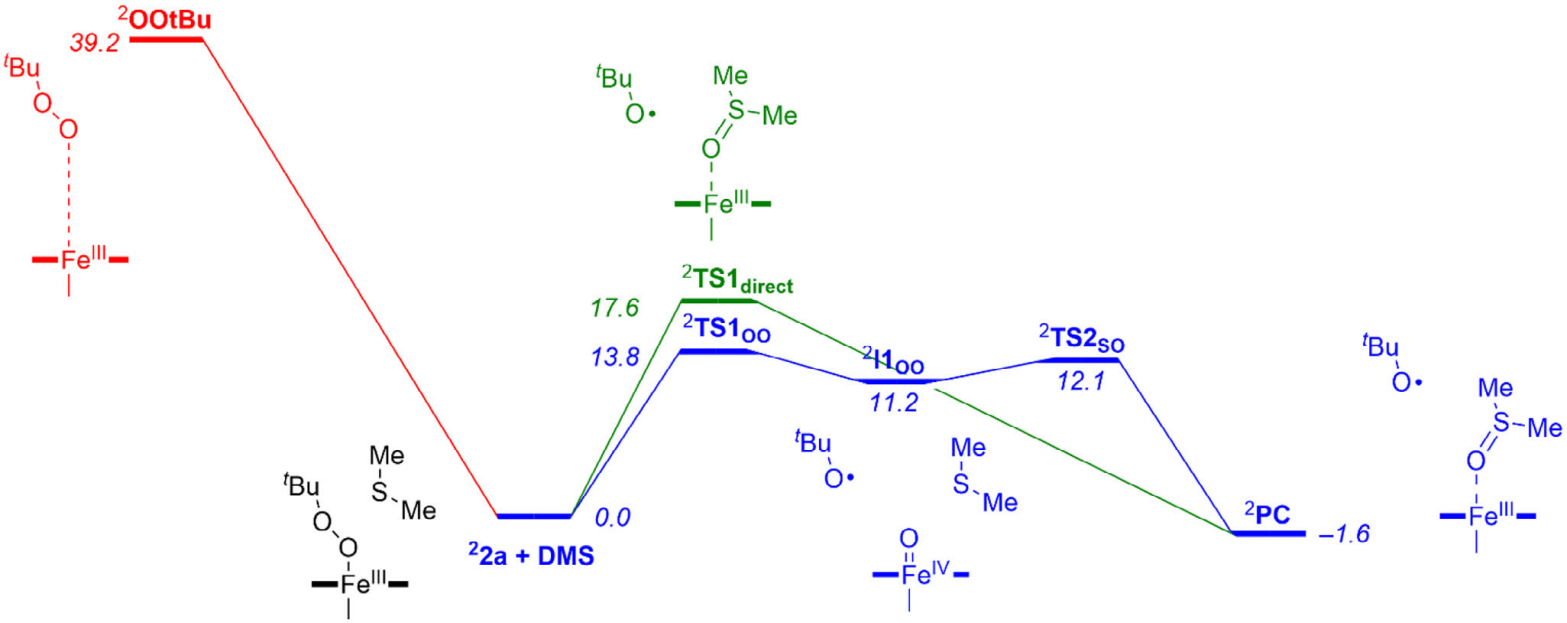
UBP86‐GD3/BS3 calculated free energy landscape (in kcal mol^−1^ with data obtained at 233 K with solvent, zero‐point, thermal, and entropic corrections included) for the reaction of ^2^ **2a** with DMS either through direct attack by the iron(III)‐alkylperoxo species on sulfur or through a stepwise reaction via an iron(IV)‐oxo species.

Next, dimethylsulfide was added to the optimized geometries of ^2,4,6^ **2a** and the substrate sulfoxidation reaction pathways were calculated either directly from the iron(III)‐alkylperoxo state or after initial O─O bond cleavage to form an iron(IV)‐oxo species. Figure 5 displays the free energy landscape of the direct and stepwise mechanisms of dimethylsulfide (DMS) sulfoxidation by ^2^ **2a**, while the remaining spin state structures are given in the Supporting Information. First, we explored the possibility of the iron(III)‐alkylperoxo species to acting as a potential oxidant for the heteroatom transfer reaction. The direct reaction of DMS with ^2^ **2a** involves a significant free energy of activation of Δ*G* ^‡^ = 17.6 kcal mol^−1^. Thereafter, we tested the possibility of the formation of an iron(IV)‐oxo species through homolytic O─O bond cleavage in ^2^ **2a** via ^2^ **TS1_OO_** to form an iron(IV)‐oxo species with a *tert*‐butoxyl radical (structure **I1**). This pathway has a free energy of activation of Δ*G* ^‡^ = 13.8 kcal mol^−1^ via ^2^ **TS1**_OO_ and results in a shallow local minimum for an iron(IV)‐oxo species at Δ*G* = 11.2 kcal mol^−1^ with respect to the reactants complex. The modest barrier and the fact that it is lower in free energy than the competitive direct sulfoxidation barrier implicate a feasible homolytic O─O bond cleavage under room temperature conditions. However, as the free energy of the iron(IV)‐oxo species **I1** is above that of the iron(III)‐alkylperoxo complex, the thermodynamic equilibrium between the two states will revert the complex back to the more stable iron(III)‐alkylperoxo species unless there are substrate molecules in the direct vicinity that can trigger the sulfoxidation reaction. Indeed, in a solution without substrate present, we were unable to trap and characterize the iron(IV)‐oxo complex experimentally in acetonitrile, which is consistent with an endergonic reaction step for its formation. Nevertheless, once structure **I1** is formed and substrate is available in the solvent cage or second coordination sphere, an oxygen atom transfer reaction to substrate may occur via transition state **TS2**_SO_ to produce the sulfoxide products. The sulfoxidation reaction; however, is exergonic and releases 1.6 kcal mol^−1^ in the doublet spin state with respect to a reactant complex of ^2^ **Re**.

Therefore, the calculations show that the formation of the iron(IV)‐oxo species from an iron(III)‐alkylperoxo complex is endothermic, which implies that the O─O cleavage of the iron(III)‐alkylperoxo complex is challenging and the energy required to cleave the O─O bond is large. Consequently, it will be difficult for this system to populate the iron(IV)‐oxo complex in significant amounts to trigger oxygen atom transfer. However, if substrate is present in large concentrations and easily accessible to the short‐lived iron(IV)‐oxo species, for instance, when it is bound inside the solvent cage of the reactant complex, a reaction leading to products can occur. The binding of substrate to the solvent cage of complex **2a** will be dependent on the polarity of the substrate and its ability to form stable noncovalent interactions. With DMS as a substrate an exergonic overall sulfoxidation is calculated whereby the iron(IV)‐oxo is expected to rapidly react with DMS. As such, the alkylperoxo complex discussed in this work is a sluggish oxidant due to a thermodynamically uphill reaction to form an iron(IV)‐oxo species. That will mean the reverse reaction leading back to the iron(III)‐alkylperoxo species may dominate unless a substrate molecule is available in the vicinity of the iron(IV)‐oxo complex. In agreement with previous work, the barriers for direct reactivity of the iron(III)‐alkylperoxo complex with substrates are high and may not be competitive with the lower pathways for O─O bond cleavage.^[^ ^57^, ^70^, ^71^, ^116^, ^117^ ^]^

To test whether OO*^t^* Bu could be involved in the chemical reaction, we ran a constraint geometry scan for Fe─O bond elongation from structure **2a**. The energy of that scan steeply rises to well over 30 kcal mol^−1^. Therefore, release of OO*^t^* Bu from **2a** is an endergonic process and incurs a significant amount of free energy as compared to the alternative O─O bond cleavage pathway. Hence, the Fe─O bond cleavage mechanism will not be able to compete with the pathway leading to the iron(IV)‐oxo species, which is considerably lower in free energy.

**Figure 6:**
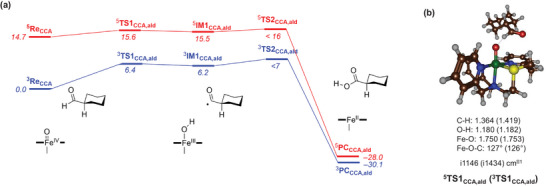
a) UBP86‐GD3/BS3 calculated free energy landscape (in kcal mol^−1^ with data obtained at 233 K with solvent, zero‐point, thermal, and entropic corrections included) for the reaction of the iron(IV)‐oxo species **2b** with cyclohexanecarboxaldehyde (CCA) for substrate hydroxylation of the aldehyde group (in blue and red). b) Optimized geometries of ^5,3^ **TS1**_CCA,ald_ with bond lengths in Å, angles in degrees and the imaginary frequency in cm^−1^.

To further establish reactivity of **2a** as a hydrogen atom abstraction agent, we tested its reaction with CCA as a substrate by calculating the mechanism from the iron(IV)‐oxo species, see Figure 6. In agreement with previous experimental and computational studies of analogous complexes,^[^ ^45^, ^46^, ^47^, ^48^, ^49^, ^50^, ^73^, ^74^, ^75^, ^76^, ^77^, ^78^, ^79^, ^80^, ^118^, ^119^, ^120^ ^]^ the triplet spin state is the ground state. The hydrogen atom abstraction barrier is small and a free energy of activation of Δ*G* ^‡^ = 6.4 kcal mol^−1^ via ^3^ **TS1**_CCA,ald_ with respect to ^3^ **Re**_CCA_ is obtained, whereas on the quintet spin state surface the barrier is calculated at Δ*G* = 15.6 kcal mol^−1^. On both spin state surfaces, the radical intermediate **IM1** is short‐lived and collapses to the product complexes with negligible barriers. The alternative C_α_─H hydrogen atom abstraction from CCA was also tested but found to be several kcal mol^−1^ higher in free energy. Therefore, **2a** will react with CCA through regioselective aldehyde C─H hydrogen atom abstraction to form cyclohexane carboxylic acid products. The calculated reaction mechanism and products are in agreement with the experimental kinetics that could not identify an iron(IV)‐oxo intermediate but showed a rapid conversion to cyclohexane carboxylic acid product formation.

Optimized transition state geometries for aldehyde C─H abstraction are shown in Figure 6b. Both transition states are late on the potential energy surface with longer C─H than O─H distances. The substrate position with respect to the Fe─O axis is somewhat bent in both transition states with an Fe─O─C angle of about 126°. Note that in the transition state structures there is a hydrogen bond between the carbonyl oxygen atom of the substrate with one of the protons of the ligand. This hydrogen bond will stabilize the transition state and force the structure in a more bent orientation. Both transition states have a large imaginary frequency (>1100 cm^−1^), which will result in a significant amount of quantum chemical tunneling and a large kinetic isotope effect.

The transition state ordering of aldehyde C─H abstraction below those for C_α_─H abstraction is unexpected as normally substrate activation will result in the cleavage of the weakest C─H bond. In CCA, the weakest C─H bond is the C_α_─H bond rather than the aldehyde C─H bond, and hence substrate hydroxylation at the C_α_─H position may be expected. However, the weak intermolecular interaction between the substrate carbonyl and protons of the ligand scaffold stabilize the aldehyde C─H abstraction transition states and change the reaction selectivity. As such, weak noncovalent interactions in the second coordination sphere of the complex stabilize the aldehyde C─H abstraction transition states and guide the reactions to the unexpected aldehyde hydroxylation mechanism. This is similar to the reactions observed between iron(IV)‐oxo and iron(III)‐peroxo complexes with 2‐phenylpropionaldehyde that also gave aldehyde C─H abstraction rather than C_α_─H abstraction as a result of weak interactions between oxidant and substrate that stabilized the aldehyde C─H abstraction pathways.^[^ ^98^ ^]^

## Conclusion

In this work, we report the synthesis and characterization of a sulfur‐ligated, low‐spin (*S* = ½) iron(III)‐alkylperoxo complex supported by a pentadentate N_4_S ligand framework. The complex was characterized using a variety of spectroscopic methods including UV–vis absorption, resonance Raman, and EPR spectroscopy, as well as ESI‐MS. Mechanistic studies of the iron(III)‐alkylperoxo species with substrates shows versatile reactivity patterns through multiple pathways, including homolytic or heterolytic O─O bond cleavage, Fe─O bond cleavage, as well as C─O bond cleavage, leading to different products. In biomimetic chemistry, generating an iron(IV)‐oxo species from an iron(III)‐alkylperoxo intermediate has always been particularly challenging, especially with pentadentate ligands. Our combined experimental and computational studies elucidate the reasons behind this limitation. Specifically, the conversion of iron(III)‐alkylperoxo to iron(IV)‐oxo is found to be endothermic, with the equilibrium favoring the iron(III)‐alkylperoxo species. As a result, in biomimetic systems, artificial oxidants such as PhIO and H_2_O_2_ should typically be employed to access high‐valent iron(IV)‐oxo species for pentadentate ligand systems. Furthermore, this study reveals that iron(III)‐alkylperoxo complexes are sluggish in oxygen atom transfer reactions unless homolytic O─O bond cleavage occurs. The reactivity of complex **2a** was examined with a series of representative substrates to probe both electrophilic and nucleophilic pathways. Notably, **2a** displays unique electrophilic reactivity with aldehydes, unlike the more common nucleophilic behavior observed for the corresponding metal‐peroxo complexes. A detailed experimental and mechanistic investigation confirms hydrogen atom abstraction from the aldehyde group of cyclohexanecarboxaldehyde, leading to the formation of cyclohexanecarboxylic acid. This reactivity is attributed to transition state stabilization enabled by favorable interactions between the substrate and the ligand framework.

## Supporting Information

The authors have cited additional references within the Supporting Information.^[^ ^121^, ^122^, ^123^, ^124^, ^125^, ^126^, ^127^, ^128^, ^129^, ^130^, ^131^ ^]^

## Conflict of Interests

The authors declare no conflict of interest.

## Supporting information



Supporting Information

## Data Availability

The data that support the findings of this study are available in the Supporting Information of this article.
