# Supplementary material for: Mechanistic Divergence in Sulfur‐Ligated Iron(III)‐Alkylperoxo Reactivity: Aldehyde Oxidation Prevails over Deformylation
Source: Angew Chem Int Ed Engl. 2025 Sep 4;64(43):e202512839. doi: 10.1002/anie.202512839 (PMC12535397; doi:10.1002/anie.202512839)
Supplement: Supplementary file 1 — Supporting Information [file ANIE-64-e202512839-s001.pdf]

Supporting Information

Mechanistic Divergence in Sulfur-Ligated iron(III)-Alkylperoxo Reactivity: Aldehyde Oxidation Prevails Over Deformylation

Jagnyesh K. Satpathy,[a] Rolly Yadav,[a] Payal Panwar,[a] Vijaya Thangaraj,[b] Maheswaran Shanmugam,[b] Chivukula V. Sastri,*[a] and Sam P. de Visser*[a,c]

[a] Dr. J. K. Satpathy, Dr. R. Yadav, Ms. P. Panwar, Prof. C. V. Sastri, Dr. S. P. de Visser
Department of Chemistry
Indian Institute of Technology, Guwahati
Assam, 781039, India
E-mail: sastricv@iitg.ac.in
[b] Ms. V. Thangaraj & Dr. M. Shanmugam
Department of Chemistry
Indian Institute of Technology, Bombay
Powai, Mumbai, Maharastra-400 076, India
[c] Dr. S. P. de Visser
The Manchester Institute of Biotechnology and Department of Chemical Engineering
The University of Manchester
131 Princess Street, Manchester M1 7DN, United Kingdom
E-mail: sam.devisser@manchester.ac.uk

Table of Contents:

**Part I: Experimental procedures.**	**Page S3**
**Part II: Experimental characterization.**	**Page S7**
**Part III: Computational data.**	**Page S21**
**Part IV: Cartesian coordinates.**	**Page S25**

Experimental Section/Methods

Materials and methods: All chemicals were purchased from Sigma Aldrich Chemical Co., and were of the best available purity, and used without further purification unless otherwise stated. Solvents were dried according to published procedures and freshly distilled under argon gas prior to use.[30] The pentadentate ligand (L, 2-((2-(pyridin-2-yl)ethyl)thio)-*N*,*N*-bis(pyridin-2-ylmethyl)ethan-1-amine), see its structure in Figure 2 (main text). was synthesized with slight modifications from the reported procedure in the literature as described below in the section "Synthesis of the ligand".[18] The ferric-alkylperoxo complex $[Fe^{III}(L)(OOtBu)]^{2+}$ (**2a**) was synthesized in acetonitrile under ambient conditions. Cyclohexanecarboxaldehyde (CCA) was distilled prior to use to avoid the presence of acid impurities, and the purity was checked with NMR spectroscopy. Two deuterated CCA compounds were studied, namely α-[D_1]-CCA (>90%, D enriched) and Ald-[D_1]-CCA (>90%, D enriched), which were procured from RVL Scientific & Engineering Pvt. Ltd. (Lucknow, India) and its purity was also confirmed by ^{1}H and ^{2}H NMR spectroscopy.

Synthesis of the Ligand

The ligand (L) was synthesized with slight modification from a previously reported procedure (see Scheme S1).[18] Namely, 1 equiv. of 2-aminoethanethiol hydrochloride was added to a solution of 1 equiv. of 2-vinylpyridine in 60 mL of ethanol, and the mixture was heated to reflux until the solution became clear. Then 25 mL of a 1 M NaOH solution in ethanol was added and the reflux was continued for 10 min. The light-yellow clouded solution was filtered to remove NaCl, and the solution was evaporated to a yellow oil. This oil was dissolved in water, the aqueous solution was extracted with 3 × 20 mL CH_2Cl_2. The organic layers were combined and dried with $MgSO_4$, before evaporation to form a yellow oil, (^{P}**L**). This yellow oil was used in the next step without any further purification.

The second step involves the reaction between 2.1 equiv. of 2-picolyl chloride hydrochloride and ^{P}**L** (1 equiv.) in 20 mL water. Thereafter, 10 mL of an 8 M NaOH solution was added to the reaction solution. The reaction was allowed to continue for 6 days. Then, the compound was extracted with 30 mL of CH_2Cl_2. This process was repeated three times. Subsequently, the organic phase was washed with Na_2SO_4 and filtered. The solution was concentrated under vacuum to give a brown oil (**L**). The purity of the ligand was confirmed by nuclear magnetic resonance (NMR) spectroscopy.

$C_{21}H_{24}N_4S$ (M = 364.51 g mol^{-1}).

^{1}H NMR (600 MHz, $CDCl_3$) δ 8.49 (s, 3H), 7.64 (t, *J* = 7.6 Hz, 2H), 7.56 (t, *J* = 9.3 Hz, 3H), 7.15 – 7.09 (m, 4H), 3.84 (s, 4H), 2.99 (t, *J* = 7.6 Hz, 2H), 2.84 (t, *J* = 7.6 Hz, 2H), 2.82 – 2.77 (m, 2H), 2.73 – 2.68 (m, 2H).

^{13}C NMR (150 MHz, $CDCl_3$) δ 159.95, 159.25, 149.35, 148.97, 136.54, 136.40, 123.16, 122.10, 121.49, 77.27, 77.06, 76.85, 60.12, 53.82, 38.44, 31.82, 29.73.

ESI-MS (positive ion mode, CH_3CN): m/z, 365.18 $\{[L+H]\}^+$.

Synthesis of metal complex 1a

The metal complex $[Fe^{II}(L)(CH_3CN)](OTf)_2$ (**1a**) was synthesized using the following procedure. 100 mg ligand L was taken in a glass vial and dissolved in 2 mL CH_3CN inside a glovebox. To this solution was added 1.2 equivalents of $[Fe^{II}(CF_3SO_3)_2].2CH_3CN$ in CH_3CN (2 mL) and stirred overnight at room temperature. The resulting yellow solution was filtered,

layered by diethyl ether and kept at −40 °C for slow vapor diffusion. The light-yellow residue was washed with diethyl ether and dried under vacuum to obtain the desired metal complex **1** in >90% yield. Complex **1** was characterized by ESI-MS, UV-vis spectroscopy.

Characterization of the complexes: UV-vis spectra and kinetic experiments were recorded on a Hewlett-Packard 8453 spectrophotometer equipped with either a constant temperature circulating water bath or a liquid nitrogen cryostat (Unisoku) with a temperature controller. NMR spectra (^{1}H, and ^{13}C) were obtained with either a Bruker Avance III HD 400 MHz or 600 MHz NMR spectrometers using tetramethylsilane as the internal standard. High resolution electrospray ionization-mass spectrometry (ESI-MS) spectra of the iron(II) and iron(III) complexes were recorded on an Agilent G6546A series (UHPLC-QTOF-HRMS) mass spectrometer at 298 K with a 2 kV spray voltage and 325 °C gas temperature.

X-band electron paramagnetic resonance (EPR) experiments were performed on a JEOL (JES-FA200) ESR spectrometer at 77 K in acetonitrile. Experimental conditions [frequency, 9135.99 MHz; power, 0.995 mW; field center, 336.00 mT, width, ±50.00 mT; sweep time, 30.0 s; modulation frequency, 100.00 kHz, width, 2 mT; amplitude CH1=10, CH2=2.0 and time constant, 0.03 s] were kept constant for all the samples. The cyclic voltammetry experiments were carried out at room temperature using a CH Instruments Electrochemical Analyzer CHI610F series. A three-electrode system was used with a glassy carbon electrode as the working electrode, a Pt wire as the auxiliary electrode, and an aqueous Ag/AgCl as the reference electrode. The solutions contained 1 mM of **1a** and 100 mM supporting electrolyte *tetra*-*n*-butylammonium hexafluorophosphate ($TBAPF_6$) in acetonitrile under N_2 atmosphere. All the values were reported with reference to Ag/AgCl after calibration with ferrocene. The resonance Raman spectra of **2a** were obtained at 638 nm (80 mW, Cobolt lasers, HÜBNER Photonics) excitation wavelength using a Kymera 328i motorized Czerny-Turner Spectrograph (Andor Technology) equipped with a DU 420A-BEX2-DD camera (iDus 420 CCD, Andor Technology). The CCD camera was cooled to −80 °C and the spectral slit width of the instrument was set to 120 μm.

Kinetics experiments: All the reactions were run in a 10 mm path length quartz cuvette by monitoring the UV-vis spectral changes of the reaction solutions as a function of time. The formation of the intermediate was monitored with an increase in the absorbance at its characteristic wavelength in the UV-vis spectrum as a function of time. The rate constants were determined under pseudo first-order reaction conditions with excess substrate concentrations with respect to the oxidant. The decrease in absorbance of the characteristic peak in the UV-Vis spectrum was monitored as a function of time to obtain the pseudo first-order rate constant (k_{obs}), which was further plotted against substrate concentration to get the second-order rate constant (k_2) for each particular reaction. All the reactions were run in triplicate and averaged to ensure a standard deviation of less than 10% in the obtained rate constants.

Computational Methodology: All calculations were performed using density functional theory (DFT) methods as implemented in the *Gaussian* 09 software package.[31] Geometry optimizations, analytical frequencies and constraint geometry scans were performed using the unrestricted UBP86-GD3 and UB3LYP-GD3 density functional approaches in combination with the LACV3P+ basis set with electron core potential on iron and 6-311+G* on the rest of the atoms (basis set BS3).[32] Overall these approaches give the same reactivity patterns and trends and reach the same conclusions. All geometry optimizations and analytical frequency calculations were done with an implicit solvent model included using the conductor-like polarizable continuum model with a dielectric constant representing acetonitrile.[33] Free energies contain zero-point, thermal and entropic corrections at 233 K. Our methods and

approaches were used in various previous collaborative projects and validated against experimental data and shown to reproduce experimentally determined free energies of activation to within a few kcal mol^{-1} and predict the correct product distributions.[34]

References:

[18] R. R. Fernandes, J. Lasri, M. F. C. Guedes da Silva, J. A. L. da Silva, J. J. R. Frafflsto da Silva, A. J. L. Pombeiro, *J. Mol. Catal. A: Chem*. **2011**, *351*, 100–111.

[30] W. L. Armarego, D. D. Perrin (Eds), *Purification of Laboratory Chemicals*, Pergamon Press, Oxford, 1997.

[31] *Gaussian*-09, Revision D.01, M. J. Frisch, G. W. Trucks, H. B. Schlegel, G. E. Scuseria, M. A. Robb, J. R. Cheeseman, G. Scalmani, V. Barone, B. Mennucci, G. A. Petersson, H. Nakatsuji, M. Caricato, X. Li, H. P. Hratchian, A. F. Izmaylov, J. Bloino, G. Zheng, J. L. Sonnenberg, M. Hada, M. Ehara, K. Toyota, R. Fukuda, J. Hasegawa, M. Ishida, T. Nakajima, Y. Honda, O. Kitao, H. Nakai, T. Vreven, J. A. Montgomery Jr, J. E. Peralta, F. Ogliaro, M. Bearpark, J. J. Heyd, E. Brothers, K. N. Kudin, V. N. Staroverov, T. Keith, R. Kobayashi, J. Normand, K. Raghavachari, A. Rendell, J. C. Burant, S. S. Iyengar, J. Tomasi, M. Cossi, N. Rega, J. M. Millam, M. Klene, J. E. Knox, J. B. Cross, V. Bakken, C. Adamo, J. Jaramillo, R. Gomperts, R. E. Stratmann, O. Yazyev, A. J. Austin, R. Cammi, C. Pomelli, J. W. Ochterski, R. L. Martin, K. Morokuma, V. G. Zakrzewski, G. A. Voth, P. Salvador, J. J. Dannenberg, S. Dapprich, A. D. Daniels, O. Farkas, J. B. Foresman, J. V. Ortiz, J. Cioslowski, D. J. Fox, Gaussian, Inc., Wallingford CT, USA, 2013.

[32] a) J. P. Perdew, *Phys. Rev. B* **1986**, *33*, 8822−8824; b) A. D. Becke, *J. Chem. Phys*. **1993**, *98*, 5648–5652; c) C. Lee, W. Yang, R. G. Parr, *Phys. Rev. B* **1988**, *37*, 785–789; d) S. Grimme, S. Ehrlich, L. Goerigk, *J. Comp. Chem*. **2011**, *32*, 1456−1465; e) P. J. Hay, W. R. Wadt, *J. Chem. Phys*. **1985**, *82*, 270–283; f) M. M. Francl, W. J. Pietro, W. J. Hehre, J. S. Binkley, M. S. Gordon, D. J. DeFrees, J. A. Pople, *J. Chem. Phys*. **1982**, *77*, 3654–3658.

[33] J. Tomasi, B. Mennucci, R. Cammi, *Chem. Rev*. **2005**, *105*, 2999–3094.

[34] a) G. Mukherjee, A. Alili, P. Barman, D. Kumar, C. V. Sastri, S. P. de Visser, *Chem. Eur. J*. **2019**, *25*, 5086–5098; b) S. P. de Visser, Y.-T. Lin, H. S. Ali, U. K. Bagha, G. Mukherjee, C. V. Sastri, *Coord. Chem. Rev*. **2021**, *439*, 213914.

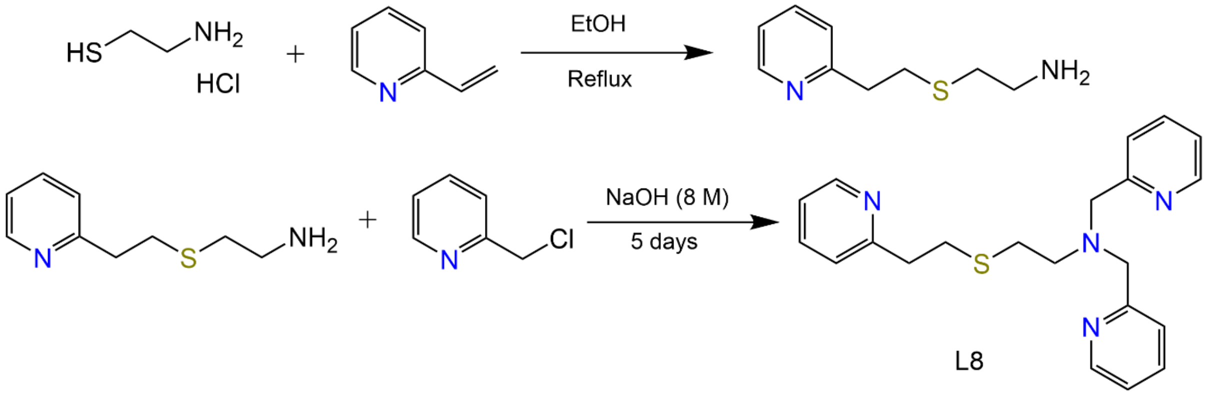

Scheme S1. Synthesis route of ligand.

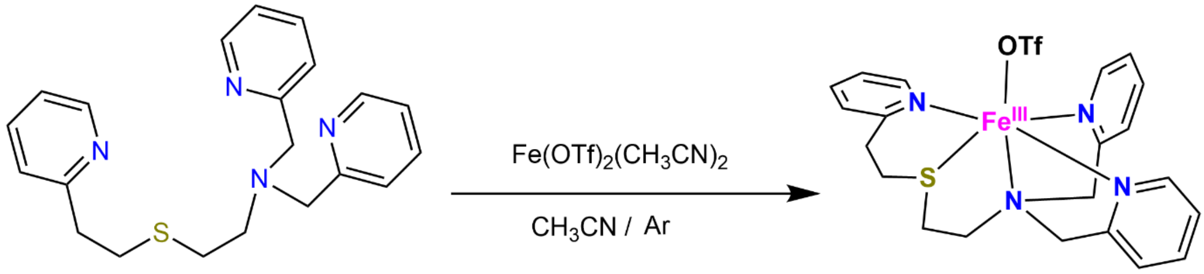

Scheme S2. Synthesis route of the iron(II) complex **1a**.

Experimental characterization

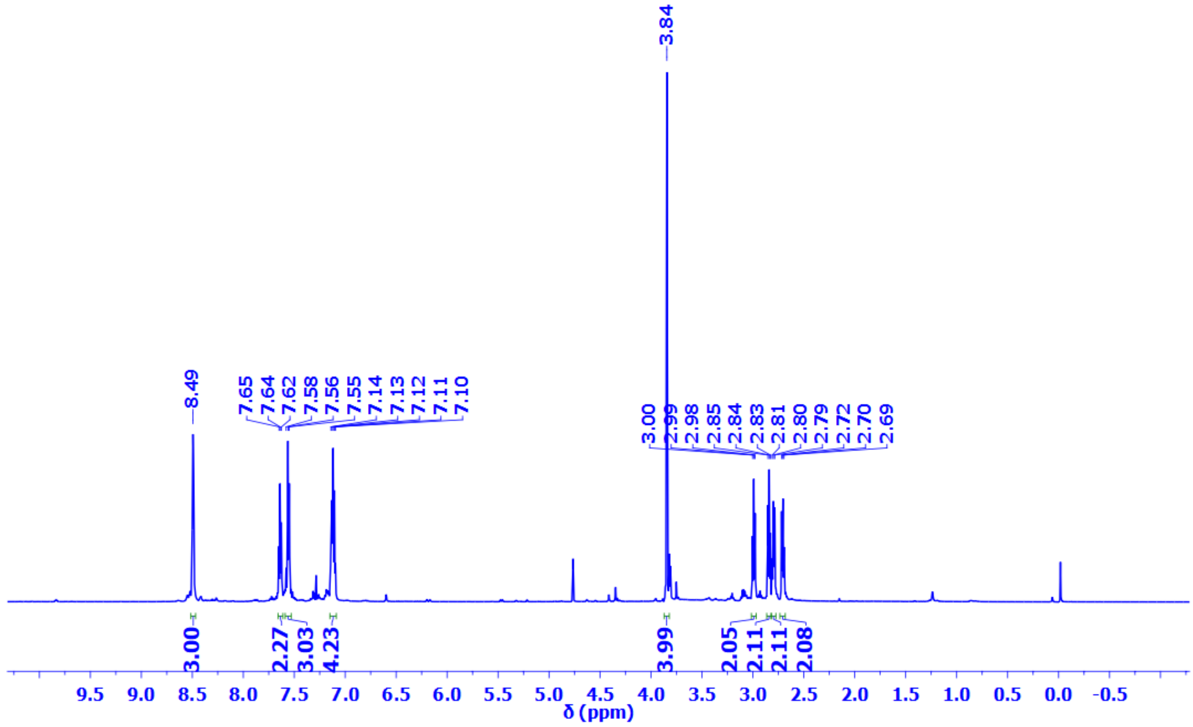

Figure S1. ^{1}H NMR spectra of L in $CDCl_3$ at RT.

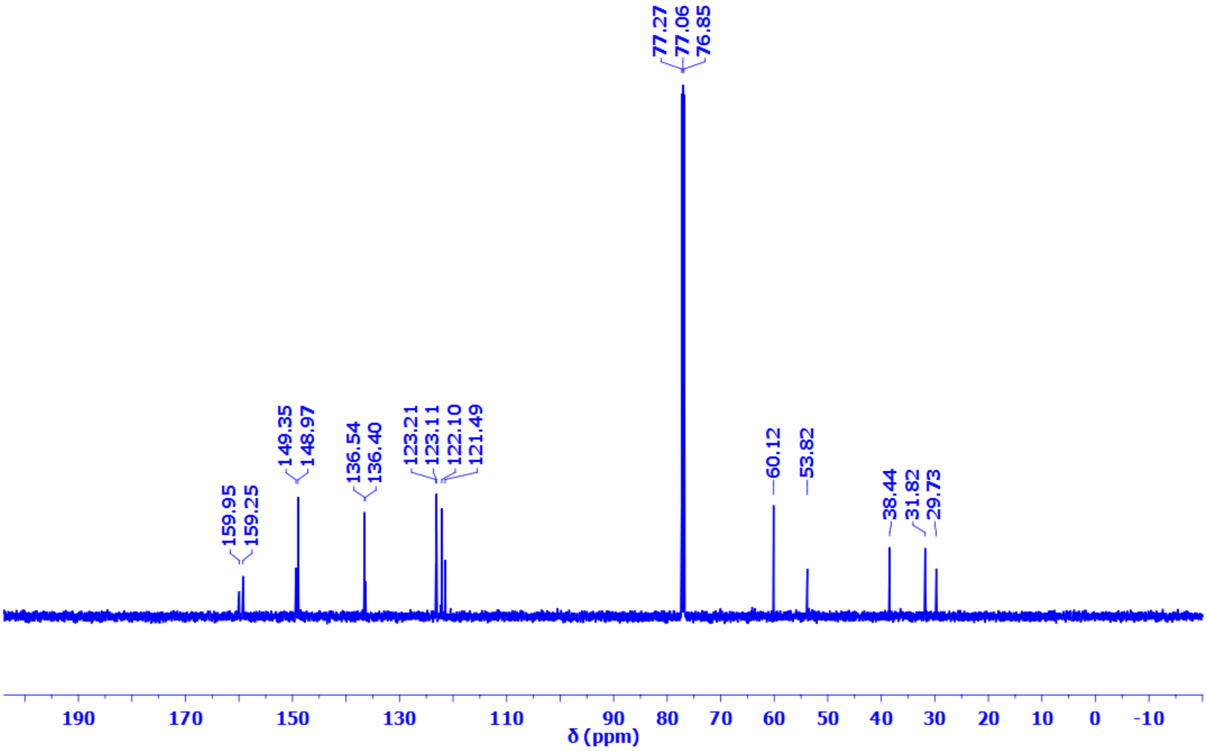

Figure S2. ^{13}C NMR spectra of L in $CDCl_3$ at RT.

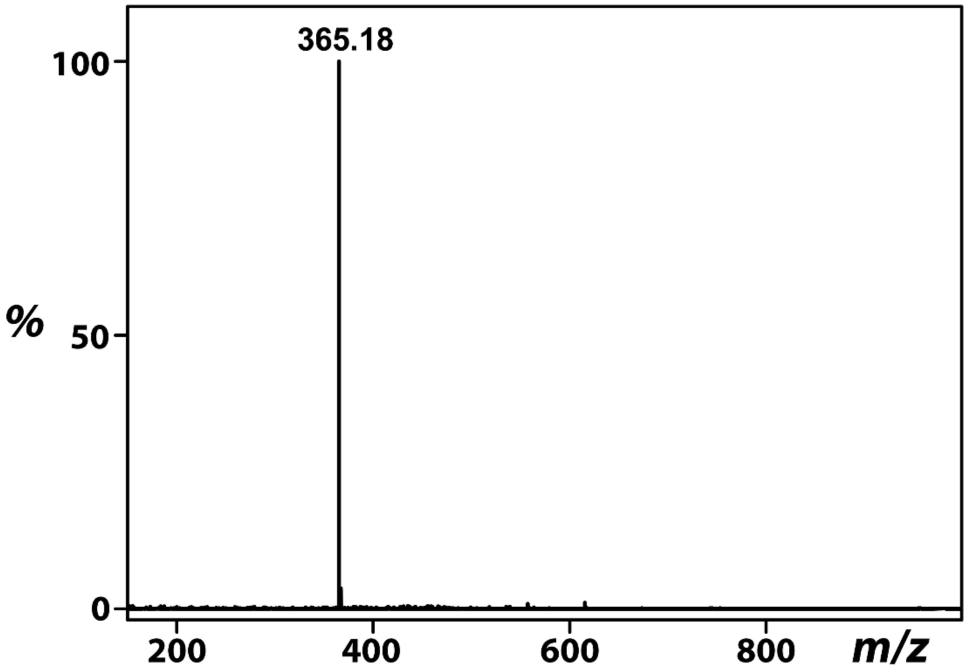

Figure S3. ESI-MS spectra of L in $CDCl_3$ at RT.

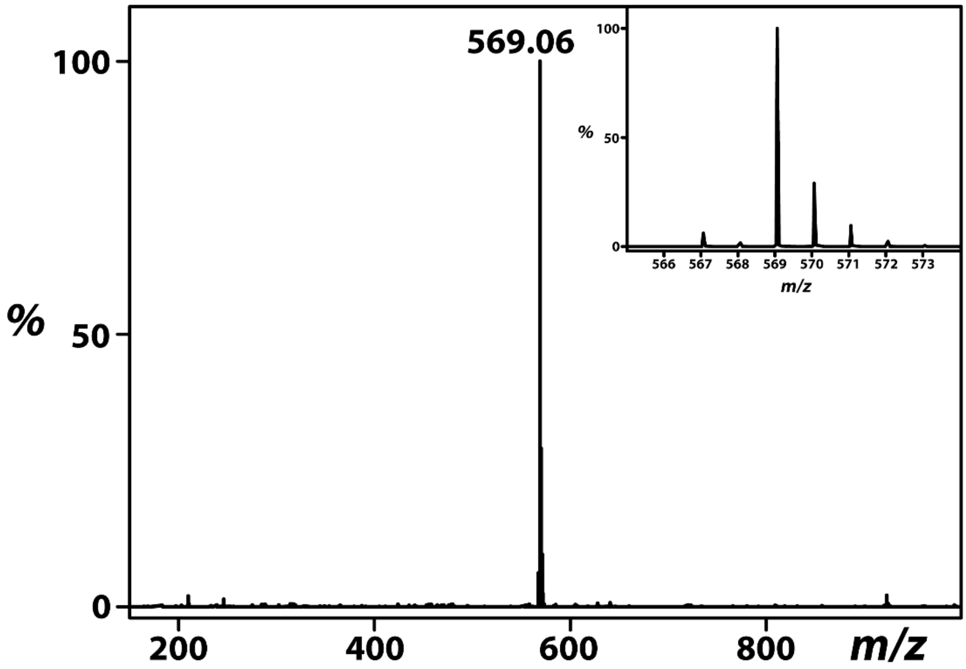

Figure S4. ESI-MS spectra of **1a** in acetonitrile at RT.

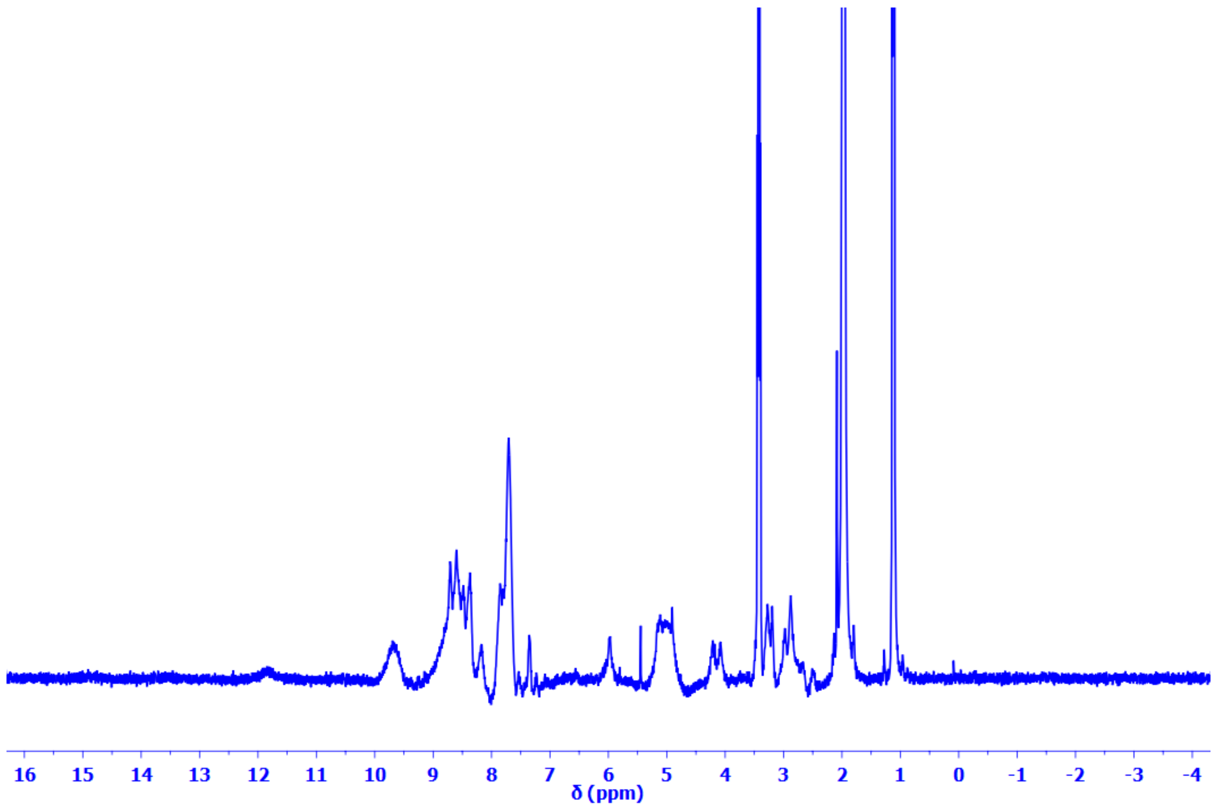

Figure S5. ^{1}H NMR spectra of **1a** in CD_3CN at RT.

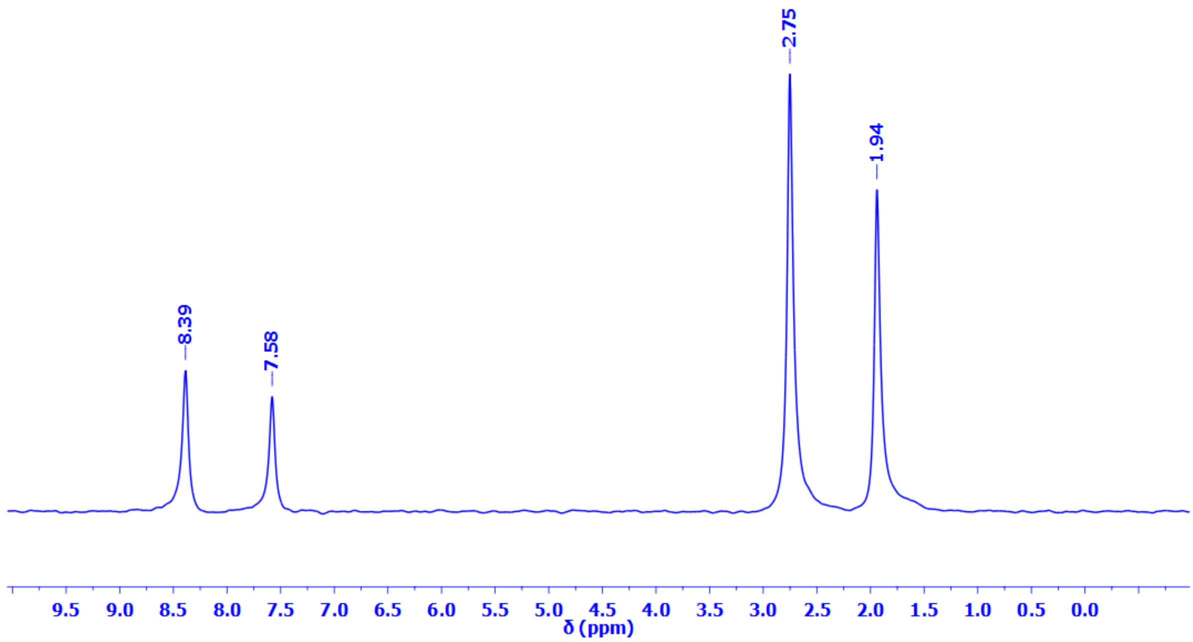

Figure S6. ^{2}H NMR spectra of **1a** in CH_3CN at RT with CD_3CN and $CDCl_3$ as markers for Evans method.

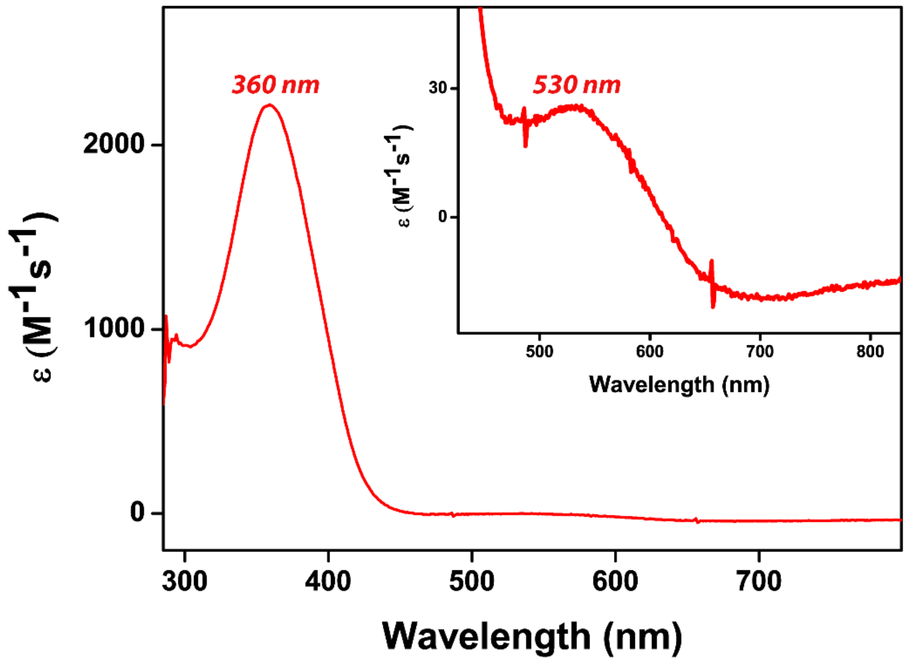

Figure S7. UV-vis spectra of **1a** (1mM) at RT.

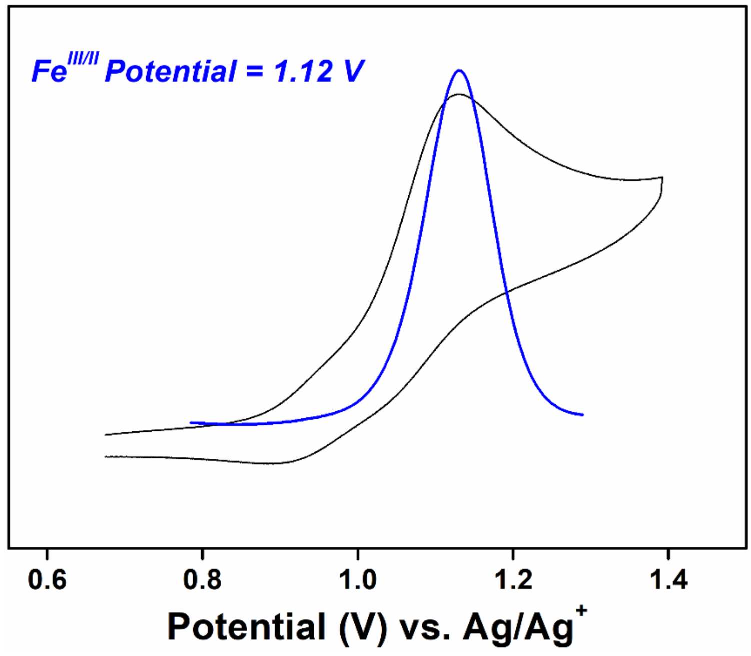

Figure S8. CV and DPV spectra of **1a** in acetonitrile at RT.

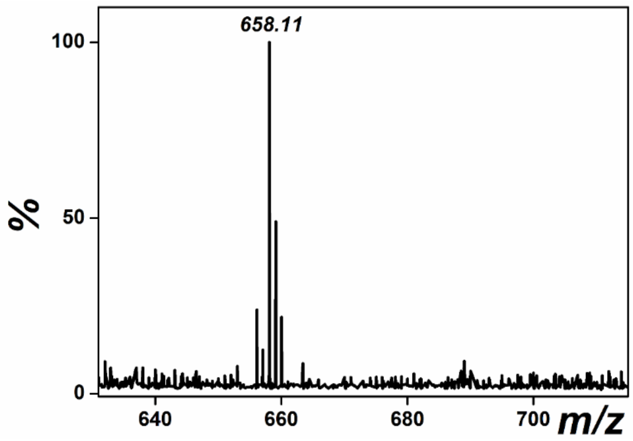

Figure S9. Electrospray ionization mass spectrum of **2a** in CH_3CN at 233 K. m/z of 658.11 corresponds to the molecular ion fragment for $[Fe^{III}(OOtBu)(OTf)]^{2+}$.

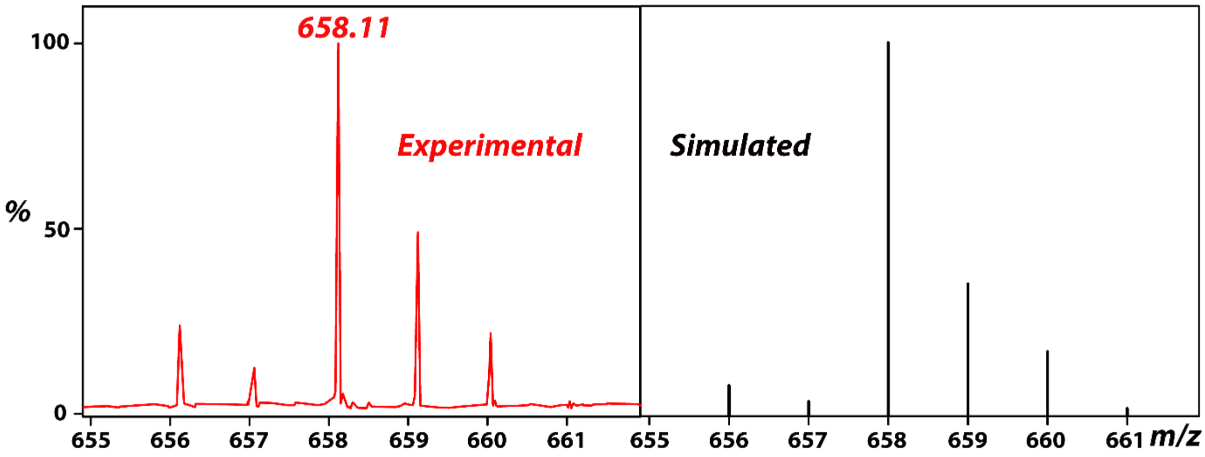

Figure S10. Electrospray ionization mass spectrum of **2a** in CH_3CN at 233 K. The plots show expanded isotopic distribution patterns of $[Fe^{III}(OO^tBu)(L)(OTf)]^+$ (Experimental in red) and (Simulated in blue).

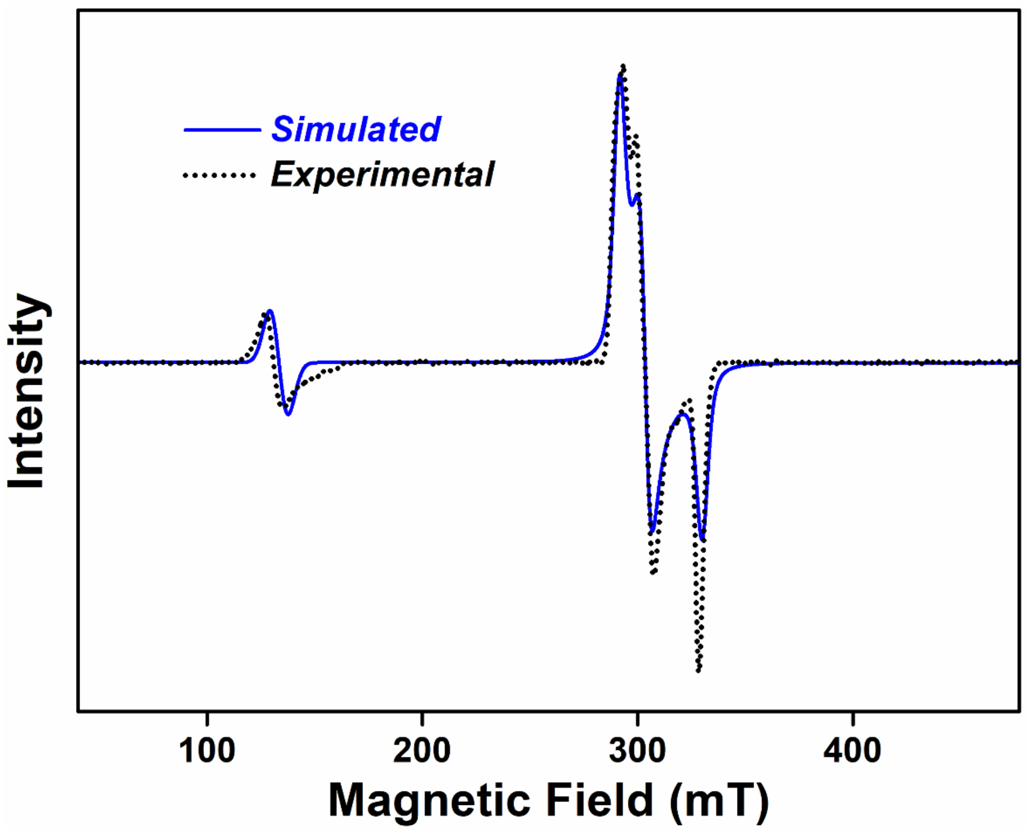

Figure S11. EPR spectrum of **2a**, experimental (black dotted lines) and simulated (blue lines) in acetonitrile at 77 K. The EPR simulation was done with the help of Easyspin.

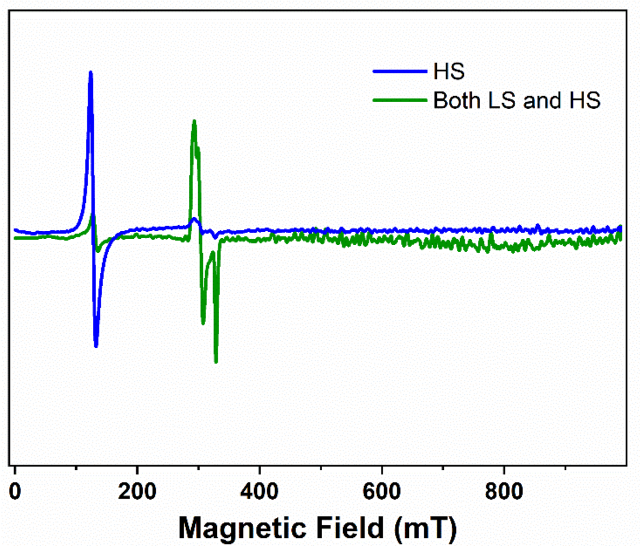

Figure S12. EPR spectrum of **2a** (Green) and EPR spectra after 2 hours (Blue) at 77 K.

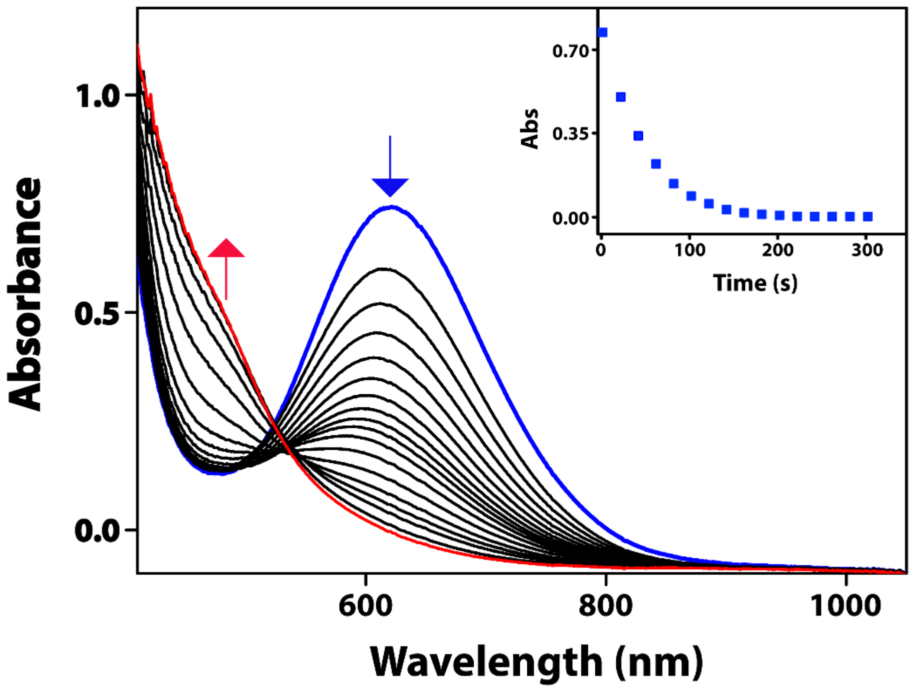

Figure S13. UV/vis spectral changes of **2a** (1mM) upon addition of 10 mM of triphenylphosphine in MeCN at 233 K. The inset shows the decay profile of the 620 nm.

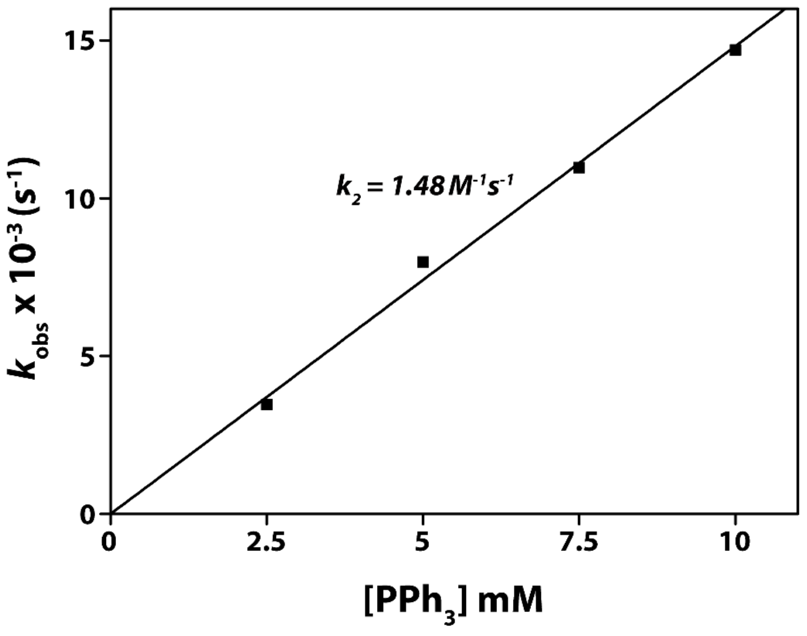

Figure S14. The second-order rate constant determined for the reaction of **2a** (1 mM) with triphenylphosphine at 233 K.

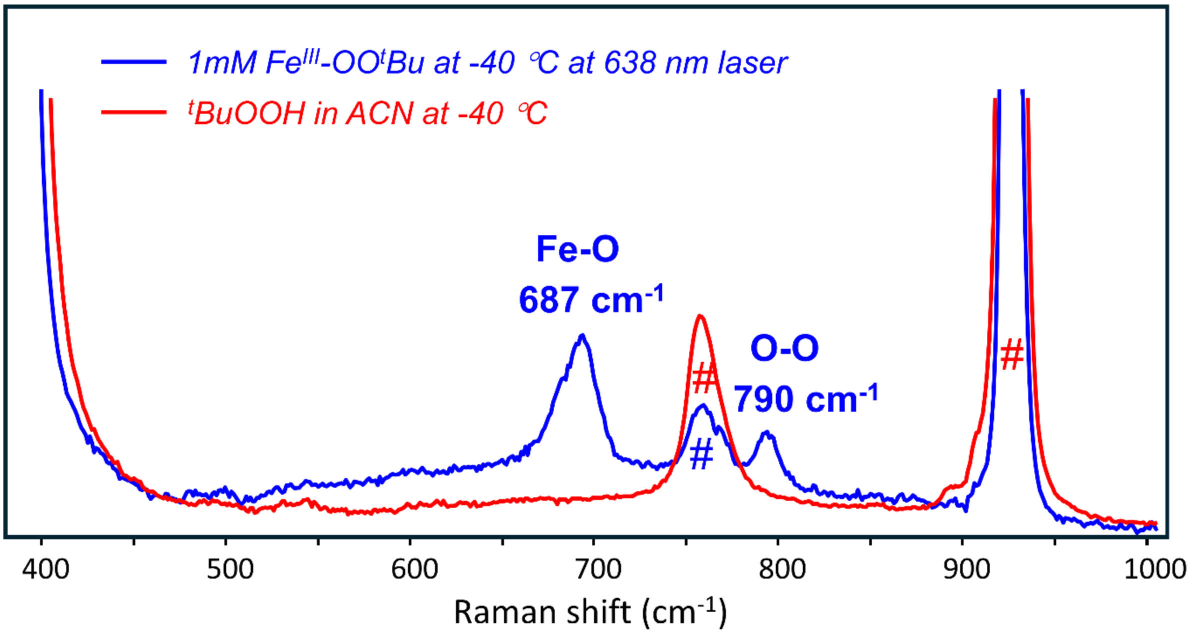

Figure S15. Resonance Raman spectra (at 638 nm excitation wavelength) nm of **2a** (1 mM) in MeCN generated by the addition of 30 equiv. of ^{t}BuOOH at 233 K (blue). The red color spectrum is for the ^{t}BuOOH in MeCN. # indicates solvent peaks.

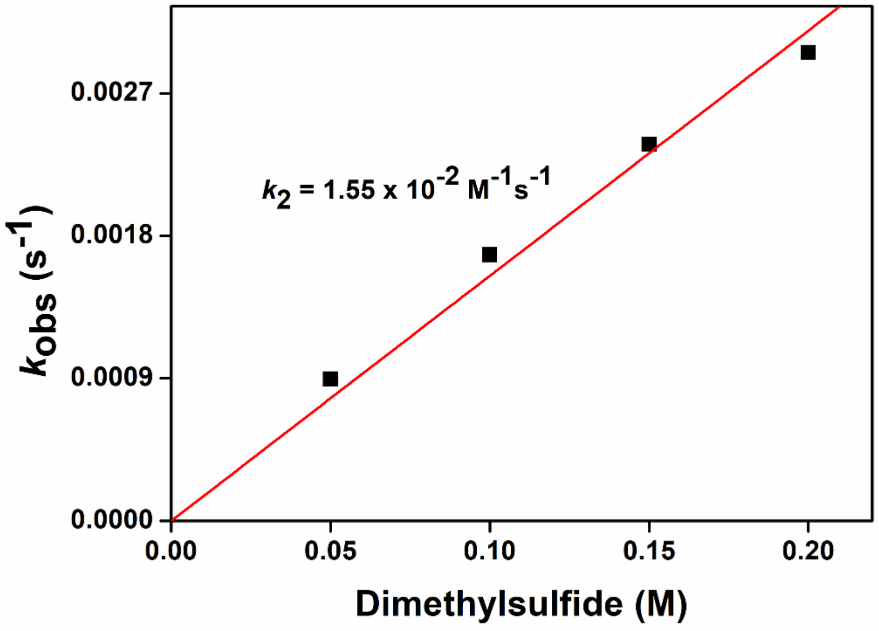

Figure S16. The second-order rate constants determined for the reaction of **2a** (1 mM) with different concentrations of dimethylsulfide at 233 K.

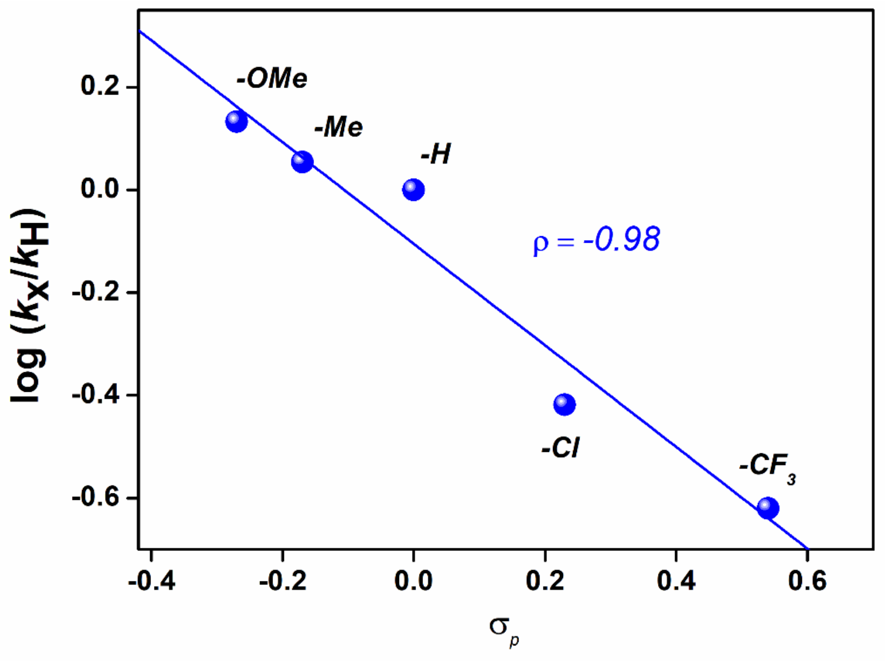

Figure S17. Hammett plot for the reaction of **2a** (1 mM) with different *para*-substituted benzaldehydes in MeCN at 233 K.

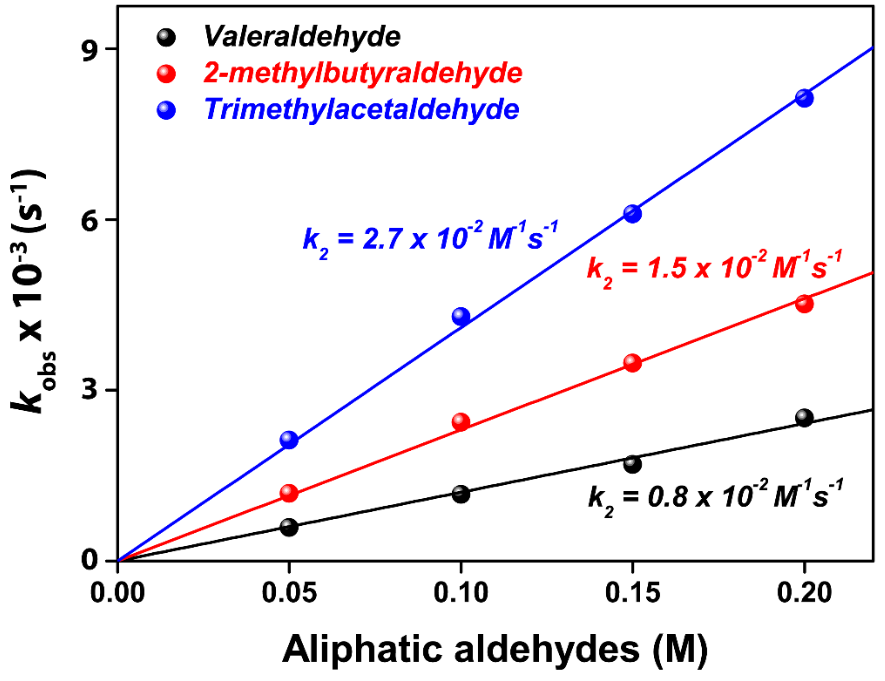

Figure S18. The second-order rate constants determined for the reaction of **2a** (1mM) with different concentrations of aliphatic aldehydes: valeraldehyde (black), 2-methylbutyraldehyde (red) & trimethylacetaldehyde (blue) at 233 K.

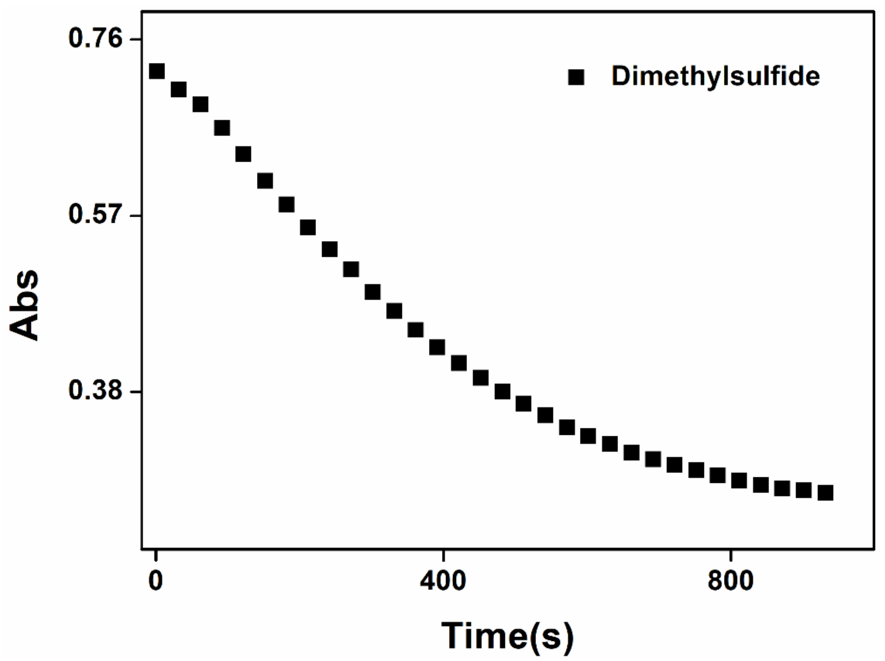

Figure S19. Time trace for the reaction of **2a (1 mM)** with 100 mM of dimethylsulfide at 233 K.

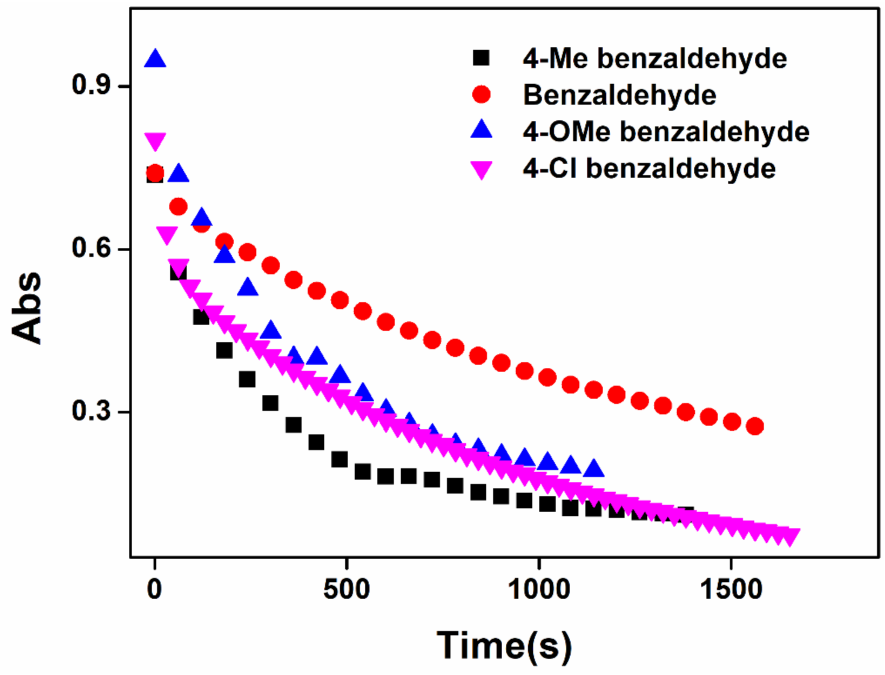

Figure S20. Time trace for the reaction of **2a (1 mM)** with 150 mM of *para*-x-benzaldehydes at 233 K.

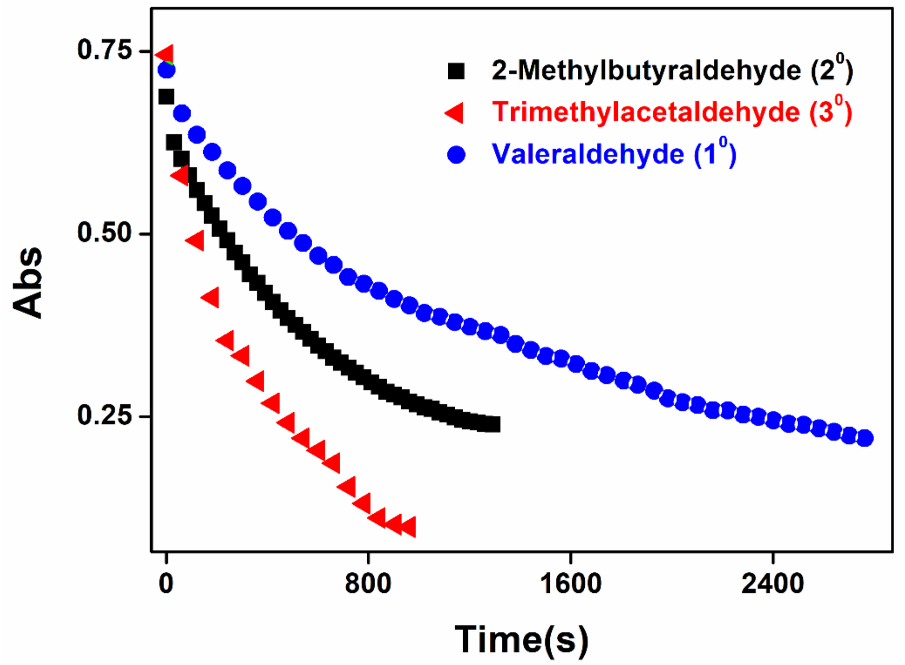

Figure S21. Time trace for the reaction of **2a (1 mM)** with 150 mM of aliphatic aldehydes at 233 K.

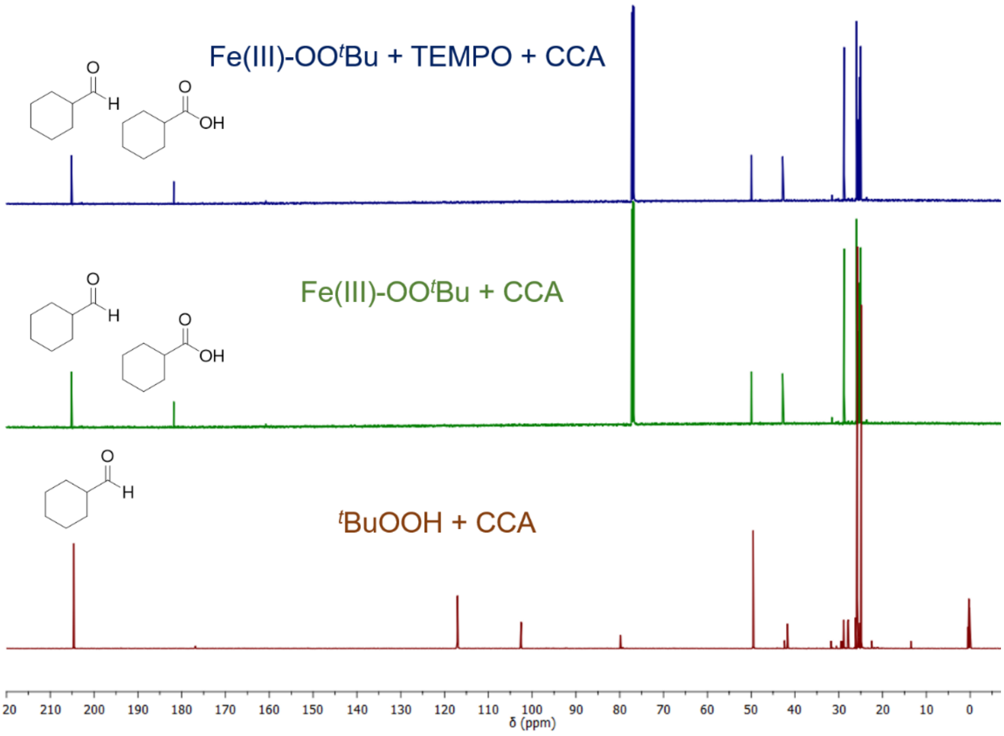

Figure S22. ^{13}C NMR spectra (600 MHz) for the oxidation of cyclohexanecarboxaldehyde with **2a** in the presence of TEMPO (*top / spectrum 3*) and in absence of TEMPO (*mid / spectrum 2*) in $CDCl_3$. Spectrum 1 shows the spectrum of the reaction between tert-butylhydroperoxide with cyclohexanecarboxaldehyde.

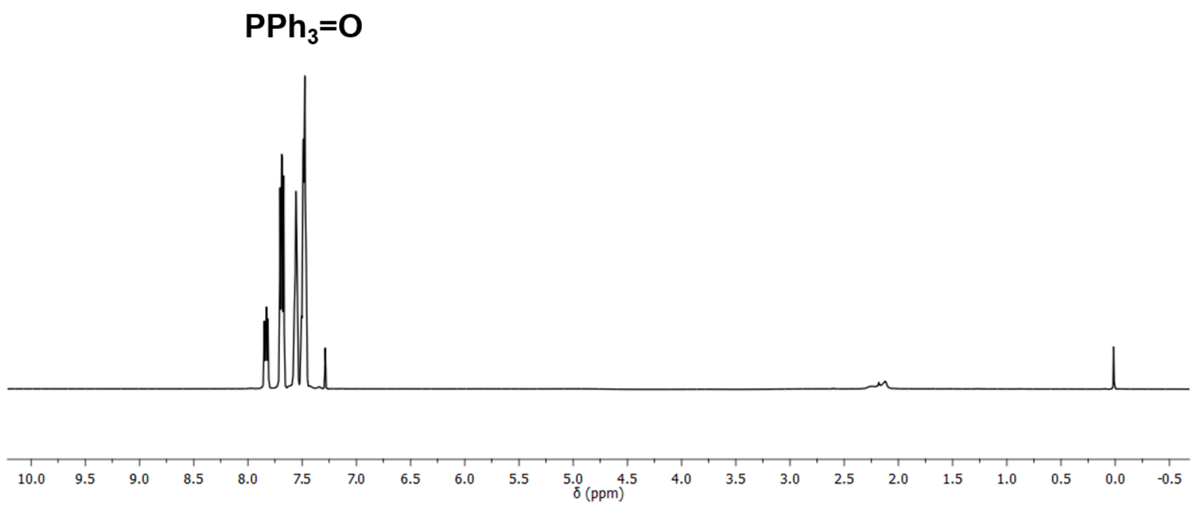

Figure S23. ^{1}H NMR spectra (600 MHz) for the oxidation of triphenylphosphine with **2a** in $CDCl_3$.

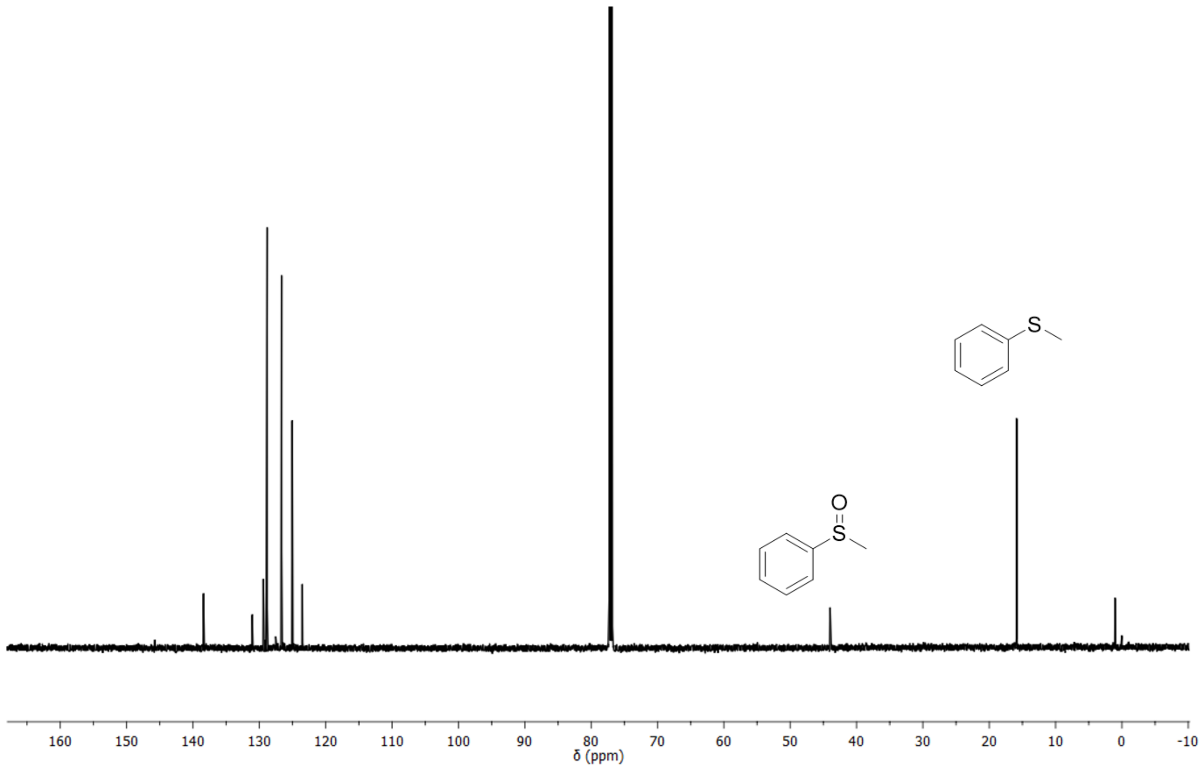

Figure S24. ^{13}C NMR spectra (600 MHz) for the oxidation of thioanisole with **2a** in $CDCl_3$.

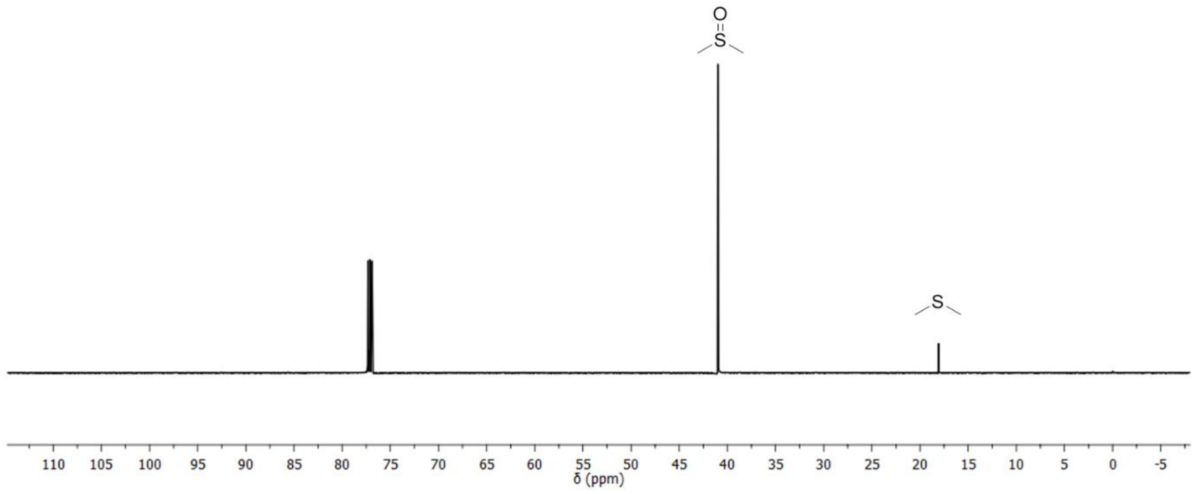

Figure S25. ^{13}C NMR spectra (600 MHz) for the oxidation of dimethylsulfide with **2a** in $CDCl_3$.

Table S1. Pseudo first-order rate constants determined for the reaction of **2a** (1 mM) with CCA with α-[D_1]-CCA and Ald-[D_1]-CCA in CH_3CN at -40°C.

Concentration (mM)	k_{obs} x 10^{-2} for CCA (s^{-1})	k_{obs} x 10^{-2} for α-[D_1]-CCA (s^{-1})	k_{obs} x 10^{-2} for Ald-[D_1]-CCA (s^{-1})
50	0.106	0.118	0.024
100	0.213	0.249	0.043
150	0.347	0.368	0.066
200	0.437	0.456	0.092

Table S2. Pseudo first-order rate constants determined for the reaction of **2a** (1 mM) with aliphatic aldehydes in CH_3CN at -40°C.

Concentration (mM)	k_{obs} x 10^{-2} for Valeraldehyde (s^{-1})	k_{obs} x 10^{-2} for 2-Methylbutyraldehyde (s^{-1})	k_{obs} x 10^{-2} for Trimethylacetaldehyde (s^{-1})
150	0.113	0.232	0.407

Table S3. Pseudo first order rate constants determined for the reaction of **2a** (1 mM) with various *para*-X benzaldehydes (150 mM) at 10 °C. [a]

para-X-benzaldehyde	σ_p[b]	Pseudo first-order rate constant (k_{obs}) (10^{-3} s^{-1})	k_x / k_H[c]	log (k_x / k_H)
-OMe	-0.27	5.23	1.358	0.133
-Me	-0.17	4.36	1.132	0.053
-H	0.0	3.85	1.0	0.0
-Cl	0.23	1.47	0.381	-0.418
-CF_3	0.54	0.924	0.240	-0.619

[a] All the reactions were followed by monitoring the UV/Vis spectral changes of the reaction solution; [b] Brown, H. C.; Okamoto, Y. *J. Am. Chem. Soc.* **1958**, *80*, 4979-4987; [c] Relative rate constant obtained by dividing the k_{obs} of *p*-X-benzaldehyde by k_{obs} of *p*-H-benzaldehyde.

Computational Results:

Table S4. Absolute (in au) and relative (in kcal mol^{-1}) energies and free energies of UBP86-GD3BJ/BS2 optimized local minima and transition states along the reaction of $[Fe^{III}(L)(OOtBu)]^{2+}$ (**2a**) in complex with DMS.

	E [BS2,au]	ZPE [au]	G [au]	DE [BS2]	DE+ZPE	DG [BS2]
^{2}**Re$_{DMS}$**	-2342.08356	0.61291	-2341.51795	0.00	0.00	0.00
^{2}**I1**	-2342.06137	0.61030	-2341.50014	13.93	12.29	11.18
^{2}**TS$_{SO}$**	-2342.05879	0.61015	-2341.49872	15.54	13.81	12.07
^{2}**PC$_{DMS}$**	-2342.08550	0.61135	-2341.52045	-1.22	-2.20	-1.57
^{4}**Re$_{DMS}$**	-2342.06137	0.61153	-2341.50133	13.93	13.06	10.43
^{4}**I1**	-2342.06151	0.61045	-2341.50230	13.84	12.29	9.82
^{4}**TS$_{SO}$**	-2342.04251	0.61002	-2341.48245	25.76	23.95	22.28
^{4}**PC$_{DMS}$**	-2342.06539	0.60835	-2341.50795	11.40	8.54	6.27
^{6}**Re$_{DMS}$**	-2342.05168	0.60959	-2341.49207	20.01	17.92	16.24
^{6}**I1**	-2342.03199	0.60815	-2341.47448	32.36	29.38	27.28
^{6}**PC$_{DMS}$**	-2342.06860	0.60888	-2341.51034	9.39	6.86	4.77
^{2}**TS$_{direct}$**	-2342.05403	0.61064	-2341.48993	18.53	17.11	17.58
^{4}**TS$_{direct}$**	-2342.02956	0.60860	-2341.46947	33.89	31.19	30.42
^{6}**TS$_{direct}$**	-2342.01481	0.60915	-2341.45334	43.14	40.78	40.55
^{2}**2a**	-1863.98451	0.53850	-1863.48768	0.00	0.00	0.00
^{2}**TS$_{OO}$**	-1863.95741	0.53526	-1863.46566	17.01	14.97	13.82
^{4}**2a**	-1863.95841	0.53685	-1863.46617	16.38	15.35	13.50
^{2}Fe---OOBu	-1863.91330	0.53698	-1863.42521	44.69	43.73	39.20
^{4}Fe---OOBu	-1863.91419	0.53477	-1863.42712	44.13	41.79	38.00
^{6}Fe---OOBu	-1863.90686	0.53370	-1863.42435	48.73	45.72	39.74
^{6}**2a**	-1863.94814	0.53481	-1863.46030	22.82	20.51	17.18
^{2}**TS$_{FeO}$**	-1863.92796	0.53678	-1863.43439	35.49	34.41	33.44
^{6}**TS$_{FeO}$**	-1863.91707	0.53460	-1863.42798	42.32	39.88	37.47

Table S5. Absolute (in au) and relative (in kcal mol^{-1}) energies and free energies of UBP86-GD3BJ/BS2 optimized local minima and transition states along the reaction of $[Fe^{III}(L)(OOtBu)]^{2+}$ (**2a**) in complex with CCA.

	E [BS2,au]	ZPE [au]	G [au]	DE [BS2]	DE+ZPE	DG [BS2]
^{3}**Re$_{CCA}$**	-1980.18607	0.59055	-1979.64250	0.00	0.00	0.00
^{3}**TS1$_{CCA,ald}$**	-1980.17387	0.58585	-1979.63232	7.66	4.71	6.39
^{3}**IM1$_{CCA,ald}$**	-1980.17783	0.59030	-1979.63268	5.17	5.02	6.16
^{3}**PR$_{CCA,ald}$**	-1980.23453	0.59231	-1979.69053	-30.40	-29.30	-30.14
^{5}**Re$_{CCA}$**	-1980.16228	0.59022	-1979.61911	14.93	14.72	14.68
^{5}**TS1$_{CCA,ald}$**	-1980.15158	0.58166	-1979.61762	21.64	16.07	15.61
^{5}**IM1$_{CCA,ald}$**	-1980.15502	0.58433	-1979.61775	19.49	15.59	15.53
^{5}**PR$_{CCA,ald}$**	-1980.23061	0.59032	-1979.68709	-27.94	-28.09	-27.98
^{3}**TS1$_{CCA,C\alpha}$**	-1980.16934	0.58438	-1979.62897	10.50	6.63	8.49
^{3}**IM1$_{CCA,C\alpha}$**	-1980.18579	0.58932	-1979.64165	0.18	-0.60	0.53
^{3}**PR$_{CCA,C\alpha}$**	-1980.19690	0.58996	-1979.65113	-6.79	-7.16	-5.42
^{5}**TS1$_{CCA,C\alpha}$**	-1980.147308	0.580224	-1979.613673	24.33	17.85	18.09
^{5}**IM1$_{CCA,C\alpha}$**	-1980.15595	0.58469	-1979.61818	18.91	15.23	15.26
^{5}**PR$_{CCA,C\alpha}$**	-1980.19687	0.58904	-1979.65424	-6.78	-7.72	-7.37

Table S6. Group spin densities and charges of UB3LYP/BS2 optimized local minima and transition states along the reaction of $[Fe^{III}(L)(OOtBu)]^{2+}$ (**2a**) in complex with DMS.

(a) Spin densities.

	Fe	O52	O-tBu	SMe2	Lig	Total
$^{2}\mathbf{Re_{DMS}}$	0.72	0.32	0.09	0.00	-0.13	1.00
$^{2}\mathbf{I1}$	1.47	0.85	-0.94	0.01	-0.39	1.00
$^{2}\mathbf{TS}_{SO}$	1.23	0.87	-0.87	0.00	-0.23	1.00
$^{2}\mathbf{PC_{DMS}}$	0.43	0.01	0.61	0.01	-0.06	1.00
$^{4}\mathbf{Re_{DMS}}$	2.76	0.17	0.03	0.00	0.05	3.00
$^{4}\mathbf{TS}_{SO}$	2.56	0.16	0.75	-0.34	-0.12	3.00
$^{4}\mathbf{I1}$	1.48	0.84	0.95	0.01	-0.29	3.00
$^{4}\mathbf{PC_{DMS}}$	3.52	0.08	-0.57	0.06	-0.09	3.00
$^{2}\mathbf{TS}_{direct}$	1.09	0.67	-0.73	0.13	-0.14	1.00
$^{4}\mathbf{TS}_{direct}$	2.78	0.15	0.03	-0.01	0.05	3.00
$^{2}\mathbf{2a}$	0.75	0.28	0.09		-0.12	1.00
$^{2}\mathbf{TS}_{OO}$	1.38	0.86	-0.95		-0.29	1.00
$^{4}\mathbf{2a}$	2.77	0.17	0.04		0.01	3.00
^{2}Fe---OOBu	0.00	0.63	0.37	0.00	0.00	1.00
^{4}Fe---OOBu	2.07	0.63	0.37	0.00	-0.07	3.00
$^{2}\mathbf{TS}_{FeO}$	-0.10	0.62	0.38		0.11	1.00
$^{6}\mathbf{Re}_{DMS}$	4.14	0.38	0.16	0.01	0.31	5.00
$^{6}\mathbf{I1}$	3.22	0.71	0.95	0.04	0.08	5.00
$^{6}\mathbf{PC}_{DMS}$	4.04	0.08	0.65	0.06	0.17	5.00
$^{6}\mathbf{2a}$	4.13	0.38	0.16		0.33	5.00
^{6}Fe---OOBu	3.60	0.63	0.37		0.40	5.00
$^{6}\mathbf{TS}_{FeO}$	3.70	0.55	0.33		0.43	5.00

(b) Charges.

	Fe	O52	O-tBu	SMe2	Lig	Total
$^{2}\mathbf{Re_{DMS}}$	-2.36	0.65	0.12	0.01	3.58	2.00
$^{2}\mathbf{I1}$	-3.34	0.00	0.14	0.07	5.13	2.00
$^{2}\mathbf{TS}_{SO}$	-2.37	0.20	0.08	0.06	4.04	2.00
$^{2}\mathbf{PC_{DMS}}$	-1.54	-0.05	-0.15	0.63	3.11	2.00
$^{4}\mathbf{Re_{DMS}}$	-1.93	0.53	0.27	-0.03	3.17	2.00
$^{4}\mathbf{TS}_{SO}$	-2.31	0.21	0.01	0.48	3.61	2.00
$^{4}\mathbf{I1}$	-3.23	-0.01	0.14	0.07	5.02	2.00
$^{4}\mathbf{PC_{DMS}}$	-2.11	0.09	-0.28	0.68	3.62	2.00
$^{2}\mathbf{TS}_{direct}$	-2.26	0.40	-0.16	0.07	3.95	2.00
$^{4}\mathbf{TS}_{direct}$	-2.50	0.36	0.29	-0.07	3.92	2.00
$^{2}\mathbf{2a}$	-1.81	0.32	0.33		3.16	2.00
$^{2}\mathbf{TS}_{OO}$	-1.58	-0.02	0.10		3.50	2.00
$^{4}\mathbf{2a}$	-2.44	0.44	0.35		3.64	2.00
^{2}Fe---OOBu	-0.57	-0.22	0.22	0.01	2.57	2.01
^{4}Fe---OOBu	-1.64	-0.22	0.22	-0.03	3.64	1.97
$^{2}\mathbf{TS}_{FeO}$	-1.87	-0.27	0.45		3.68	2.00
$^{6}\mathbf{Re}_{DMS}$	-2.69	0.52	0.34	-0.08	3.91	2.00
$^{6}\mathbf{I1}$	-2.64	0.04	0.12	0.08	4.39	2.00
$^{6}\mathbf{PC}_{DMS}$	-2.25	0.09	-0.09	0.83	3.41	2.00
$^{6}\mathbf{2a}$	-2.10	0.39	0.36		3.35	2.00
^{6}Fe---OOBu	-0.70	-0.22	0.22		2.70	2.00
$^{6}\mathbf{TS}_{FeO}$	-0.76	-0.27	0.38		2.65	2.00

Table S7. Group spin densities and charges of UB3LYP/BS2 optimized local minima and transition states along the reaction of $[Fe^{III}(L)(OOtBu)]^{2+}$ (**2a**) in complex with CCA.

(a) Spin densities.

	Fe	O52	CHD	Lig	Total
^{3}**Re**$_{CCA}$	1.37	0.91	0.01	-0.28	2.00
^{3}**TS1**$_{CCA,ald}$	1.01	0.57	0.54	-0.12	2.00
^{3}**IM1**$_{CCA,ald}$	0.99	0.29	0.88	-0.16	2.00
^{3}**PR**$_{CCA,ald}$	2.00	0.01	-0.02	0.01	2.00
^{5}**Re**$_{CCA}$	3.07	0.74	0.01	0.18	4.00
^{5}**TS1**$_{CCA,ald}$	3.78	0.32	-0.21	0.12	4.00
^{5}**IM1**$_{CCA,ald}$	4.19	0.25	-0.57	0.13	4.00
^{5}**PR**$_{CCA,ald}$	3.58	0.01	0.01	0.40	4.00
^{3}**TS1**$_{CCA,C\alpha}$	1.19	0.58	0.40	-0.17	2.00
^{3}**IM1**$_{CCA,C\alpha}$	1.02	0.21	0.99	-0.23	2.00
^{3}**PR**$_{CCA,C\alpha}$	2.18	-0.03	0.05	-0.21	2.00
^{5}**TS1**$_{CCA,C\alpha}$	3.72	0.37	-0.24	0.15	4.00
^{5}**IM1**$_{CCA,C\alpha}$	4.28	0.37	-0.95	0.30	4.00
^{5}**PR**$_{CCA,C\alpha}$	3.55	0.00	0.07	0.38	4.00

(b) Charges.

	Fe	O52	CHD	Lig	Total
^{3}**Re**$_{CCA}$	-3.09	0.54	-0.01	4.56	2.00
^{3}**TS1**$_{CCA,ald}$	-2.06	-0.30	0.42	3.95	2.00
^{3}**IM1**$_{CCA,ald}$	-2.27	-0.27	0.43	4.11	2.00
^{3}**PR**$_{CCA,ald}$	-1.64	-0.12	0.10	3.66	2.00
^{5}**Re**$_{CCA}$	-1.17	0.15	0.03	3.00	2.00
^{5}**TS1**$_{CCA,ald}$	-2.07	-0.07	0.59	3.55	2.00
^{5}**IM1**$_{CCA,ald}$	-1.64	-0.20	0.74	3.10	2.00
^{5}**PR**$_{CCA,ald}$	-0.62	-0.10	0.15	2.57	2.00
^{3}**TS1**$_{CCA,C\alpha}$	-2.59	0.19	0.30	4.10	2.00
^{3}**IM1**$_{CCA,C\alpha}$	-2.83	0.10	0.40	4.34	2.00
^{3}**PR**$_{CCA,C\alpha}$	-0.90	-0.12	0.10	2.91	2.00
^{5}**TS1**$_{CCA,C\alpha}$	-3.39	0.57	0.18	4.63	2.00
^{5}**IM1**$_{CCA,C\alpha}$	-1.59	-0.05	0.21	3.43	2.00
^{5}**PR**$_{CCA,C\alpha}$	-1.22	0.08	0.39	2.75	2.00

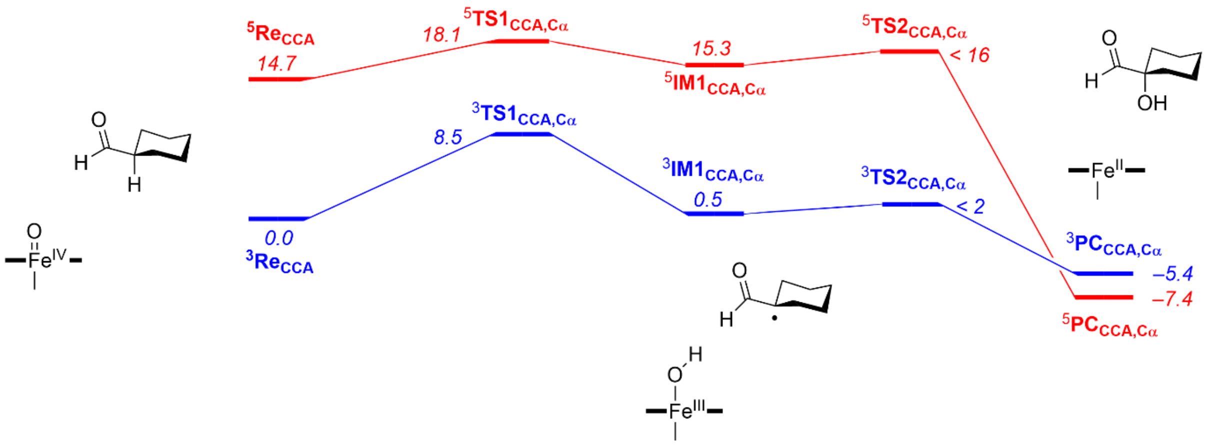

Figure S26: UBP86-GD3/BS3 calculated CCA hydroxylation at the C_{α}-H position with free energies calculated at 233 K.

Cartesian coordinates of B3LYP-GD3BJ/BS3 optimized geometries:

^{2}**Re**$_{\mathbf{DMS}}$:

26	0.133912000	-0.777934000	-0.357069000
7	-1.621973000	-0.184731000	0.661460000
6	-1.327564000	0.491672000	1.798011000
1	-0.289877000	0.720735000	1.962421000
7	1.736898000	-1.413176000	-1.357739000
6	-2.265819000	0.891420000	2.727019000
1	-1.944706000	1.422291000	3.613754000
6	-0.267225000	-2.827285000	1.563227000
1	-1.261451000	-2.450067000	1.800577000
1	-0.197031000	-3.848882000	1.942317000
6	3.772171000	-2.544664000	-2.862417000
1	4.565743000	-2.989706000	-3.450484000
7	1.173816000	-0.919030000	1.353495000
6	3.478232000	-1.188540000	-2.975008000
1	4.028991000	-0.544842000	-3.648447000
6	0.792799000	-1.940624000	2.153214000
6	2.780113000	-0.242982000	2.977273000
1	3.567519000	0.442333000	3.262769000
7	-0.089335000	-2.789495000	0.083848000
6	-2.932146000	-0.363075000	0.367831000
6	2.159514000	-0.096674000	1.745156000
1	2.416640000	0.690923000	1.051212000
6	2.008864000	-2.727472000	-1.253163000
6	1.372434000	-2.142427000	3.395157000
1	1.052781000	-2.973779000	4.010651000
6	2.373482000	-1.272486000	3.819864000
1	2.839675000	-1.409344000	4.788088000
6	-3.925535000	-0.012045000	1.288071000
1	-4.960094000	-0.201694000	1.031220000
6	1.184159000	-3.500425000	-0.271676000
1	1.772882000	-3.639610000	0.635804000
1	0.959477000	-4.498947000	-0.647510000
6	-3.602769000	0.593712000	2.488330000
1	-4.375297000	0.862549000	3.198824000
6	3.020587000	-3.325419000	-1.993404000
1	3.206431000	-4.387186000	-1.889325000
6	2.448439000	-0.658228000	-2.216493000
1	2.176628000	0.383931000	-2.268422000
6	-2.620808000	-0.462204000	-2.186774000
1	-3.164317000	-0.720640000	-3.095544000
1	-2.437452000	0.613025000	-2.191095000
6	-1.251765000	-3.382983000	-0.627873000
1	-2.147380000	-3.031381000	-0.127127000
1	-1.236658000	-4.472183000	-0.530156000
6	-1.245404000	-2.995651000	-2.099784000
1	-2.178350000	-3.274412000	-2.588132000
1	-0.437788000	-3.482942000	-2.645499000
16	-0.963940000	-1.190589000	-2.351548000
6	-3.414936000	-0.883454000	-0.965618000
1	-3.549198000	-1.966395000	-0.947900000
1	-4.424456000	-0.491951000	-1.104811000
8	0.248794000	0.978793000	-0.704865000

8	1.527649000	1.609725000	-0.659276000
6	1.339674000	3.060703000	-0.722635000
6	2.777020000	3.570103000	-0.678816000
1	2.779258000	4.661167000	-0.721149000
1	3.274521000	3.263956000	0.244125000
1	3.351395000	3.193473000	-1.527923000
6	0.650230000	3.429079000	-2.033451000
1	1.216395000	3.048101000	-2.886850000
1	-0.359330000	3.020145000	-2.070614000
1	0.581487000	4.515413000	-2.126379000
6	0.538427000	3.520939000	0.491632000
1	-0.455308000	3.074751000	0.482207000
1	1.040042000	3.237608000	1.419904000
1	0.428134000	4.607527000	0.479796000
16	-3.274606000	3.044596000	-0.760687000
6	-3.487767000	3.870203000	0.850410000
1	-2.495757000	4.095797000	1.239504000
1	-4.047694000	4.798772000	0.735101000
1	-4.001388000	3.211961000	1.550914000
6	-5.026667000	2.729223000	-1.153996000
1	-5.064865000	2.232925000	-2.123444000
1	-5.478192000	2.082099000	-0.400888000
1	-5.583806000	3.664771000	-1.213715000

^{2}**I1**$_{\mathbf{DMS}}$:

26	-0.555356000	-0.128765000	-0.254584000
7	-0.616175000	0.125230000	1.826821000
6	0.551841000	0.613204000	2.309066000
1	1.339289000	0.798063000	1.603555000
7	-0.498678000	-0.459495000	-2.213215000
6	0.777587000	0.911460000	3.637601000
1	1.744017000	1.300813000	3.928994000
6	0.704508000	-2.521506000	0.705306000
1	0.413082000	-2.281079000	1.728012000
1	0.988002000	-3.575236000	0.677837000
6	-0.479610000	-1.028060000	-4.914794000
1	-0.474142000	-1.254499000	-5.974088000
7	1.419654000	-0.441034000	-0.248875000
6	-0.620737000	0.285565000	-4.471404000
1	-0.726667000	1.108623000	-5.165798000
6	1.825861000	-1.628102000	0.250780000
6	3.666922000	0.143124000	-0.757512000
1	4.362393000	0.860246000	-1.171631000
7	-0.454844000	-2.246882000	-0.190542000
6	-1.649241000	-0.006360000	2.700223000
6	2.309681000	0.423294000	-0.756563000
1	1.900935000	1.342715000	-1.152219000
6	-0.357162000	-1.729286000	-2.628335000
6	3.169904000	-1.964650000	0.286857000
1	3.475518000	-2.923286000	0.686221000
6	4.103443000	-1.062366000	-0.217208000
1	5.158589000	-1.306663000	-0.201715000
6	-1.469116000	0.269121000	4.058440000
1	-2.311518000	0.143903000	4.726155000
6	-0.160818000	-2.768170000	-1.565774000

1	0.875864000	-3.105517000	-1.602406000
1	-0.771752000	-3.648753000	-1.767352000
6	-0.252550000	0.714549000	4.546649000
1	-0.124174000	0.927054000	5.601147000
6	-0.349134000	-2.047500000	-3.981192000
1	-0.238546000	-3.079904000	-4.288781000
6	-0.631275000	0.534689000	-3.111285000
1	-0.747505000	1.526143000	-2.697739000
6	-3.600391000	0.187635000	1.020087000
1	-4.660847000	-0.048419000	0.925912000
1	-3.495707000	1.274886000	0.996050000
6	-1.724915000	-2.809161000	0.333232000
1	-1.761659000	-2.590642000	1.395033000
1	-1.730910000	-3.898197000	0.227653000
6	-2.915444000	-2.213879000	-0.403477000
1	-3.856475000	-2.478530000	0.077149000
1	-2.972338000	-2.560078000	-1.434634000
16	-2.830565000	-0.371869000	-0.526842000
6	-3.041770000	-0.423429000	2.290271000
1	-3.129879000	-1.510793000	2.265319000
1	-3.710176000	-0.118668000	3.096926000
8	-0.524518000	1.492707000	-0.438616000
8	3.293437000	2.145089000	1.544893000
6	3.120888000	3.490093000	1.289331000
6	3.827812000	4.163802000	2.512639000
1	3.715752000	5.244289000	2.399866000
1	3.358849000	3.853522000	3.446499000
1	4.887381000	3.910158000	2.534207000
6	3.851732000	3.893869000	-0.002903000
1	4.896158000	3.579351000	0.031702000
1	3.376540000	3.431021000	-0.870913000
1	3.813746000	4.976942000	-0.137784000
6	1.638503000	3.881806000	1.264546000
1	1.105631000	3.337575000	0.482033000
1	1.168556000	3.647652000	2.220404000
1	1.520168000	4.951374000	1.076139000
16	-3.174221000	3.944730000	1.060709000
6	-1.866966000	3.563294000	2.272515000
1	-2.349481000	3.181995000	3.171574000
1	-1.198058000	2.806811000	1.867616000
1	-1.299515000	4.459748000	2.522800000
6	-2.125619000	4.438519000	-0.348954000
1	-2.789211000	4.677143000	-1.180093000
1	-1.536848000	5.321503000	-0.097086000
1	-1.468569000	3.615817000	-0.628201000

^{2}**TS**$_{SO}$:

26	-0.721838000	-0.211506000	-0.236596000
7	-0.917421000	0.173634000	1.807739000
6	0.188967000	0.782593000	2.302358000
1	0.938557000	1.087042000	1.593252000
7	-0.529311000	-0.589078000	-2.187383000
6	0.373866000	1.076221000	3.639292000
1	1.288384000	1.562406000	3.950066000
6	0.548983000	-2.499481000	0.891023000

1	0.166904000	-2.233914000	1.877437000
1	0.873570000	-3.541535000	0.927276000
6	-0.369290000	-1.264936000	-4.864828000
1	-0.311164000	-1.533177000	-5.912915000
7	1.258297000	-0.430993000	-0.092810000
6	-0.580974000	0.057753000	-4.481235000
1	-0.689088000	0.847598000	-5.213237000
6	1.669324000	-1.579849000	0.489311000
6	3.516706000	0.214795000	-0.452389000
1	4.214714000	0.943043000	-0.841994000
7	-0.545255000	-2.306190000	-0.103184000
6	-1.938943000	-0.067911000	2.671272000
6	2.152932000	0.446934000	-0.563872000
1	1.747347000	1.347639000	-0.998279000
6	-0.315100000	-1.864920000	-2.547325000
6	3.015155000	-1.864699000	0.642867000
1	3.322086000	-2.793610000	1.106391000
6	3.953710000	-0.948712000	0.169020000
1	5.012630000	-1.152910000	0.272985000
6	-1.801346000	0.208198000	4.034089000
1	-2.633077000	-0.006074000	4.692889000
6	-0.113127000	-2.847728000	-1.433842000
1	0.947793000	-3.097234000	-1.386022000
1	-0.634253000	-3.783057000	-1.640887000
6	-0.634352000	0.757573000	4.538387000
1	-0.531005000	0.960759000	5.597645000
6	-0.236270000	-2.239225000	-3.883826000
1	-0.071331000	-3.277830000	-4.142658000
6	-0.661136000	0.358823000	-3.132838000
1	-0.830394000	1.358598000	-2.760370000
6	-3.866142000	-0.078283000	0.945122000
1	-4.890169000	-0.432258000	0.824266000
1	-3.887659000	1.010386000	0.930697000
6	-1.818926000	-2.930186000	0.327783000
1	-1.940542000	-2.723477000	1.384751000
1	-1.766802000	-4.017744000	0.217708000
6	-2.982502000	-2.387641000	-0.488609000
1	-3.940445000	-2.711790000	-0.083015000
1	-2.940753000	-2.721213000	-1.524983000
16	-2.989107000	-0.542286000	-0.580774000
6	-3.281417000	-0.612603000	2.239268000
1	-3.275868000	-1.702904000	2.233202000
1	-3.992394000	-0.353789000	3.026024000
8	-0.689661000	1.499086000	-0.573191000
8	1.991568000	3.051339000	0.836483000
6	2.884046000	3.964451000	1.396400000
6	4.294639000	3.503609000	0.952507000
1	5.051851000	4.177316000	1.363323000
1	4.494288000	2.492353000	1.311190000
1	4.373594000	3.512717000	-0.136544000
6	2.624369000	5.384497000	0.861365000
1	2.661337000	5.389169000	-0.230870000
1	1.638149000	5.739167000	1.170973000
1	3.368264000	6.093210000	1.236271000
6	2.804108000	3.942550000	2.932424000

1	1.795975000	4.201475000	3.266693000
1	3.045599000	2.945348000	3.308908000
1	3.503080000	4.654527000	3.380478000
16	-1.877599000	2.950166000	0.440814000
6	-0.662869000	3.816783000	1.397371000
1	0.377671000	3.476651000	1.108195000
1	-0.738356000	4.887252000	1.212397000
1	-0.832397000	3.593175000	2.448095000
6	-1.883220000	3.905966000	-1.093365000
1	-2.534483000	3.388640000	-1.794574000
1	-2.265165000	4.906220000	-0.893261000
1	-0.868153000	3.947429000	-1.484067000

^{2}**PC$_{DMS}$**:

26	-0.844826000	-0.180939000	-0.209033000
7	-1.056119000	0.410549000	1.770997000
6	-0.053690000	1.238357000	2.146952000
1	0.761423000	1.352101000	1.454728000
7	-0.645268000	-0.657170000	-2.142775000
6	-0.035594000	1.950312000	3.331573000
1	0.803851000	2.597177000	3.548124000
6	0.616916000	-2.482733000	0.964915000
1	0.209166000	-2.190424000	1.934692000
1	1.019714000	-3.496246000	1.063862000
6	-0.508144000	-1.415570000	-4.813696000
1	-0.458736000	-1.717203000	-5.853324000
7	1.277475000	-0.375961000	-0.012740000
6	-0.819948000	-0.103496000	-4.465867000
1	-1.017888000	0.649476000	-5.218121000
6	1.705049000	-1.518990000	0.553121000
6	3.537797000	0.286768000	-0.395637000
1	4.229194000	1.013701000	-0.801560000
7	-0.474054000	-2.415186000	-0.028677000
6	-2.099448000	0.265427000	2.627698000
6	2.170060000	0.505095000	-0.483838000
1	1.760130000	1.401854000	-0.931548000
6	-0.335776000	-1.925229000	-2.473675000
6	3.058280000	-1.797307000	0.694287000
1	3.374548000	-2.727661000	1.149935000
6	3.989043000	-0.878467000	0.216063000
1	5.050239000	-1.078728000	0.305814000
6	-2.140826000	0.969508000	3.834576000
1	-2.994806000	0.835698000	4.486032000
6	-0.018583000	-2.886144000	-1.358765000
1	1.063992000	-3.028247000	-1.337807000
1	-0.441607000	-3.868800000	-1.581851000
6	-1.110552000	1.821608000	4.200412000
1	-1.148108000	2.366837000	5.136123000
6	-0.266131000	-2.335055000	-3.801921000
1	-0.023712000	-3.366126000	-4.029993000
6	-0.883272000	0.234659000	-3.124859000
1	-1.122969000	1.233321000	-2.790800000
6	-4.041330000	-0.468163000	1.078623000
1	-4.946588000	-1.075711000	1.100184000
1	-4.346400000	0.575034000	0.979992000

6	-1.695472000	-3.109559000	0.403648000
1	-1.830205000	-2.917661000	1.463500000
1	-1.588082000	-4.195925000	0.291468000
6	-2.919565000	-2.663241000	-0.394878000
1	-3.826010000	-3.109961000	0.013655000
1	-2.848739000	-2.979048000	-1.435815000
16	-3.177741000	-0.841523000	-0.482193000
6	-3.236331000	-0.687538000	2.354020000
1	-2.873491000	-1.713725000	2.398789000
1	-3.937768000	-0.601742000	3.185953000
8	-0.869397000	1.853537000	-0.730994000
8	2.215967000	3.262997000	1.422609000
6	3.393621000	3.751141000	1.941336000
6	4.161573000	4.222398000	0.659091000
1	5.118847000	4.632233000	0.987740000
1	4.342473000	3.384328000	-0.012431000
1	3.598998000	4.994728000	0.135233000
6	3.131348000	4.962406000	2.851058000
1	2.570368000	5.730352000	2.315341000
1	2.555292000	4.659057000	3.728588000
1	4.073732000	5.392644000	3.197299000
6	4.188908000	2.647552000	2.652622000
1	3.637613000	2.288370000	3.525068000
1	4.356267000	1.804373000	1.980867000
1	5.156098000	3.023497000	2.993880000
16	-1.630670000	3.005628000	0.007003000
6	-0.511296000	4.417583000	-0.135083000
1	0.362231000	4.195882000	0.476699000
1	-0.233441000	4.542060000	-1.181212000
1	-1.027348000	5.300504000	0.243052000
6	-2.900109000	3.508408000	-1.181809000
1	-3.600384000	2.679834000	-1.270648000
1	-3.410764000	4.387421000	-0.788967000
1	-2.429861000	3.723411000	-2.140449000

^{4}**Re**$_{\mathbf{DMS}}$:

26	-0.348961000	-0.438245000	-0.070638000
7	-0.303128000	-0.326842000	2.123519000
6	0.855656000	0.252045000	2.508789000
1	1.559676000	0.480960000	1.727189000
7	-0.710923000	-0.713277000	-2.105180000
6	1.162486000	0.571308000	3.817570000
1	2.116747000	1.027451000	4.046726000
6	1.397941000	-2.728202000	0.462379000
1	1.261417000	-2.602268000	1.537644000
1	1.768013000	-3.741620000	0.291681000
6	-1.545974000	-1.366173000	-4.648598000
1	-1.882565000	-1.627782000	-5.644665000
7	1.804827000	-0.509598000	-0.366061000
6	-1.691428000	-0.064068000	-4.173608000
1	-2.137915000	0.712411000	-4.780878000
6	2.362680000	-1.694698000	-0.054774000
6	3.932122000	0.340703000	-1.014564000
1	4.519780000	1.163556000	-1.400336000
7	0.073597000	-2.532449000	-0.194549000

6	-1.228122000	-0.604701000	3.077409000
6	2.563109000	0.487113000	-0.845119000
1	2.045554000	1.408949000	-1.075581000
6	-0.548740000	-1.969198000	-2.557781000
6	3.724279000	-1.914505000	-0.200838000
1	4.151022000	-2.878001000	0.047920000
6	4.520073000	-0.876739000	-0.681083000
1	5.586560000	-1.022776000	-0.803914000
6	-0.980198000	-0.279763000	4.414246000
1	-1.740283000	-0.497340000	5.153381000
6	0.172557000	-2.917710000	-1.643762000
1	1.226966000	-2.909044000	-1.925435000
1	-0.174744000	-3.942944000	-1.779705000
6	0.215494000	0.306939000	4.797962000
1	0.399498000	0.553353000	5.836941000
6	-0.967101000	-2.332364000	-3.831940000
1	-0.839886000	-3.351600000	-4.174700000
6	-1.265626000	0.224107000	-2.887849000
1	-1.368324000	1.204956000	-2.441570000
6	-3.400364000	-0.864980000	1.633648000
1	-4.382962000	-1.328820000	1.719204000
1	-3.544085000	0.215016000	1.629978000
6	-0.967031000	-3.332757000	0.508230000
1	-0.863702000	-3.121185000	1.568026000
1	-0.759283000	-4.399079000	0.370218000
6	-2.374561000	-3.027652000	0.018199000
1	-3.109174000	-3.537098000	0.641590000
1	-2.528921000	-3.380933000	-1.000982000
16	-2.789285000	-1.234559000	-0.043983000
6	-2.529011000	-1.320906000	2.793699000
1	-2.341167000	-2.394314000	2.718306000
1	-3.142454000	-1.219269000	3.690400000
8	-0.530166000	1.336314000	-0.354730000
8	0.219790000	2.181222000	0.501761000
6	-0.611499000	3.289173000	0.990728000
6	0.379197000	4.062934000	1.854588000
1	-0.110489000	4.947164000	2.267534000
1	0.737213000	3.450789000	2.684585000
1	1.237487000	4.392121000	1.265082000
6	-1.087058000	4.112897000	-0.202793000
1	-0.237310000	4.446010000	-0.803082000
1	-1.756690000	3.529898000	-0.835588000
1	-1.628726000	4.995432000	0.145147000
6	-1.781162000	2.757526000	1.809642000
1	-2.422645000	2.129337000	1.193214000
1	-1.435540000	2.185426000	2.670914000
1	-2.381139000	3.592914000	2.176611000
16	-4.125597000	1.800129000	-1.169440000
6	-5.247803000	0.568652000	-1.906607000
1	-4.668461000	-0.001740000	-2.632058000
1	-5.624934000	-0.112253000	-1.142908000
1	-6.079391000	1.057981000	-2.414633000
6	-5.278784000	2.614041000	-0.015017000
1	-4.723884000	3.398358000	0.498862000
1	-6.114806000	3.062359000	-0.552716000

1	-5.653401000	1.901182000	0.720908000

^{4}**I1**$_{\mathbf{DMS}}$:

26	0.468450000	-0.100030000	0.106409000
7	2.439781000	1.016742000	0.382686000
6	2.457285000	2.097225000	-0.427485000
1	1.637735000	2.192351000	-1.121260000
7	-1.211007000	-1.367361000	-0.039921000
6	3.444169000	3.063981000	-0.399761000
1	3.395230000	3.898991000	-1.086571000
6	2.057839000	-1.127701000	-2.112681000
1	2.946395000	-0.620464000	-1.735024000
1	2.384933000	-1.888375000	-2.824287000
6	-3.198643000	-3.273832000	-0.044092000
1	-3.975569000	-4.028944000	-0.039451000
7	0.281864000	0.480985000	-1.895344000
6	-3.396069000	-2.054023000	0.600512000
1	-4.323309000	-1.829343000	1.111303000
6	1.128364000	-0.132033000	-2.751419000
6	-0.690478000	1.700920000	-3.688644000
1	-1.426610000	2.418915000	-4.025769000
7	1.352380000	-1.750083000	-0.953152000
6	3.423128000	0.890803000	1.304945000
6	-0.621890000	1.366857000	-2.344848000
1	-1.288681000	1.791708000	-1.604675000
6	-1.017322000	-2.530558000	-0.685174000
6	1.111198000	0.159487000	-4.105525000
1	1.792551000	-0.345724000	-4.778054000
6	0.194430000	1.097226000	-4.577648000
1	0.162907000	1.340492000	-5.632835000
6	4.439306000	1.847049000	1.391423000
1	5.209299000	1.725457000	2.142320000
6	0.262923000	-2.653661000	-1.459272000
1	0.047741000	-2.378425000	-2.493014000
1	0.620962000	-3.683640000	-1.482664000
6	4.465527000	2.935048000	0.533958000
1	5.257398000	3.671343000	0.602874000
6	-1.994454000	-3.517261000	-0.697009000
1	-1.814710000	-4.453182000	-1.211041000
6	-2.372209000	-1.121938000	0.589181000
1	-2.453049000	-0.157111000	1.070440000
6	2.247016000	-0.651752000	3.027775000
1	2.502531000	-1.335331000	3.837571000
1	1.793486000	0.236584000	3.467297000
6	2.308756000	-2.489406000	-0.084219000
1	3.172956000	-1.847569000	0.053191000
1	2.653886000	-3.390273000	-0.600460000
6	1.690177000	-2.872332000	1.250118000
1	2.440583000	-3.298969000	1.915081000
1	0.901475000	-3.615741000	1.139393000
16	0.885961000	-1.458079000	2.117813000
6	3.500661000	-0.285615000	2.247509000
1	3.905615000	-1.158259000	1.731600000
1	4.255001000	-0.041660000	2.997330000
8	-0.339700000	1.106351000	0.831336000

8	-2.921705000	2.079617000	-0.086663000
6	-2.840376000	3.255815000	0.630766000
6	-4.224462000	3.926869000	0.343566000
1	-4.241989000	4.877968000	0.880128000
1	-4.349831000	4.115973000	-0.722721000
1	-5.040028000	3.298289000	0.701137000
6	-2.708291000	2.970660000	2.134361000
1	-3.538513000	2.352182000	2.481785000
1	-1.775627000	2.440674000	2.323653000
1	-2.702046000	3.902266000	2.704836000
6	-1.709473000	4.153735000	0.108699000
1	-0.748355000	3.655302000	0.240646000
1	-1.848146000	4.368102000	-0.953310000
1	-1.682366000	5.100578000	0.652803000
16	1.756110000	2.828631000	4.151509000
6	1.191823000	3.564785000	2.583629000
1	2.064227000	3.991268000	2.091052000
1	0.751218000	2.800769000	1.944886000
1	0.465741000	4.356185000	2.771327000
6	0.157340000	2.211360000	4.777407000
1	0.348419000	1.716677000	5.729411000
1	-0.540334000	3.034183000	4.935151000
1	-0.279176000	1.492707000	4.082389000

^{4}**PC$_{DMS}$**:

26	-0.727860000	-0.126781000	-0.223629000
7	-0.896134000	0.369808000	1.990108000
6	0.022250000	1.306335000	2.296964000
1	0.891971000	1.355722000	1.659232000
7	-0.755901000	-0.623409000	-2.361953000
6	-0.111963000	2.196362000	3.350388000
1	0.659157000	2.931810000	3.535138000
6	0.817458000	-2.491063000	0.736360000
1	0.440555000	-2.280501000	1.739502000
1	1.225211000	-3.506569000	0.740740000
6	-1.137863000	-1.537942000	-4.951765000
1	-1.292754000	-1.901169000	-5.961061000
7	1.438299000	-0.300923000	-0.072778000
6	-1.402742000	-0.209593000	-4.626852000
1	-1.764377000	0.491958000	-5.368084000
6	1.887123000	-1.486891000	0.378232000
6	3.689051000	0.423289000	-0.405142000
1	4.368819000	1.195181000	-0.741441000
7	-0.316407000	-2.360507000	-0.210582000
6	-1.988647000	0.264758000	2.781201000
6	2.317620000	0.632001000	-0.461481000
1	1.896251000	1.563555000	-0.818201000
6	-0.487467000	-1.900476000	-2.672971000
6	3.244294000	-1.764640000	0.475370000
1	3.575325000	-2.731151000	0.835215000
6	4.159913000	-0.792096000	0.080974000
1	5.224182000	-0.987683000	0.138962000
6	-2.192666000	1.139839000	3.849952000
1	-3.083724000	1.036082000	4.456786000
6	0.101176000	-2.758544000	-1.580720000

1	1.186733000	-2.664775000	-1.647239000
1	-0.126877000	-3.813139000	-1.755226000
6	-1.254643000	2.123041000	4.137155000
1	-1.408538000	2.807157000	4.963602000
6	-0.676247000	-2.396457000	-3.959723000
1	-0.464409000	-3.436823000	-4.175507000
6	-1.205127000	0.203957000	-3.317661000
1	-1.406079000	1.217982000	-2.998305000
6	-3.905567000	-0.698553000	1.341114000
1	-4.710926000	-1.431666000	1.397043000
1	-4.363670000	0.291709000	1.325017000
6	-1.500946000	-3.120834000	0.239825000
1	-1.581083000	-3.004048000	1.317041000
1	-1.362097000	-4.191783000	0.048843000
6	-2.788356000	-2.667306000	-0.442519000
1	-3.640195000	-3.209594000	-0.031960000
1	-2.764978000	-2.869404000	-1.513242000
16	-3.146879000	-0.866733000	-0.316679000
6	-2.979944000	-0.846617000	2.551495000
1	-2.456470000	-1.800976000	2.515428000
1	-3.626297000	-0.901354000	3.430375000
8	-0.827682000	1.844693000	-0.729481000
8	2.215531000	3.407859000	1.402163000
6	3.345949000	3.749326000	2.109920000
6	4.342621000	4.172525000	0.976697000
1	5.279016000	4.458469000	1.460069000
1	4.529743000	3.342513000	0.297547000
1	3.950638000	5.021387000	0.417342000
6	3.081381000	4.953485000	3.027662000
1	2.684309000	5.792973000	2.454111000
1	2.357689000	4.690125000	3.802873000
1	4.003228000	5.269636000	3.520839000
6	3.908826000	2.542706000	2.875615000
1	3.206150000	2.222751000	3.648906000
1	4.078504000	1.704613000	2.197856000
1	4.852662000	2.798541000	3.362237000
16	-1.599370000	3.002327000	-0.002656000
6	-0.516459000	4.427574000	-0.235844000
1	0.382922000	4.244285000	0.350473000
1	-0.283759000	4.522833000	-1.295682000
1	-1.038090000	5.310347000	0.134720000
6	-2.922775000	3.423757000	-1.161239000
1	-3.601313000	2.573394000	-1.195856000
1	-3.444136000	4.300146000	-0.776706000
1	-2.493980000	3.621537000	-2.142647000

^{2}**TS**$_{direct,DMS}$:

26	-0.614394000	-0.861216000	-0.275290000
7	-2.064565000	-0.381530000	1.156495000
6	-1.617058000	0.547799000	2.032125000
1	-0.600082000	0.870418000	1.901534000
7	0.770443000	-1.217577000	-1.668250000
6	-2.390540000	1.102794000	3.035280000
1	-1.952839000	1.836846000	3.699539000
6	-0.248913000	-2.990418000	1.586125000

1	-1.249379000	-2.805433000	1.978243000
1	0.076929000	-3.971260000	1.942292000
6	2.660686000	-1.864509000	-3.583265000
1	3.396520000	-2.122679000	-4.335624000
7	0.780966000	-0.860433000	1.175365000
6	2.041556000	-0.616768000	-3.593969000
1	2.280246000	0.124965000	-4.345344000
6	0.704659000	-1.910663000	2.021910000
6	2.552053000	0.053822000	2.475201000
1	3.292541000	0.833019000	2.603854000
7	-0.315739000	-2.928554000	0.103547000
6	-3.368206000	-0.746176000	1.243026000
6	1.710742000	0.088396000	1.370570000
1	1.808600000	0.873422000	0.628090000
6	1.372567000	-2.418691000	-1.643130000
6	1.508052000	-1.996849000	3.147623000
1	1.423790000	-2.852652000	3.805672000
6	2.432433000	-0.985212000	3.391531000
1	3.071653000	-1.027728000	4.265272000
6	-4.199919000	-0.215758000	2.233145000
1	-5.234442000	-0.531702000	2.269321000
6	1.004770000	-3.308487000	-0.493529000
1	1.778271000	-3.214990000	0.269602000
1	0.995631000	-4.357418000	-0.793441000
6	-3.716267000	0.704511000	3.149631000
1	-4.362206000	1.111366000	3.918554000
6	2.320535000	-2.779113000	-2.592669000
1	2.781299000	-3.758422000	-2.549783000
6	1.098152000	-0.328569000	-2.620875000
1	0.585998000	0.619137000	-2.552590000
6	-3.832858000	-1.450160000	-1.198924000
1	-4.417721000	-2.162672000	-1.780855000
1	-4.208447000	-0.457809000	-1.440683000
6	-1.386735000	-3.785819000	-0.461855000
1	-2.230691000	-3.764956000	0.216989000
1	-1.053052000	-4.826474000	-0.510326000
6	-1.784429000	-3.309785000	-1.850517000
1	-2.663151000	-3.840666000	-2.215069000
1	-0.987655000	-3.458879000	-2.578023000
16	-2.145332000	-1.503889000	-1.895753000
6	-3.983527000	-1.740434000	0.289947000
1	-3.639768000	-2.746047000	0.523712000
1	-5.056321000	-1.763674000	0.489495000
8	-0.424689000	0.861209000	-0.582016000
8	2.008289000	2.471788000	-0.886142000
6	2.015192000	3.762643000	-0.395898000
6	3.547413000	4.080468000	-0.342902000
1	3.657744000	5.092213000	0.053185000
1	4.064193000	3.379659000	0.312790000
1	3.985995000	4.037924000	-1.340022000
6	1.321744000	4.749329000	-1.347977000
1	1.734678000	4.659141000	-2.354796000
1	0.248035000	4.564484000	-1.400349000
1	1.461982000	5.776324000	-1.002228000
6	1.428307000	3.818405000	1.024461000

1	0.384429000	3.501397000	1.012050000
1	1.982324000	3.167451000	1.702641000
1	1.473265000	4.839104000	1.411090000
16	-1.824050000	2.510492000	-0.698966000
6	-3.607457000	2.373909000	-0.366953000
1	-3.738697000	2.313952000	0.710993000
1	-4.102534000	3.270394000	-0.743783000
1	-4.035122000	1.495917000	-0.841760000
6	-1.841111000	2.449125000	-2.506876000
1	-0.831514000	2.660878000	-2.846444000
1	-2.145856000	1.460817000	-2.847022000
1	-2.520857000	3.209415000	-2.891523000

^{4}**TS**$_{direct,DMS}$:

26	-0.273671000	-0.649182000	-0.022832000
7	-1.887139000	-0.151456000	1.525117000
6	-1.446869000	0.771605000	2.405504000
1	-0.380808000	0.959129000	2.406664000
7	1.181719000	-1.059384000	-1.686822000
6	-2.272104000	1.466875000	3.280518000
1	-1.845701000	2.189390000	3.967475000
6	0.197979000	-3.230185000	1.538459000
1	-0.773981000	-3.104233000	2.026333000
1	0.527506000	-4.260378000	1.727579000
6	2.630602000	-1.903919000	-3.920005000
1	3.185594000	-2.239771000	-4.790333000
7	1.227208000	-1.029223000	1.550865000
6	2.263274000	-0.566456000	-3.778330000
1	2.524693000	0.175820000	-4.524424000
6	1.189125000	-2.249083000	2.125711000
6	2.996742000	-0.386259000	3.037870000
1	3.703841000	0.371648000	3.355513000
7	0.019053000	-2.965104000	0.087894000
6	-3.215158000	-0.434044000	1.508502000
6	2.126972000	-0.127619000	1.984586000
1	2.143288000	0.819228000	1.456397000
6	1.559563000	-2.348530000	-1.811140000
6	2.031835000	-2.584625000	3.184500000
1	1.983163000	-3.577043000	3.619915000
6	2.938081000	-1.635830000	3.656386000
1	3.601999000	-1.876340000	4.480565000
6	-4.106640000	0.249705000	2.343839000
1	-5.163333000	0.009174000	2.298803000
6	1.273608000	-3.276159000	-0.652154000
1	2.112428000	-3.185933000	0.045273000
1	1.266872000	-4.318353000	-0.993093000
6	-3.640372000	1.210952000	3.236217000
1	-4.329642000	1.736736000	3.889591000
6	2.274818000	-2.805628000	-2.919576000
1	2.548795000	-3.853263000	-2.988061000
6	1.539462000	-0.188863000	-2.651748000
1	1.243885000	0.840383000	-2.492094000
6	-3.732342000	-1.350427000	-0.882273000
1	-4.346044000	-2.119226000	-1.360593000
1	-4.154835000	-0.378349000	-1.148663000

6	-1.128260000	-3.733506000	-0.456910000
1	-1.916414000	-3.738111000	0.295378000
1	-0.840840000	-4.782670000	-0.618278000
6	-1.664123000	-3.169463000	-1.772963000
1	-2.549232000	-3.731787000	-2.082996000
1	-0.928984000	-3.255261000	-2.577496000
16	-2.100440000	-1.381621000	-1.732150000
6	-3.751685000	-1.551857000	0.640321000
1	-3.246822000	-2.483758000	0.904941000
1	-4.802104000	-1.701038000	0.911938000
8	-0.642069000	1.600511000	-0.570621000
8	0.719853000	2.177314000	-0.877076000
6	0.975011000	3.481820000	-0.266715000
6	2.498054000	3.590471000	-0.450616000
1	2.839301000	4.562158000	-0.079441000
1	3.020047000	2.805305000	0.104961000
1	2.770420000	3.513043000	-1.508018000
6	0.265571000	4.593816000	-1.046835000
1	0.516278000	4.542203000	-2.110821000
1	-0.820699000	4.538313000	-0.928958000
1	0.584962000	5.571214000	-0.669842000
6	0.588222000	3.518500000	1.210262000
1	-0.488170000	3.382458000	1.340112000
1	1.113429000	2.747836000	1.781311000
1	0.862446000	4.491319000	1.631102000
16	-2.180487000	2.045790000	-1.333637000
6	-3.574048000	3.317804000	-1.464421000
1	-4.008992000	3.406338000	-0.467362000
1	-3.185123000	4.282354000	-1.798546000
1	-4.320848000	2.938234000	-2.170730000
6	-1.572524000	2.205432000	-3.040773000
1	-0.901379000	1.372674000	-3.246652000
1	-2.435347000	2.153009000	-3.708096000
1	-1.051917000	3.154242000	-3.182829000

^{2}**2a**:

26	0.013887000	0.017025000	-0.059480000
7	0.050882000	0.086369000	2.042900000
6	1.302651000	0.094223000	2.559165000
1	2.111817000	-0.011960000	1.863891000
7	-0.039234000	-0.057222000	-2.049524000
6	1.596037000	0.245475000	3.901107000
1	2.629909000	0.231470000	4.220922000
6	0.486584000	-2.698556000	0.600804000
1	0.277669000	-2.499751000	1.652252000
1	0.427932000	-3.777426000	0.445350000
6	-0.221492000	-0.305567000	-4.795933000
1	-0.295845000	-0.405053000	-5.872082000
7	1.815365000	-0.844260000	-0.139425000
6	0.056070000	0.930378000	-4.215501000
1	0.204198000	1.817009000	-4.818015000
6	1.832100000	-2.148818000	0.215355000
6	4.144741000	-0.934568000	-0.620706000
1	5.034925000	-0.424631000	-0.965014000
7	-0.524814000	-1.975088000	-0.220543000

6	-0.977689000	0.284145000	2.912493000
6	2.939449000	-0.250448000	-0.568968000
1	2.853596000	0.791224000	-0.841228000
6	-0.299853000	-1.254943000	-2.599674000
6	3.002834000	-2.888692000	0.187633000
1	2.988633000	-3.933310000	0.471575000
6	4.179725000	-2.268398000	-0.225899000
1	5.107791000	-2.826362000	-0.255123000
6	-0.737507000	0.451450000	4.278766000
1	-1.581415000	0.607563000	4.937923000
6	-0.409286000	-2.412796000	-1.653513000
1	0.484452000	-3.028788000	-1.761714000
1	-1.252164000	-3.054191000	-1.912926000
6	0.548944000	0.425018000	4.791943000
1	0.724547000	0.553225000	5.853324000
6	-0.405205000	-1.411916000	-3.976417000
1	-0.621505000	-2.388180000	-4.391942000
6	0.133667000	1.019806000	-2.836820000
1	0.331440000	1.947870000	-2.319462000
6	-2.803848000	1.164096000	1.296550000
1	-3.888049000	1.247367000	1.218669000
1	-2.397121000	2.172673000	1.366466000
6	-1.908163000	-2.178975000	0.285247000
1	-1.873316000	-2.068096000	1.363946000
1	-2.238729000	-3.200298000	0.074952000
6	-2.866834000	-1.182756000	-0.350374000
1	-3.839160000	-1.198789000	0.140702000
1	-3.036738000	-1.396599000	-1.405001000
16	-2.220133000	0.546773000	-0.309329000
6	-2.430429000	0.317396000	2.498357000
1	-2.815899000	-0.697437000	2.383445000
1	-2.989849000	0.721670000	3.343070000
8	0.453370000	1.760317000	-0.115683000
8	1.609880000	2.111800000	0.645868000
6	1.519257000	3.503941000	1.073455000
6	2.827478000	3.682349000	1.839805000
1	2.895325000	4.704599000	2.217460000
1	2.878547000	2.998916000	2.689918000
1	3.686993000	3.499539000	1.191318000
6	1.455314000	4.413399000	-0.150578000
1	2.309308000	4.231932000	-0.807385000
1	0.538102000	4.242887000	-0.715506000
1	1.475796000	5.461075000	0.158393000
6	0.309182000	3.690039000	1.984850000
1	-0.617224000	3.525477000	1.434551000
1	0.345883000	2.999909000	2.829626000
1	0.289597000	4.709216000	2.377116000

^{2}**TS**$_{OO}$:

26	-0.028362000	0.024007000	-0.109217000
7	-0.002640000	0.095570000	1.988443000
6	1.250497000	0.092799000	2.500841000
1	2.058141000	0.022625000	1.800861000
7	-0.066837000	-0.132963000	-2.096219000
6	1.542551000	0.197570000	3.847008000

1	2.576613000	0.176707000	4.165571000
6	0.460713000	-2.704013000	0.593243000
1	0.266519000	-2.488343000	1.644151000
1	0.411892000	-3.786260000	0.460179000
6	-0.208795000	-0.449139000	-4.836858000
1	-0.267350000	-0.575612000	-5.911074000
7	1.764356000	-0.844735000	-0.181666000
6	0.070995000	0.798955000	-4.283979000
1	0.236823000	1.668828000	-4.905833000
6	1.794678000	-2.147050000	0.180162000
6	4.089008000	-0.906479000	-0.673653000
1	4.972271000	-0.386587000	-1.020787000
7	-0.570939000	-2.008861000	-0.226942000
6	-1.030899000	0.279956000	2.860132000
6	2.877677000	-0.233882000	-0.614453000
1	2.775030000	0.809219000	-0.875188000
6	-0.330569000	-1.342480000	-2.618258000
6	2.973494000	-2.873097000	0.144128000
1	2.973727000	-3.916649000	0.432132000
6	4.139763000	-2.240368000	-0.280968000
1	5.073347000	-2.788592000	-0.317067000
6	-0.790298000	0.400462000	4.230944000
1	-1.634222000	0.542547000	4.893242000
6	-0.473590000	-2.477039000	-1.649173000
1	0.397602000	-3.125915000	-1.749531000
1	-1.337888000	-3.094534000	-1.895996000
6	0.495266000	0.344904000	4.743522000
1	0.670809000	0.433730000	5.808866000
6	-0.414617000	-1.532052000	-3.992566000
1	-0.632744000	-2.516854000	-4.386366000
6	0.129106000	0.922883000	-2.907781000
1	0.328913000	1.862323000	-2.412464000
6	-2.828344000	1.179698000	1.224749000
1	-3.909370000	1.293779000	1.142309000
1	-2.390122000	2.177015000	1.265126000
6	-1.943293000	-2.204964000	0.307480000
1	-1.893063000	-2.064899000	1.382645000
1	-2.275510000	-3.231916000	0.129629000
6	-2.914544000	-1.229736000	-0.340520000
1	-3.879012000	-1.230554000	0.166074000
1	-3.102205000	-1.471455000	-1.386060000
16	-2.269816000	0.500987000	-0.365184000
6	-2.481832000	0.351389000	2.445681000
1	-2.901252000	-0.652823000	2.355736000
1	-3.026009000	0.793953000	3.281156000
8	0.378989000	1.626476000	-0.196908000
8	2.032240000	2.299410000	0.790337000
6	1.708427000	3.600225000	1.191044000
6	2.988998000	4.030238000	1.954154000
1	2.851060000	5.053035000	2.313211000
1	3.166678000	3.378637000	2.811140000
1	3.860928000	4.004763000	1.298726000
6	1.480220000	4.511503000	-0.019884000
1	2.345496000	4.482612000	-0.686451000
1	0.603598000	4.179325000	-0.578000000

1	1.319015000	5.545951000	0.294335000
6	0.506195000	3.612562000	2.141820000
1	-0.386482000	3.277034000	1.614041000
1	0.678610000	2.949139000	2.990815000
1	0.322735000	4.620780000	2.520820000

[4]**2a**:

26	0.050906000	-0.037426000	-0.084316000
7	0.063300000	-0.027779000	2.257376000
6	1.335119000	-0.023113000	2.702360000
1	2.102384000	-0.204854000	1.967803000
7	-0.265662000	-0.122957000	-2.214610000
6	1.691278000	0.212871000	4.019344000
1	2.735359000	0.193936000	4.304355000
6	0.507831000	-2.807822000	0.486604000
1	0.353670000	-2.632399000	1.551815000
1	0.406849000	-3.879672000	0.305259000
6	-1.071649000	-0.400737000	-4.836120000
1	-1.395652000	-0.510520000	-5.864166000
7	1.898571000	-0.976266000	-0.226167000
6	-0.652643000	0.839681000	-4.356999000
1	-0.637596000	1.714122000	-4.994516000
6	1.859633000	-2.295490000	0.071042000
6	4.219266000	-1.146788000	-0.733299000
1	5.129948000	-0.662566000	-1.060649000
7	-0.530750000	-2.039032000	-0.262470000
6	-0.925094000	0.224899000	3.147530000
6	3.047761000	-0.412425000	-0.632074000
1	3.005743000	0.644541000	-0.852164000
6	-0.652378000	-1.323337000	-2.670736000
6	2.994703000	-3.085680000	-0.007050000
1	2.938664000	-4.140178000	0.230820000
6	4.193569000	-2.499758000	-0.408124000
1	5.093733000	-3.098472000	-0.477895000
6	-0.630605000	0.490473000	4.486924000
1	-1.441122000	0.699480000	5.173434000
6	-0.513595000	-2.466759000	-1.706086000
1	0.445939000	-2.948043000	-1.901372000
1	-1.280434000	-3.224823000	-1.868956000
6	0.682991000	0.483269000	4.935152000
1	0.910899000	0.687100000	5.974773000
6	-1.075431000	-1.499372000	-3.982647000
1	-1.396393000	-2.474609000	-4.326813000
6	-0.264520000	0.941492000	-3.030830000
1	0.048616000	1.876830000	-2.583793000
6	-2.816103000	1.050094000	1.569666000
1	-3.903796000	1.117945000	1.547606000
1	-2.424416000	2.064539000	1.644846000
6	-1.880008000	-2.237067000	0.343118000
1	-1.760122000	-2.132823000	1.416784000
1	-2.221033000	-3.258572000	0.150680000
6	-2.899670000	-1.245245000	-0.192629000
1	-3.838558000	-1.326911000	0.353885000
1	-3.125787000	-1.416356000	-1.244425000
16	-2.325473000	0.506468000	-0.106919000

6	-2.379764000	0.186579000	2.746019000
1	-2.701822000	-0.846003000	2.597226000
1	-2.960574000	0.532111000	3.603083000
8	0.509025000	1.691419000	-0.151849000
8	1.723399000	1.990640000	0.539215000
6	1.665413000	3.354126000	1.069446000
6	3.008806000	3.466090000	1.784674000
1	3.114880000	4.467257000	2.207438000
1	3.080442000	2.741173000	2.597878000
1	3.835534000	3.298862000	1.091061000
6	1.558864000	4.336805000	-0.093400000
1	2.387725000	4.197967000	-0.791441000
1	0.621481000	4.197743000	-0.633809000
1	1.590295000	5.363916000	0.277396000
6	0.499471000	3.495165000	2.042291000
1	-0.452618000	3.365942000	1.528234000
1	0.570058000	2.763219000	2.847181000
1	0.509071000	4.492773000	2.486531000

^{4}**TS**$_{OO}$:

26	0.005606000	-0.021959000	-0.120181000
7	0.020677000	-0.020485000	2.187487000
6	1.293685000	-0.045522000	2.630978000
1	2.058309000	-0.208426000	1.891573000
7	-0.315212000	-0.184036000	-2.227486000
6	1.653076000	0.142572000	3.953933000
1	2.697306000	0.099730000	4.235442000
6	0.500877000	-2.807154000	0.485542000
1	0.374925000	-2.619224000	1.552223000
1	0.409067000	-3.882689000	0.323227000
6	-1.060809000	-0.515379000	-4.860051000
1	-1.361256000	-0.646431000	-5.892667000
7	1.845674000	-0.965753000	-0.280704000
6	-0.649602000	0.734066000	-4.397571000
1	-0.616926000	1.595044000	-5.052345000
6	1.833181000	-2.283275000	0.024457000
6	4.153034000	-1.095301000	-0.841925000
1	5.048305000	-0.597087000	-1.190033000
7	-0.568333000	-2.065141000	-0.247174000
6	-0.963617000	0.228603000	3.084668000
6	2.973063000	-0.378972000	-0.713168000
1	2.904168000	0.678107000	-0.926347000
6	-0.693456000	-1.394047000	-2.666148000
6	2.978582000	-3.054225000	-0.081330000
1	2.947385000	-4.108654000	0.161287000
6	4.156398000	-2.448245000	-0.515608000
1	5.064251000	-3.032145000	-0.607663000
6	-0.661849000	0.449533000	4.430344000
1	-1.468164000	0.654916000	5.122812000
6	-0.585104000	-2.521183000	-1.680914000
1	0.350585000	-3.045773000	-1.880193000
1	-1.383514000	-3.250453000	-1.821599000
6	0.650550000	0.401807000	4.878327000
1	0.881726000	0.569533000	5.923616000
6	-1.086200000	-1.594929000	-3.984026000

1	-1.400327000	-2.577201000	-4.314230000
6	-0.291729000	0.863965000	-3.066421000
1	0.014477000	1.807389000	-2.633121000
6	-2.844046000	1.067843000	1.497008000
1	-3.928973000	1.171440000	1.485078000
1	-2.416495000	2.070290000	1.529001000
6	-1.896191000	-2.264852000	0.399631000
1	-1.754606000	-2.114394000	1.465291000
1	-2.223546000	-3.298966000	0.256861000
6	-2.943971000	-1.314384000	-0.155624000
1	-3.871383000	-1.385142000	0.411767000
1	-3.187801000	-1.533848000	-1.194666000
16	-2.395378000	0.446552000	-0.162942000
6	-2.422251000	0.230423000	2.695109000
1	-2.783269000	-0.795019000	2.587661000
1	-2.980688000	0.624274000	3.545963000
8	0.430195000	1.570648000	-0.232909000
8	2.066545000	2.103822000	0.771645000
6	1.826278000	3.418352000	1.192069000
6	3.117118000	3.731194000	1.993872000
1	3.047367000	4.754193000	2.371662000
1	3.221907000	3.050238000	2.839781000
1	4.000781000	3.652748000	1.358797000
6	1.697063000	4.366074000	-0.004638000
1	2.573573000	4.284173000	-0.651675000
1	0.811487000	4.112646000	-0.589856000
1	1.606333000	5.403666000	0.326571000
6	0.606044000	3.501195000	2.115405000
1	-0.297198000	3.243431000	1.562511000
1	0.709702000	2.813575000	2.955573000
1	0.490523000	4.513922000	2.508569000

^{2}Fe---OOBu:

26	-2.560674000	-10.039219000	2.120101000
7	-2.491858000	-9.734271000	4.193219000
6	-1.336418000	-9.150768000	4.588578000
1	-0.570426000	-9.045940000	3.839721000
7	-2.582309000	-9.932863000	0.160962000
6	-1.091447000	-8.691444000	5.870362000
1	-0.135791000	-8.237728000	6.099764000
6	-1.588128000	-12.572504000	2.675927000
1	-1.890145000	-12.458390000	3.717780000
1	-1.419502000	-13.634975000	2.484922000
6	-2.665762000	-9.976301000	-2.601416000
1	-2.702092000	-9.996190000	-3.684002000
7	-0.621544000	-10.472569000	2.054151000
6	-2.702050000	-8.763807000	-1.914421000
1	-2.765951000	-7.819086000	-2.438484000
6	-0.357531000	-11.752534000	2.393227000
6	1.705388000	-10.087296000	1.693335000
1	2.490902000	-9.403758000	1.398429000
7	-2.680343000	-12.026710000	1.819115000
6	-3.478031000	-9.846725000	5.121943000
6	0.384995000	-9.662858000	1.689439000
1	0.109515000	-8.654654000	1.403935000

6	-2.538578000	-11.104198000	-0.494218000
6	0.938462000	-12.244780000	2.421247000
1	1.119027000	-13.277600000	2.691766000
6	1.987430000	-11.395082000	2.078755000
1	3.008831000	-11.755728000	2.094435000
6	-3.287130000	-9.410020000	6.433979000
1	-4.095764000	-9.523962000	7.144543000
6	-2.384954000	-12.320216000	0.371156000
1	-1.356505000	-12.673863000	0.288653000
1	-3.017718000	-13.135800000	0.019590000
6	-2.088426000	-8.832756000	6.825611000
1	-1.943259000	-8.495998000	7.845249000
6	-2.587206000	-11.163093000	-1.881691000
1	-2.560319000	-12.122326000	-2.383799000
6	-2.666428000	-8.782960000	-0.530360000
1	-2.710387000	-7.874974000	0.058907000
6	-5.570378000	-9.716247000	3.657935000
1	-6.601067000	-10.067113000	3.603933000
1	-5.595996000	-8.639640000	3.833142000
6	-4.014496000	-12.565287000	2.193212000
1	-4.055812000	-12.615472000	3.275500000
1	-4.125744000	-13.588459000	1.822627000
6	-5.126168000	-11.687937000	1.635298000
1	-6.095358000	-11.981444000	2.036877000
1	-5.188834000	-11.758518000	0.549613000
16	-4.867785000	-9.893305000	1.983592000
6	-4.820143000	-10.434188000	4.774196000
1	-4.733311000	-11.498166000	4.559696000
1	-5.446698000	-10.376190000	5.665825000
8	2.501142000	13.255574000	-2.485532000
8	3.665691000	12.814989000	-2.071948000
6	4.506314000	13.860994000	-1.373533000
6	5.760876000	13.081128000	-1.016093000
1	6.460104000	13.737463000	-0.494331000
1	5.527932000	12.241238000	-0.358334000
1	6.255729000	12.700277000	-1.911833000
6	4.773394000	14.980505000	-2.367968000
1	5.280526000	14.601068000	-3.257581000
1	3.843474000	15.464078000	-2.669283000
1	5.414924000	15.732242000	-1.903364000
6	3.737264000	14.330414000	-0.147975000
1	2.800501000	14.809053000	-0.435753000
1	3.517807000	13.495198000	0.520588000
1	4.339278000	15.058132000	0.400326000

^{4}Fe---OOBu:

26	-2.548887000	-10.325064000	2.333260000
7	-2.508441000	-9.499613000	4.213380000
6	-1.604973000	-8.520477000	4.418043000
1	-0.981158000	-8.258513000	3.576441000
7	-2.568298000	-9.914359000	0.314360000
6	-1.464500000	-7.855856000	5.623071000
1	-0.721081000	-7.075721000	5.722744000
6	-1.707257000	-13.075274000	2.336691000
1	-2.066923000	-13.200684000	3.360180000

1	-1.510069000	-14.072425000	1.933659000
6	-2.736112000	-9.529809000	-2.419818000
1	-2.808328000	-9.383949000	-3.490996000
7	-0.635158000	-10.894916000	2.311353000
6	-2.709037000	-8.436129000	-1.556139000
1	-2.756871000	-7.420672000	-1.927617000
6	-0.451877000	-12.236885000	2.338117000
6	1.736781000	-10.584707000	2.227127000
1	2.569916000	-9.897027000	2.162443000
7	-2.751607000	-12.356906000	1.568372000
6	-3.336138000	-9.843176000	5.228509000
6	0.440272000	-10.094531000	2.229720000
1	0.248537000	-9.033349000	2.146888000
6	-2.582227000	-10.971628000	-0.511143000
6	0.818360000	-12.795604000	2.335273000
1	0.929723000	-13.872601000	2.348484000
6	1.930610000	-11.960357000	2.295610000
1	2.929817000	-12.378834000	2.293516000
6	-3.235651000	-9.219015000	6.470765000
1	-3.904946000	-9.518731000	7.267037000
6	-2.436170000	-12.337279000	0.116926000
1	-1.400502000	-12.655084000	-0.019395000
1	-3.051312000	-13.067221000	-0.414028000
6	-2.292582000	-8.221024000	6.678763000
1	-2.211480000	-7.733942000	7.643235000
6	-2.676202000	-10.813097000	-1.889986000
1	-2.700116000	-11.684962000	-2.532462000
6	-2.632739000	-8.670878000	-0.193484000
1	-2.629163000	-7.861726000	0.526809000
6	-5.460293000	-10.440584000	3.977009000
1	-6.261912000	-11.175607000	3.914038000
1	-5.903696000	-9.491949000	4.278964000
6	-4.124406000	-12.843257000	1.839043000
1	-4.198433000	-13.076895000	2.899982000
1	-4.322954000	-13.770677000	1.290380000
6	-5.159610000	-11.796456000	1.456426000
1	-6.165834000	-12.125278000	1.714334000
1	-5.150455000	-11.585324000	0.386926000
16	-4.865337000	-10.159260000	2.257541000
6	-4.400841000	-10.880111000	4.992295000
1	-3.955147000	-11.833834000	4.705442000
1	-4.917245000	-11.066629000	5.935669000
8	2.595461000	13.566542000	-2.570944000
8	3.762049000	13.124600000	-2.164605000
6	4.607625000	14.169250000	-1.470141000
6	5.863523000	13.387944000	-1.120629000
1	6.566262000	14.043146000	-0.602163000
1	5.633576000	12.547584000	-0.462409000
1	6.352946000	13.007565000	-2.019543000
6	4.870018000	15.289343000	-2.465181000
1	5.371699000	14.910133000	-3.357983000
1	3.938837000	15.773993000	-2.760799000
1	5.514916000	16.040098000	-2.003662000
6	3.846127000	14.638317000	-0.239760000
1	2.908159000	15.118082000	-0.521689000

1	3.629780000	13.802737000	0.429351000
1	4.452016000	15.364994000	0.305644000

[3]**Re**$_{CCA}$:

26	0.572093000	-0.028670000	0.222783000
7	0.240072000	-0.396525000	2.263845000
6	1.310595000	-0.976396000	2.858587000
1	2.175049000	-1.137926000	2.241138000
7	0.853359000	0.245272000	-1.721407000
6	1.350776000	-1.349279000	4.186785000
1	2.244656000	-1.815948000	4.579642000
6	-0.197564000	-2.791429000	0.223737000
1	-0.488979000	-2.734871000	1.273256000
1	-0.636125000	-3.699255000	-0.194584000
6	1.138962000	0.582795000	-4.446531000
1	1.245652000	0.714806000	-5.516363000
7	1.854164000	-1.552838000	0.079537000
6	1.779294000	1.444621000	-3.558153000
1	2.398379000	2.260653000	-3.907127000
6	1.301110000	-2.784865000	0.096656000
6	4.017180000	-2.501208000	-0.180874000
1	5.082673000	-2.350931000	-0.295544000
7	-0.701516000	-1.573500000	-0.471323000
6	-0.817884000	-0.078103000	3.052668000
6	3.180057000	-1.401263000	-0.070705000
1	3.555212000	-0.385171000	-0.081742000
6	0.240977000	-0.599875000	-2.567088000
6	2.085592000	-3.922182000	-0.009246000
1	1.621700000	-4.900103000	-0.000090000
6	3.464548000	-3.778051000	-0.140838000
1	4.096787000	-4.653585000	-0.224634000
6	-0.835919000	-0.446464000	4.401229000
1	-1.704099000	-0.191170000	4.995199000
6	-0.529000000	-1.730841000	-1.953086000
1	0.006816000	-2.660187000	-2.150456000
1	-1.504016000	-1.838231000	-2.429649000
6	0.235937000	-1.104531000	4.978469000
1	0.212656000	-1.390010000	6.023237000
6	0.359802000	-0.450936000	-3.943800000
1	-0.149658000	-1.141897000	-4.603798000
6	1.611225000	1.249611000	-2.199762000
1	2.075775000	1.879862000	-1.456850000
6	-1.730651000	1.862323000	1.620489000
1	-2.622796000	2.477015000	1.502127000
1	-0.924137000	2.502873000	1.979514000
6	-2.114109000	-1.266293000	-0.129767000
1	-2.222782000	-1.391987000	0.942368000
1	-2.785161000	-1.985118000	-0.608779000
6	-2.476357000	0.144550000	-0.569452000
1	-3.443840000	0.452091000	-0.173955000
1	-2.531748000	0.233601000	-1.653805000
16	-1.215327000	1.395369000	-0.060417000
6	-2.010380000	0.710686000	2.566063000
1	-2.777323000	0.051071000	2.155724000
1	-2.478969000	1.146598000	3.449984000

8	1.627089000	1.142829000	0.635605000
6	3.963194000	4.407398000	-0.664167000
6	3.652919000	5.907879000	-0.637186000
6	2.306155000	6.193861000	0.035245000
6	2.249717000	5.595608000	1.445063000
6	2.541090000	4.092141000	1.425000000
6	3.893888000	3.802403000	0.739462000
1	2.123852000	7.272651000	0.074373000
1	4.448031000	6.431378000	-0.090954000
1	3.661893000	6.307103000	-1.656355000
1	3.237299000	3.898466000	-1.309242000
1	4.947038000	4.225382000	-1.104704000
1	2.985143000	6.100957000	2.084091000
1	1.268249000	5.774177000	1.896089000
1	2.543299000	3.686106000	2.441593000
1	1.748277000	3.568332000	0.883099000
1	4.672455000	4.265958000	1.369392000
1	1.499706000	5.762446000	-0.572056000
6	4.211867000	2.333177000	0.780253000
1	4.104339000	1.859977000	1.775579000
8	4.582497000	1.665970000	-0.164937000

^{3}**TS1**$_{CCA\text{-}ald}$:

26	1.923628000	-0.748890000	2.813462000
7	3.248996000	0.131625000	4.200447000
6	4.548873000	-0.121797000	3.913562000
1	4.740404000	-0.750143000	3.063395000
7	0.664065000	-1.578039000	1.510784000
6	5.621128000	0.383204000	4.623762000
1	6.626991000	0.130132000	4.315107000
6	2.387638000	-2.926106000	4.608390000
1	2.863863000	-2.254670000	5.323563000
1	2.138301000	-3.851746000	5.130963000
6	-1.209516000	-2.750156000	-0.151820000
1	-1.947426000	-3.208484000	-0.799226000
7	3.202569000	-2.241574000	2.463835000
6	-0.505086000	-1.621616000	-0.565815000
1	-0.672664000	-1.176327000	-1.537873000
6	3.296606000	-3.172591000	3.436847000
6	4.793569000	-3.424126000	1.150045000
1	5.362112000	-3.494203000	0.232040000
7	1.169754000	-2.253904000	4.076151000
6	2.998637000	0.977919000	5.234203000
6	3.917806000	-2.365692000	1.335399000
1	3.780114000	-1.582534000	0.602121000
6	-0.003240000	-2.671565000	1.914926000
6	4.152637000	-4.255637000	3.313408000
1	4.207095000	-4.993858000	4.103390000
6	4.919550000	-4.377234000	2.157123000
1	5.596501000	-5.214601000	2.039015000
6	4.045275000	1.508595000	5.996280000
1	3.804364000	2.175659000	6.813484000
6	0.377520000	-3.228251000	3.254203000
1	0.980235000	-4.123515000	3.093299000
1	-0.503978000	-3.551321000	3.808929000

6	5.366310000	1.210490000	5.708792000
1	6.170693000	1.630284000	6.300305000
6	-0.956711000	-3.279979000	1.107251000
1	-1.484844000	-4.155012000	1.464993000
6	0.420081000	-1.056917000	0.294912000
1	0.993738000	-0.174066000	0.050350000
6	0.667930000	1.838065000	4.517063000
1	-0.225729000	2.299007000	4.938169000
1	1.126680000	2.563546000	3.846400000
6	0.332819000	-1.676126000	5.158133000
1	1.000910000	-1.196259000	5.865066000
1	-0.187591000	-2.471752000	5.699870000
6	-0.680128000	-0.695718000	4.588214000
1	-1.183451000	-0.138192000	5.377331000
1	-1.451443000	-1.199382000	4.006431000
16	0.082454000	0.512110000	3.419161000
6	1.608263000	1.415357000	5.629709000
1	1.133832000	0.664881000	6.263155000
1	1.722926000	2.283574000	6.280321000
8	2.463706000	0.340131000	1.562646000
6	5.503813000	4.001783000	2.739908000
6	5.186843000	5.181154000	3.667145000
6	3.992315000	5.995909000	3.158968000
6	2.757347000	5.112686000	2.951414000
6	3.059109000	3.935430000	2.018136000
6	4.258658000	3.127661000	2.562497000
1	3.761935000	6.803890000	3.859978000
1	4.967702000	4.799712000	4.672326000
1	6.070452000	5.819429000	3.762140000
1	5.828000000	4.370294000	1.760965000
1	6.325487000	3.403719000	3.143304000
1	2.419203000	4.728657000	3.922144000
1	1.930232000	5.699286000	2.541100000
1	2.184719000	3.288907000	1.906444000
1	3.304514000	4.309121000	1.018075000
1	3.958805000	2.721832000	3.534494000
1	4.257991000	6.473252000	2.207423000
6	4.479748000	1.929703000	1.673659000
1	3.476831000	1.110534000	1.686340000
8	5.401635000	1.687607000	0.958554000

^{3}**IM1**$_{CCA,ald}$:

26	0.536972000	-0.063128000	-0.696925000
7	1.173977000	0.392816000	1.261501000
6	2.430326000	-0.044009000	1.524238000
1	2.955460000	-0.514779000	0.712956000
7	-0.048939000	-0.471078000	-2.547246000
6	3.061717000	0.080974000	2.746781000
1	4.067897000	-0.299232000	2.865269000
6	-0.037812000	-2.501890000	0.658244000
1	0.114080000	-2.025845000	1.627145000
1	-0.582629000	-3.433256000	0.823349000
6	-1.045996000	-1.077377000	-5.051645000
1	-1.442098000	-1.313969000	-6.031895000
7	1.710706000	-1.674157000	-0.744458000

6	-0.135903000	-0.035469000	-4.886908000
1	0.198577000	0.559344000	-5.726926000
6	1.280013000	-2.731979000	-0.025882000
6	3.618341000	-2.903495000	-1.452113000
1	4.529970000	-2.938272000	-2.033945000
7	-0.813356000	-1.565415000	-0.204671000
6	0.533921000	1.067660000	2.252776000
6	2.842472000	-1.754661000	-1.460325000
1	3.109772000	-0.866991000	-2.017732000
6	-0.917990000	-1.484400000	-2.696838000
6	2.008294000	-3.909832000	0.024403000
1	1.641375000	-4.748006000	0.603107000
6	3.200935000	-3.991795000	-0.690468000
1	3.788579000	-4.901359000	-0.665118000
6	1.118681000	1.208125000	3.516193000
1	0.572830000	1.742197000	4.282966000
6	-1.234696000	-2.273865000	-1.461639000
1	-0.706068000	-3.226302000	-1.519450000
1	-2.296495000	-2.517261000	-1.411500000
6	2.378031000	0.699707000	3.784658000
1	2.823752000	0.813043000	4.765286000
6	-1.443938000	-1.810919000	-3.940644000
1	-2.148149000	-2.628593000	-4.030478000
6	0.338722000	0.245390000	-3.616924000
1	1.036001000	1.043752000	-3.407069000
6	-1.079158000	2.448923000	0.769397000
1	-2.004827000	3.020395000	0.839318000
1	-0.279762000	3.145227000	0.516208000
6	-1.994180000	-1.010308000	0.509700000
1	-1.670988000	-0.746312000	1.511243000
1	-2.765408000	-1.778956000	0.613749000
6	-2.556562000	0.191875000	-0.231339000
1	-3.306417000	0.712630000	0.362745000
1	-3.029575000	-0.091409000	-1.171017000
16	-1.243957000	1.398745000	-0.707707000
6	-0.815983000	1.724377000	2.076062000
1	-1.619311000	1.016340000	2.288173000
1	-0.904538000	2.473756000	2.864578000
8	1.716709000	1.131673000	-1.348137000
6	4.282195000	3.949477000	2.430440000
6	3.584337000	4.892303000	3.417001000
6	2.883140000	6.046841000	2.693211000
6	1.900196000	5.530970000	1.637306000
6	2.586905000	4.597595000	0.634060000
6	3.279387000	3.439848000	1.385415000
1	2.359601000	6.681759000	3.414473000
1	2.847375000	4.324954000	3.999142000
1	4.315874000	5.278628000	4.133387000
1	5.094625000	4.476618000	1.918940000
1	4.732522000	3.103897000	2.957555000
1	1.084575000	4.989561000	2.133290000
1	1.441011000	6.366313000	1.100515000
1	1.866951000	4.201192000	-0.087014000
1	3.335964000	5.154928000	0.060898000
1	2.494377000	2.865965000	1.885429000

1	3.635955000	6.680113000	2.207108000
6	3.903141000	2.499984000	0.380192000
1	2.349026000	1.494344000	-0.700406000
8	5.040219000	2.280430000	0.136459000

[3]**TS2**$_{CCA,ald}$:

26	0.493095000	0.059310000	-0.529714000
7	0.991389000	0.444320000	1.505721000
6	2.244608000	0.045932000	1.827198000
1	2.796473000	-0.471638000	1.065771000
7	0.024716000	-0.331817000	-2.438961000
6	2.853980000	0.285693000	3.043713000
1	3.861519000	-0.071792000	3.211158000
6	-0.008941000	-2.482966000	0.678613000
1	0.079352000	-2.042556000	1.672668000
1	-0.490199000	-3.457537000	0.784484000
6	-0.802084000	-0.952426000	-5.012096000
1	-1.133294000	-1.192377000	-6.015191000
7	1.773350000	-1.480307000	-0.583130000
6	0.067370000	0.111965000	-4.785894000
1	0.440035000	0.722040000	-5.598214000
6	1.351820000	-2.599737000	0.042454000
6	3.789207000	-2.568934000	-1.221984000
1	4.741403000	-2.522194000	-1.734084000
7	-0.814308000	-1.560587000	-0.164850000
6	0.314473000	1.169560000	2.434332000
6	2.956192000	-1.460219000	-1.215106000
1	3.222105000	-0.522825000	-1.684708000
6	-0.793826000	-1.377557000	-2.653912000
6	2.135725000	-3.742736000	0.071588000
1	1.777033000	-4.631775000	0.574675000
6	3.375782000	-3.723428000	-0.562992000
1	4.005799000	-4.604595000	-0.551888000
6	0.883582000	1.445895000	3.681533000
1	0.314428000	2.028507000	4.393909000
6	-1.151963000	-2.218731000	-1.464084000
1	-0.598390000	-3.155965000	-1.537674000
1	-2.208231000	-2.490589000	-1.482580000
6	2.152614000	0.997406000	4.006583000
1	2.586118000	1.215812000	4.974967000
6	-1.234778000	-1.706994000	-3.930487000
1	-1.901747000	-2.549453000	-4.065269000
6	0.455718000	0.393059000	-3.488292000
1	1.133348000	1.203205000	-3.269198000
6	-1.330284000	2.460647000	0.906106000
1	-2.297023000	2.962574000	0.948084000
1	-0.571710000	3.219402000	0.712880000
6	-2.033894000	-1.062427000	0.518089000
1	-1.755854000	-0.818778000	1.538367000
1	-2.790339000	-1.851845000	0.571233000
6	-2.613405000	0.143920000	-0.209937000
1	-3.408988000	0.611005000	0.369863000
1	-3.038493000	-0.130694000	-1.174668000
16	-1.354607000	1.439880000	-0.601577000
6	-1.074275000	1.716581000	2.204847000

1	-1.820397000	0.933432000	2.346533000
1	-1.272850000	2.426442000	3.009037000
8	1.766745000	1.355725000	-0.932781000
6	4.332526000	3.499287000	1.801187000
6	3.831674000	4.327743000	2.989103000
6	3.134475000	5.613267000	2.532475000
6	1.997862000	5.312080000	1.550100000
6	2.493545000	4.500683000	0.348395000
6	3.185309000	3.213901000	0.818488000
1	2.749197000	6.160720000	3.398358000
1	3.129605000	3.725117000	3.577432000
1	4.670966000	4.562634000	3.651314000
1	5.127567000	4.043399000	1.278721000
1	4.766395000	2.556900000	2.144623000
1	1.211810000	4.747130000	2.068310000
1	1.536002000	6.241176000	1.203022000
1	1.663268000	4.255791000	-0.319942000
1	3.201996000	5.099669000	-0.235321000
1	2.434164000	2.594718000	1.316160000
1	3.866715000	6.270968000	2.047172000
6	3.647512000	2.347209000	-0.331181000
1	1.439695000	2.093320000	-1.462437000
8	4.553045000	1.593207000	-0.421104000

^{3}**PR**$_{CCA,ald}$:

26	-0.139879000	-0.609050000	0.316719000
7	0.467578000	-0.084031000	2.195430000
6	1.626842000	0.594332000	2.296037000
1	2.146539000	0.799624000	1.373651000
7	-0.399595000	0.113620000	-1.608376000
6	2.136849000	1.044027000	3.501506000
1	3.070178000	1.591911000	3.516512000
6	-0.146227000	-3.427781000	-0.100080000
1	-0.350700000	-3.592315000	0.960256000
1	-0.323462000	-4.374403000	-0.618433000
6	-0.905556000	0.920713000	-4.204067000
1	-1.108411000	1.233990000	-5.221423000
7	1.494007000	-1.640907000	-0.152620000
6	-0.451541000	1.836803000	-3.257409000
1	-0.290985000	2.877130000	-3.510116000
6	1.281877000	-2.972478000	-0.279367000
6	3.799761000	-1.964178000	-0.690129000
1	4.772595000	-1.522963000	-0.864756000
7	-1.047510000	-2.351186000	-0.577243000
6	-0.238463000	-0.341327000	3.321907000
6	2.722212000	-1.146688000	-0.382283000
1	2.826909000	-0.070990000	-0.338950000
6	-0.835147000	-0.775415000	-2.513562000
6	2.315608000	-3.844430000	-0.588057000
1	2.112129000	-4.902923000	-0.691048000
6	3.597731000	-3.337338000	-0.780097000
1	4.418556000	-4.002337000	-1.020647000
6	0.227952000	0.075669000	4.566276000
1	-0.355086000	-0.147841000	5.450691000
6	-0.971573000	-2.208191000	-2.056131000

1	-0.097987000	-2.756818000	-2.413324000
1	-1.839274000	-2.677239000	-2.525356000
6	1.425318000	0.773998000	4.664671000
1	1.791832000	1.102351000	5.630166000
6	-1.105856000	-0.401758000	-3.825620000
1	-1.467705000	-1.137831000	-4.533402000
6	-0.217006000	1.396936000	-1.964479000
1	0.116895000	2.063484000	-1.181601000
6	-2.632147000	-0.223038000	2.500977000
1	-3.594126000	-0.733884000	2.522709000
1	-2.755156000	0.738394000	2.998736000
6	-2.442617000	-2.492066000	-0.098090000
1	-2.413474000	-2.861644000	0.926105000
1	-2.983782000	-3.235850000	-0.692967000
6	-3.173605000	-1.159617000	-0.157579000
1	-4.177136000	-1.241121000	0.258450000
1	-3.272292000	-0.792865000	-1.179335000
16	-2.282577000	0.184246000	0.739894000
6	-1.556941000	-1.055293000	3.205374000
1	-1.425308000	-2.020650000	2.714030000
1	-1.923121000	-1.278303000	4.209257000
8	1.055123000	3.277177000	0.483145000
6	3.766715000	4.520991000	1.646787000
6	4.625028000	5.774142000	1.845049000
6	5.709117000	5.893103000	0.769722000
6	5.105211000	5.852325000	-0.637742000
6	4.252410000	4.597149000	-0.845871000
6	3.156685000	4.494393000	0.222698000
1	6.278321000	6.817136000	0.910949000
1	3.979635000	6.660735000	1.813575000
1	5.075597000	5.751212000	2.842021000
1	4.382499000	3.624165000	1.779244000
1	2.975198000	4.478385000	2.400711000
1	4.485513000	6.744390000	-0.794378000
1	5.896320000	5.887595000	-1.392855000
1	3.799779000	4.601190000	-1.841834000
1	4.884565000	3.705509000	-0.789480000
1	2.486612000	5.357612000	0.125953000
1	6.421967000	5.066178000	0.881468000
6	2.343333000	3.230843000	0.067847000
1	0.804066000	4.163601000	0.780317000
8	2.769934000	2.178045000	-0.351687000

^{5}**TS1**$_{CCA,ald}$:

26	0.111853000	-0.309339000	-0.661874000
7	-0.127875000	0.527717000	1.460755000
6	1.076407000	0.514788000	2.069869000
1	1.855715000	-0.045543000	1.579055000
7	0.019746000	-1.193701000	-2.576846000
6	1.350731000	1.172778000	3.255031000
1	2.341992000	1.112594000	3.685394000
6	-0.227458000	-2.786697000	0.942135000
1	-0.441905000	-2.196788000	1.834781000
1	-0.546349000	-3.812934000	1.139892000
6	-0.408890000	-2.327687000	-5.055492000

1	-0.584651000	-2.773025000	-6.027439000
7	1.640021000	-1.648496000	-0.073530000
6	0.224173000	-1.090122000	-4.948849000
1	0.556513000	-0.548451000	-5.824821000
6	1.246776000	-2.723209000	0.638982000
6	3.897907000	-2.403338000	-0.009252000
1	4.932261000	-2.248004000	-0.286736000
7	-0.979647000	-2.195766000	-0.194215000
6	-1.122577000	1.243572000	2.035158000
6	2.932901000	-1.489196000	-0.399939000
1	3.171441000	-0.603952000	-0.974822000
6	-0.577291000	-2.394014000	-2.667129000
6	2.159839000	-3.677954000	1.061107000
1	1.822135000	-4.537919000	1.625446000
6	3.504163000	-3.511571000	0.737211000
1	4.234542000	-4.245395000	1.056143000
6	-0.905070000	1.939300000	3.227716000
1	-1.718618000	2.508211000	3.659165000
6	-0.899765000	-3.102161000	-1.379369000
1	-0.107946000	-3.832793000	-1.202298000
1	-1.824056000	-3.674637000	-1.475094000
6	0.332756000	1.906952000	3.850770000
1	0.499495000	2.447810000	4.774730000
6	-0.816793000	-2.986724000	-3.901900000
1	-1.310255000	-3.949483000	-3.952654000
6	0.414520000	-0.548921000	-3.689621000
1	0.879374000	0.414744000	-3.531935000
6	-2.614981000	1.730022000	-0.023768000
1	-3.641245000	2.020719000	-0.246942000
1	-1.968295000	2.582023000	-0.240383000
6	-2.383929000	-1.907699000	0.183312000
1	-2.361874000	-1.431764000	1.159059000
1	-2.944959000	-2.842003000	0.293651000
6	-3.090224000	-1.025627000	-0.837829000
1	-4.075226000	-0.731945000	-0.475695000
1	-3.241706000	-1.545203000	-1.783993000
16	-2.157117000	0.495841000	-1.293734000
6	-2.505556000	1.305787000	1.435591000
1	-3.034386000	0.365763000	1.602792000
1	-3.071430000	2.042152000	2.008670000
8	1.004331000	1.050769000	-1.134525000
6	2.937666000	4.728727000	1.500368000
6	4.398683000	5.145278000	1.705384000
6	5.326470000	3.930069000	1.804224000
6	5.187752000	3.023530000	0.576839000
6	3.735574000	2.583827000	0.366746000
6	2.805684000	3.807102000	0.281725000
1	6.365003000	4.256450000	1.916376000
1	4.715558000	5.777475000	0.866590000
1	4.477637000	5.761090000	2.606653000
1	2.577334000	4.195503000	2.387469000
1	2.298213000	5.606139000	1.377452000
1	5.534867000	3.559936000	-0.315043000
1	5.824701000	2.139848000	0.679162000
1	3.640773000	1.977680000	-0.537464000

1	3.424507000	1.954246000	1.206545000
1	3.072756000	4.363454000	-0.631453000
1	5.078963000	3.357071000	2.706975000
6	1.377997000	3.378989000	0.029203000
1	1.278035000	2.444528000	-0.676874000
8	0.376409000	3.847213000	0.486336000

^{5}**IM1**$_{CCA,ald}$:

26	0.182612000	-0.348125000	-0.777647000
7	0.044859000	0.582757000	1.267586000
6	1.273770000	0.545451000	1.831220000
1	2.036307000	0.011722000	1.286652000
7	-0.071501000	-1.348859000	-2.648497000
6	1.588026000	1.142618000	3.037166000
1	2.593885000	1.065036000	3.428739000
6	-0.293869000	-2.768322000	0.958481000
1	-0.455062000	-2.117009000	1.819715000
1	-0.660838000	-3.762692000	1.225988000
6	-0.641623000	-2.569232000	-5.057059000
1	-0.872880000	-3.048757000	-6.000783000
7	1.614032000	-1.801483000	-0.155527000
6	0.043747000	-1.355388000	-5.029769000
1	0.362656000	-0.866233000	-5.940969000
6	1.175005000	-2.806465000	0.626112000
6	3.823494000	-2.691186000	-0.096343000
1	4.857764000	-2.617885000	-0.406101000
7	-1.036289000	-2.203979000	-0.193339000
6	-0.926664000	1.279772000	1.903972000
6	2.906094000	-1.743269000	-0.519760000
1	3.181819000	-0.909200000	-1.152537000
6	-0.719345000	-2.524084000	-2.663197000
6	2.038933000	-3.789458000	1.086018000
1	1.663672000	-4.592567000	1.707598000
6	3.382271000	-3.726354000	0.724809000
1	4.074552000	-4.484013000	1.071917000
6	-0.667370000	1.906357000	3.126155000
1	-1.463163000	2.457219000	3.610597000
6	-1.022144000	-3.162538000	-1.332757000
1	-0.246269000	-3.908601000	-1.148478000
1	-1.965202000	-3.711551000	-1.376665000
6	0.587731000	1.835615000	3.708363000
1	0.784285000	2.322913000	4.655858000
6	-1.030336000	-3.159654000	-3.860489000
1	-1.564076000	-4.102012000	-3.849990000
6	0.304801000	-0.769869000	-3.803307000
1	0.815925000	0.178375000	-3.703341000
6	-2.471490000	1.798920000	-0.115544000
1	-3.496658000	2.114021000	-0.308137000
1	-1.809380000	2.625963000	-0.373640000
6	-2.411727000	-1.820284000	0.188853000
1	-2.345944000	-1.303282000	1.141138000
1	-3.027331000	-2.712410000	0.349763000
6	-3.084198000	-0.942258000	-0.858487000
1	-4.052101000	-0.591283000	-0.500999000
1	-3.265178000	-1.485841000	-1.785620000

16	-2.092400000	0.521820000	-1.368364000
6	-2.324497000	1.408483000	1.348586000
1	-2.902388000	0.508497000	1.566524000
1	-2.821551000	2.193244000	1.921638000
8	1.260642000	0.951807000	-1.450641000
6	2.814807000	4.715436000	1.819529000
6	4.211621000	5.273606000	2.113419000
6	5.285860000	4.184201000	2.036330000
6	5.262237000	3.475341000	0.678067000
6	3.877719000	2.896685000	0.370999000
6	2.801807000	3.989325000	0.461244000
1	6.274183000	4.616965000	2.218902000
1	4.442804000	6.065894000	1.390825000
1	4.212391000	5.743015000	3.102052000
1	2.529123000	4.005426000	2.602852000
1	2.067875000	5.512585000	1.822233000
1	5.538819000	4.187582000	-0.109428000
1	6.006777000	2.674331000	0.650892000
1	3.861060000	2.435595000	-0.619822000
1	3.647815000	2.106862000	1.092967000
1	2.985993000	4.719353000	-0.342200000
1	5.114497000	3.448203000	2.832176000
6	1.425040000	3.465661000	0.122102000
1	1.385812000	1.837231000	-1.045226000
8	0.352448000	3.817895000	0.469567000

^{5}**PR**$_{CCA,ald}$:

26	0.107112000	-0.391668000	0.233468000
7	-0.079330000	0.351948000	2.244323000
6	1.018504000	0.948173000	2.744289000
1	1.893746000	0.947660000	2.110821000
7	0.376676000	0.042396000	-1.848560000
6	1.063451000	1.544646000	3.991486000
1	1.978133000	2.008214000	4.337229000
6	-0.272595000	-3.379121000	0.119741000
1	-0.856091000	-3.396053000	1.042490000
1	-0.431606000	-4.339312000	-0.380823000
6	0.406877000	0.454314000	-4.589932000
1	0.415204000	0.611031000	-5.662059000
7	1.613605000	-1.921792000	0.560131000
6	0.670520000	1.506915000	-3.716954000
1	0.886358000	2.503330000	-4.080762000
6	1.185231000	-3.193743000	0.461962000
6	3.812080000	-2.703237000	1.083057000
1	4.842238000	-2.466280000	1.315740000
7	-0.750141000	-2.246460000	-0.705192000
6	-1.200966000	0.316988000	3.000058000
6	2.902045000	-1.683208000	0.854463000
1	3.206303000	-0.644791000	0.893859000
6	0.108107000	-0.973718000	-2.686542000
6	2.040236000	-4.269094000	0.670473000
1	1.668726000	-5.281509000	0.570995000
6	3.370084000	-4.020930000	0.993745000
1	4.055017000	-4.844064000	1.159251000
6	-1.226637000	0.909864000	4.262351000

1	-2.137857000	0.872383000	4.846017000
6	-0.152546000	-2.322585000	-2.065951000
1	0.806521000	-2.837846000	-1.983662000
1	-0.779306000	-2.931484000	-2.722584000
6	-0.090858000	1.530310000	4.766942000
1	-0.105495000	1.987958000	5.749050000
6	0.112997000	-0.799996000	-4.066716000
1	-0.118738000	-1.635117000	-4.716389000
6	0.637050000	1.259643000	-2.354086000
1	0.792195000	2.047342000	-1.630725000
6	-3.165674000	0.372145000	1.359241000
1	-4.106962000	-0.125410000	1.125437000
1	-3.402790000	1.386633000	1.680274000
6	-2.232126000	-2.206837000	-0.744848000
1	-2.597596000	-2.425761000	0.257275000
1	-2.610732000	-2.999187000	-1.401264000
6	-2.792063000	-0.870793000	-1.211026000
1	-3.881431000	-0.902957000	-1.193282000
1	-2.496041000	-0.632535000	-2.232305000
16	-2.247017000	0.575143000	-0.218870000
6	-2.429097000	-0.378756000	2.474328000
1	-2.175988000	-1.391546000	2.158976000
1	-3.134098000	-0.492597000	3.300617000
8	0.181248000	3.196781000	0.510420000
6	2.766790000	4.025210000	-0.959872000
6	3.967427000	3.489758000	-1.744105000
6	5.229708000	3.453283000	-0.877264000
6	5.000041000	2.658094000	0.412666000
6	3.794443000	3.188428000	1.196387000
6	2.537469000	3.185585000	0.320150000
1	6.063348000	3.024535000	-1.441976000
1	3.740240000	2.478848000	-2.105383000
1	4.130458000	4.109073000	-2.631314000
1	2.948347000	5.065714000	-0.667832000
1	1.868329000	4.027856000	-1.582109000
1	4.834216000	1.602178000	0.163477000
1	5.892875000	2.691071000	1.044178000
1	3.634181000	2.587483000	2.096354000
1	3.989324000	4.210363000	1.536356000
1	2.318961000	2.158085000	0.009468000
1	5.519942000	4.480005000	-0.621117000
6	1.317951000	3.704323000	1.040244000
1	-0.583371000	3.577701000	0.972025000
8	1.306932000	4.494613000	1.956640000

^{3}**TS1**$_{CCA,C\alpha}$:

26	3.649679000	1.190578000	6.389244000
7	5.302786000	2.383469000	6.851769000
6	6.432616000	2.012855000	6.200556000
1	6.330424000	1.249963000	5.451402000
7	2.084213000	0.016525000	6.010522000
6	7.678285000	2.561425000	6.427160000
1	8.527067000	2.196654000	5.863883000
6	5.139234000	-0.465072000	8.185494000
1	5.802295000	0.362260000	8.438851000

1	5.258084000	-1.233626000	8.951280000
6	-0.116216000	-1.618342000	5.660530000
1	-0.980274000	-2.258241000	5.528128000
7	4.819622000	-0.329246000	5.816123000
6	0.180126000	-0.624456000	4.729848000
1	-0.437878000	-0.466170000	3.855631000
6	5.450539000	-0.988764000	6.811570000
6	5.773951000	-1.799633000	4.208655000
1	5.868004000	-2.096979000	3.172609000
7	3.740456000	0.048943000	8.145869000
6	5.397547000	3.432572000	7.711091000
6	4.953452000	-0.734532000	4.544990000
1	4.390944000	-0.177947000	3.809201000
6	1.812699000	-0.944424000	6.909327000
6	6.283169000	-2.063172000	6.543928000
1	6.774063000	-2.581625000	7.357484000
6	6.458302000	-2.465127000	5.221614000
1	7.106980000	-3.300779000	4.988883000
6	6.635210000	4.026654000	7.976719000
1	6.671959000	4.852994000	8.674900000
6	2.786179000	-1.105101000	8.039123000
1	3.357382000	-2.019484000	7.873102000
1	2.264922000	-1.243540000	8.986973000
6	7.792155000	3.583026000	7.360567000
1	8.748036000	4.042472000	7.581020000
6	0.709766000	-1.777699000	6.765808000
1	0.510945000	-2.538792000	7.509906000
6	1.285776000	0.180478000	4.940264000
1	1.568982000	0.979756000	4.269792000
6	2.872394000	4.087856000	7.754757000
1	2.173001000	4.674819000	8.350188000
1	2.926431000	4.541179000	6.765573000
6	3.425971000	0.895433000	9.327202000
1	4.264754000	1.568611000	9.474525000
1	3.346792000	0.274822000	10.224817000
6	2.128962000	1.658287000	9.108177000
1	1.971354000	2.411691000	9.878992000
1	1.259764000	1.001587000	9.118325000
16	2.073694000	2.485166000	7.458655000
6	4.220691000	4.040921000	8.443239000
1	4.122756000	3.588405000	9.432748000
1	4.491140000	5.077687000	8.653039000
8	3.413847000	1.983809000	4.865421000
6	5.766196000	4.431371000	3.850261000
6	5.007221000	5.489469000	4.656056000
6	3.831128000	6.070909000	3.866069000
6	2.890939000	4.970012000	3.363702000
6	3.641533000	3.922807000	2.531152000
6	4.851591000	3.375019000	3.270592000
1	3.278139000	6.784207000	4.485064000
1	4.639720000	5.037329000	5.581522000
1	5.696635000	6.282725000	4.958162000
1	6.266904000	4.921635000	3.001538000
1	6.561842000	3.978252000	4.439311000
1	2.415217000	4.470609000	4.214715000

1	2.084852000	5.402518000	2.764285000
1	2.974224000	3.107206000	2.237598000
1	3.993264000	4.388354000	1.598808000
1	4.242500000	2.695637000	4.242359000
1	4.218206000	6.635466000	3.008547000
6	5.502001000	2.203972000	2.648530000
1	4.845282000	1.588986000	2.003137000
8	6.659384000	1.865663000	2.833943000

^{3}**IM1**$_{CCA,C\alpha}$:

26	3.507058000	1.085270000	6.510634000
7	5.091701000	2.407152000	6.884847000
6	6.166038000	2.139802000	6.100635000
1	6.032164000	1.398885000	5.334079000
7	2.016144000	-0.165331000	6.126543000
6	7.387692000	2.771684000	6.217010000
1	8.194276000	2.488989000	5.553658000
6	5.105122000	-0.476407000	8.256384000
1	5.730116000	0.386702000	8.488030000
1	5.277959000	-1.231190000	9.025469000
6	-0.088124000	-1.920936000	5.779918000
1	-0.914881000	-2.608599000	5.648632000
7	4.730185000	-0.363173000	5.897722000
6	0.149604000	-0.911255000	4.849620000
1	-0.478177000	-0.789166000	3.976556000
6	5.411746000	-0.992412000	6.878582000
6	5.732315000	-1.767153000	4.265461000
1	5.829955000	-2.048739000	3.225316000
7	3.678698000	-0.032746000	8.247654000
6	5.206380000	3.433704000	7.768973000
6	4.868842000	-0.741781000	4.620107000
1	4.284946000	-0.186515000	3.900324000
6	1.801130000	-1.140200000	7.024966000
6	6.288587000	-2.025252000	6.591643000
1	6.818876000	-2.523326000	7.393150000
6	6.457501000	-2.411243000	5.263170000
1	7.138584000	-3.215946000	5.014101000
6	6.423408000	4.100743000	7.933250000
1	6.478974000	4.904964000	8.655652000
6	2.783374000	-1.237797000	8.154247000
1	3.401650000	-2.122891000	7.999859000
1	2.270401000	-1.388714000	9.104284000
6	7.534123000	3.761222000	7.179887000
1	8.475772000	4.278597000	7.318254000
6	0.747860000	-2.035398000	6.883487000
1	0.592511000	-2.807528000	7.626667000
6	1.209239000	-0.044893000	5.057417000
1	1.451014000	0.767418000	4.387179000
6	2.682387000	3.979868000	7.962451000
1	1.981056000	4.507293000	8.609120000
1	2.690929000	4.493083000	7.000616000
6	3.345399000	0.783399000	9.443939000
1	4.160928000	1.482876000	9.593758000
1	3.296459000	0.147350000	10.332430000
6	2.019261000	1.500300000	9.245368000

1	1.836449000	2.230887000	10.032538000
1	1.176622000	0.809575000	9.245458000
16	1.926783000	2.362526000	7.614047000
6	4.055727000	3.946803000	8.603510000
1	4.016713000	3.432662000	9.565469000
1	4.295887000	4.979386000	8.864177000
8	3.284085000	1.850937000	4.887688000
6	6.178936000	4.559061000	3.285105000
6	5.556162000	5.421649000	4.404865000
6	4.340443000	6.201525000	3.899203000
6	3.291166000	5.273372000	3.282258000
6	3.898553000	4.385189000	2.171160000
6	5.142909000	3.708301000	2.636245000
1	3.894959000	6.773244000	4.719005000
1	5.257617000	4.772972000	5.235022000
1	6.315624000	6.099207000	4.803356000
1	6.599259000	5.234705000	2.525902000
1	6.998411000	3.947509000	3.661019000
1	2.857394000	4.633084000	4.059554000
1	2.461242000	5.849984000	2.865612000
1	3.167340000	3.659282000	1.806238000
1	4.153800000	5.035388000	1.321308000
1	3.632042000	2.749765000	4.844053000
1	4.665795000	6.930803000	3.147420000
6	5.310326000	2.298161000	2.489967000
1	4.478392000	1.761632000	2.003522000
8	6.307997000	1.661975000	2.870076000

^{3}**TS2**$_{CCA,C\alpha}$:

26	3.801522000	1.252528000	6.432636000
7	5.468986000	2.413536000	6.949809000
6	6.545644000	2.137795000	6.179292000
1	6.413606000	1.392314000	5.415718000
7	2.217174000	0.121379000	5.897171000
6	7.767560000	2.771256000	6.297809000
1	8.579695000	2.484764000	5.642187000
6	5.093042000	-0.483495000	8.347052000
1	5.711043000	0.350878000	8.681607000
1	5.171527000	-1.278028000	9.093654000
6	-0.029762000	-1.426753000	5.386454000
1	-0.913003000	-2.024554000	5.196422000
7	5.006450000	-0.308010000	5.943719000
6	0.368239000	-0.440055000	4.488188000
1	-0.181906000	-0.247367000	3.576447000
6	5.545266000	-0.966486000	6.992725000
6	6.103704000	-1.793566000	4.436016000
1	6.284774000	-2.098837000	3.413694000
7	3.699622000	0.002321000	8.200101000
6	5.581688000	3.428553000	7.844618000
6	5.255481000	-0.727649000	4.695296000
1	4.768787000	-0.178648000	3.904482000
6	1.858535000	-0.856747000	6.749784000
6	6.406438000	-2.036914000	6.805808000
1	6.822279000	-2.550707000	7.663392000
6	6.702505000	-2.447269000	5.508153000

1	7.372987000	-3.281442000	5.339936000
6	6.791967000	4.109532000	8.002597000
1	6.846752000	4.912364000	8.726378000
6	2.776238000	-1.129368000	7.907771000
1	3.368373000	-2.011517000	7.657770000
1	2.205248000	-1.391547000	8.800242000
6	7.902877000	3.777330000	7.244773000
1	8.839766000	4.305500000	7.375708000
6	0.729881000	-1.638400000	6.529147000
1	0.462748000	-2.405141000	7.245861000
6	1.491934000	0.312732000	4.779644000
1	1.839964000	1.080045000	4.106225000
6	3.087803000	4.107575000	8.043828000
1	2.402633000	4.597860000	8.735602000
1	3.174241000	4.743208000	7.162587000
6	3.226134000	0.788301000	9.359462000
1	4.048772000	1.411143000	9.693207000
1	2.969130000	0.126763000	10.193417000
6	2.006437000	1.625404000	8.991425000
1	1.742216000	2.313340000	9.794102000
1	1.133360000	1.002580000	8.798936000
16	2.226438000	2.615798000	7.449127000
6	4.435315000	3.890699000	8.714356000
1	4.329400000	3.242545000	9.584347000
1	4.725273000	4.856226000	9.132220000
8	3.840344000	2.237440000	4.752437000
6	5.539441000	4.273324000	3.634747000
6	4.869356000	5.315621000	4.537849000
6	3.669210000	5.971231000	3.849886000
6	2.648365000	4.930522000	3.378237000
6	3.299223000	3.859033000	2.488234000
6	4.573392000	3.304866000	3.045146000
1	3.187889000	6.682316000	4.527629000
1	4.547756000	4.821569000	5.457373000
1	5.604748000	6.070678000	4.826992000
1	6.011973000	4.790749000	2.783728000
1	6.343178000	3.746349000	4.144284000
1	2.173555000	4.463896000	4.249400000
1	1.838886000	5.410119000	2.822313000
1	2.598141000	3.056995000	2.238909000
1	3.562971000	4.328125000	1.527005000
1	2.976954000	2.658481000	4.658138000
1	4.019210000	6.550259000	2.986456000
6	5.055311000	2.031901000	2.529266000
1	4.302656000	1.420861000	1.999934000
8	6.202011000	1.617174000	2.668498000

^{3}**PR**$_{CCA,C\alpha}$:

26	3.883035000	1.139606000	6.542284000
7	5.461255000	2.461969000	6.952032000
6	6.547984000	2.278400000	6.170903000
1	6.505554000	1.457110000	5.477606000
7	2.347269000	-0.071009000	5.942019000
6	7.678993000	3.072086000	6.209343000
1	8.503877000	2.855515000	5.542944000

6	5.105704000	-0.603155000	8.523397000
1	5.655279000	0.261465000	8.900658000
1	5.223886000	-1.411482000	9.251850000
6	0.251921000	-1.743689000	5.223949000
1	-0.568048000	-2.396990000	4.950458000
7	5.187526000	-0.339453000	6.111855000
6	0.601479000	-0.664286000	4.415866000
1	0.071361000	-0.447526000	3.497440000
6	5.662100000	-1.016948000	7.180439000
6	6.474903000	-1.729538000	4.653992000
1	6.758417000	-1.986529000	3.641625000
7	3.695476000	-0.213393000	8.334800000
6	5.491259000	3.499434000	7.829352000
6	5.570735000	-0.702732000	4.876037000
1	5.143286000	-0.140976000	4.055044000
6	2.020130000	-1.116253000	6.721126000
6	6.574554000	-2.052218000	7.029217000
1	6.935233000	-2.580895000	7.902724000
6	6.996558000	-2.407625000	5.751648000
1	7.707244000	-3.213892000	5.615342000
6	6.602203000	4.341349000	7.907493000
1	6.587388000	5.160393000	8.614688000
6	2.857601000	-1.368810000	7.949419000
1	3.501042000	-2.226850000	7.743995000
1	2.215636000	-1.669817000	8.781163000
6	7.710294000	4.135218000	7.100261000
1	8.570879000	4.790344000	7.164512000
6	0.968562000	-1.966378000	6.391969000
1	0.723636000	-2.792044000	7.049160000
6	1.646904000	0.149630000	4.813855000
1	1.952253000	1.007800000	4.236223000
6	2.971945000	3.941724000	8.192015000
1	2.279999000	4.315202000	8.946930000
1	2.982343000	4.659480000	7.371444000
6	3.104591000	0.555348000	9.443357000
1	3.877831000	1.189033000	9.866840000
1	2.768970000	-0.109367000	10.248517000
6	1.908406000	1.386312000	8.978098000
1	1.555253000	2.039302000	9.775829000
1	1.071876000	0.749918000	8.689885000
16	2.212416000	2.440551000	7.488790000
6	4.359976000	3.765560000	8.790875000
1	4.345658000	2.997267000	9.564196000
1	4.593957000	4.690628000	9.320479000
8	3.446366000	2.768156000	4.499437000
6	5.364026000	3.759385000	3.364307000
6	5.426847000	5.017750000	4.231779000
6	4.518839000	6.119654000	3.677056000
6	3.077998000	5.624422000	3.520279000
6	3.012654000	4.348396000	2.667741000
6	3.937014000	3.248659000	3.213015000
1	4.543868000	6.996951000	4.330264000
1	5.121218000	4.773425000	5.252870000
1	6.460557000	5.367843000	4.291889000
1	5.746537000	3.977621000	2.362438000

1	5.993345000	2.964801000	3.764147000
1	2.643453000	5.444835000	4.511091000
1	2.452286000	6.390682000	3.054780000
1	1.986460000	3.971091000	2.599870000
1	3.329148000	4.578255000	1.645182000
1	2.616431000	3.213288000	4.709355000
1	4.896908000	6.444630000	2.699684000
6	3.843667000	2.026608000	2.306323000
1	2.829430000	1.586453000	2.242478000
8	4.765854000	1.533259000	1.702269000

^{5}**TS1**$_{CCA,C\alpha}$:

26	0.494875000	-0.079226000	-0.386881000
7	0.843713000	-0.030461000	1.839135000
6	2.163446000	-0.195352000	2.073559000
1	2.761159000	-0.527671000	1.240869000
7	-0.157714000	-0.237299000	-2.407497000
6	2.767563000	0.043462000	3.293564000
1	3.832635000	-0.113610000	3.402950000
6	0.402591000	-2.974411000	0.274412000
1	0.447886000	-2.730321000	1.337237000
1	0.086034000	-4.016491000	0.186841000
6	-1.273126000	-0.429892000	-4.925505000
1	-1.718224000	-0.506041000	-5.910360000
7	2.022949000	-1.495079000	-0.723417000
6	-0.572368000	0.716163000	-4.552606000
1	-0.451821000	1.550730000	-5.230931000
6	1.757343000	-2.762903000	-0.349185000
6	4.174191000	-2.149197000	-1.505558000
1	5.112641000	-1.873270000	-1.968264000
7	-0.569734000	-2.054322000	-0.368585000
6	0.064850000	0.404008000	2.860202000
6	3.198068000	-1.188338000	-1.297275000
1	3.333304000	-0.150318000	-1.572498000
6	-0.815741000	-1.352423000	-2.763037000
6	2.689962000	-3.773266000	-0.529120000
1	2.456169000	-4.786542000	-0.227666000
6	3.916768000	-3.459571000	-1.109349000
1	4.659910000	-4.233459000	-1.259393000
6	0.621727000	0.678767000	4.111698000
1	-0.023242000	1.034216000	4.904781000
6	-0.824625000	-2.486019000	-1.774682000
1	-0.038946000	-3.183645000	-2.071225000
1	-1.762354000	-3.042010000	-1.831500000
6	1.977455000	0.499085000	4.340815000
1	2.405606000	0.714946000	5.312355000
6	-1.397940000	-1.476485000	-4.019759000
1	-1.934573000	-2.380464000	-4.279757000
6	-0.033783000	0.778678000	-3.279677000
1	0.505566000	1.642715000	-2.918091000
6	-1.932564000	1.469172000	1.591019000
1	-2.985294000	1.701210000	1.752472000
1	-1.387925000	2.413292000	1.551257000
6	-1.827653000	-1.967216000	0.409043000
1	-1.554175000	-1.876979000	1.455836000

1	-2.399014000	-2.895981000	0.307071000
6	-2.700057000	-0.799737000	-0.033370000
1	-3.555574000	-0.686866000	0.632091000
1	-3.094842000	-0.948083000	-1.038158000
16	-1.819039000	0.816229000	-0.112266000
6	-1.427258000	0.572275000	2.713978000
1	-1.911528000	-0.404184000	2.667814000
1	-1.791925000	1.014357000	3.642582000
8	1.369185000	1.363240000	-0.644470000
6	3.866581000	3.506198000	-0.419135000
6	4.230930000	2.771018000	0.876661000
6	3.616806000	3.445467000	2.107145000
6	2.097763000	3.586500000	1.970889000
6	1.713272000	4.336213000	0.690196000
6	2.371996000	3.725489000	-0.536373000
1	3.858394000	2.873895000	3.008165000
1	3.871226000	1.738999000	0.809851000
1	5.319024000	2.717389000	0.973301000
1	4.354725000	4.491322000	-0.431782000
1	4.238000000	2.969407000	-1.295212000
1	1.639369000	2.594113000	1.946778000
1	1.680878000	4.104557000	2.838838000
1	0.626723000	4.363863000	0.562862000
1	2.043290000	5.382356000	0.769266000
1	1.878928000	2.597513000	-0.608676000
1	4.063563000	4.439017000	2.238950000
6	1.873098000	4.252700000	-1.838873000
1	0.814370000	4.576338000	-1.839183000
8	2.529425000	4.311762000	-2.859239000

^{5}**IM1**$_{CCA,C\alpha}$:

26	0.779943000	-0.277009000	-0.437433000
7	1.214949000	-0.137413000	1.753214000
6	2.544207000	-0.296144000	1.936492000
1	3.109399000	-0.648201000	1.087860000
7	0.089273000	-0.286130000	-2.465771000
6	3.193631000	-0.018309000	3.124775000
1	4.263246000	-0.165189000	3.197649000
6	0.022078000	-3.120465000	0.264146000
1	0.004919000	-2.870775000	1.326269000
1	-0.473203000	-4.088826000	0.149606000
6	-1.021338000	-0.255522000	-4.993886000
1	-1.464734000	-0.243334000	-5.982473000
7	1.976065000	-2.022663000	-0.634316000
6	-0.200837000	0.789026000	-4.573119000
1	0.015709000	1.631712000	-5.216849000
6	1.448269000	-3.189722000	-0.217162000
6	4.016267000	-3.121103000	-1.192106000
1	5.019603000	-3.058416000	-1.592467000
7	-0.685629000	-2.053046000	-0.483685000
6	0.472122000	0.310171000	2.795287000
6	3.227314000	-1.984882000	-1.124934000
1	3.575826000	-1.014317000	-1.454414000
6	-0.692130000	-1.302717000	-2.866419000
6	2.184388000	-4.364894000	-0.252272000

1	1.737552000	-5.292441000	0.082759000
6	3.488442000	-4.327754000	-0.737979000
1	4.080625000	-5.234148000	-0.775649000
6	1.073318000	0.613893000	4.017641000
1	0.457294000	0.979879000	4.828605000
6	-0.840833000	-2.453095000	-1.910785000
1	-0.063590000	-3.180080000	-2.153253000
1	-1.795953000	-2.961150000	-2.058393000
6	2.440329000	0.455279000	4.192008000
1	2.906064000	0.698191000	5.139544000
6	-1.271602000	-1.315406000	-4.128977000
1	-1.906469000	-2.141519000	-4.424236000
6	0.335786000	0.742933000	-3.297379000
1	0.967627000	1.528436000	-2.906757000
6	-1.519708000	1.474802000	1.639911000
1	-2.578549000	1.679328000	1.797530000
1	-0.988132000	2.422737000	1.719784000
6	-1.996776000	-1.731979000	0.125691000
1	-1.876534000	-1.756133000	1.203693000
1	-2.730569000	-2.505192000	-0.125835000
6	-2.535116000	-0.380625000	-0.326054000
1	-3.443855000	-0.131749000	0.221441000
1	-2.786523000	-0.376969000	-1.385880000
16	-1.357448000	1.018718000	-0.124849000
6	-1.020943000	0.473859000	2.678558000
1	-1.496504000	-0.495679000	2.538173000
1	-1.386151000	0.828628000	3.643794000
8	2.025674000	1.003630000	-0.848296000
6	3.354263000	4.561414000	-0.424384000
6	4.102549000	3.729290000	0.643152000
6	3.502585000	3.916589000	2.039353000
6	2.000204000	3.627061000	2.052266000
6	1.256637000	4.490888000	1.009580000
6	1.881087000	4.353800000	-0.336295000
1	4.013353000	3.265392000	2.754842000
1	4.073171000	2.668205000	0.365277000
1	5.159364000	4.008348000	0.637315000
1	3.563330000	5.624230000	-0.234245000
1	3.715647000	4.336729000	-1.428526000
1	1.827321000	2.569314000	1.829873000
1	1.576287000	3.806085000	3.043261000
1	0.191262000	4.249484000	0.976164000
1	1.329990000	5.541330000	1.326342000
1	2.391180000	1.645206000	-0.230435000
1	3.674619000	4.946982000	2.372500000
6	1.092900000	4.009371000	-1.476068000
1	0.019396000	3.837323000	-1.284794000
8	1.530974000	3.894259000	-2.632056000

[5]**PR**$_{CCA,C\alpha}$:

26	3.961189000	1.220590000	6.287739000
7	5.714321000	2.475552000	7.074378000
6	6.783945000	2.395635000	6.261356000
1	6.804083000	1.552668000	5.586394000
7	2.128041000	0.134291000	5.689822000

6	7.820049000	3.315446000	6.247090000
1	8.647090000	3.184806000	5.560847000
6	5.012237000	-0.664073000	8.369878000
1	5.532286000	0.170630000	8.844419000
1	4.993545000	-1.490178000	9.087244000
6	-0.298153000	-1.189459000	5.376235000
1	-1.243391000	-1.706285000	5.259614000
7	5.398098000	-0.425453000	5.991266000
6	0.021497000	-0.100401000	4.570924000
1	-0.658986000	0.261745000	3.810888000
6	5.752864000	-1.067155000	7.119195000
6	6.985652000	-1.726222000	4.760914000
1	7.439389000	-1.961614000	3.806811000
7	3.646586000	-0.211541000	8.019794000
6	5.674229000	3.495139000	7.966530000
6	5.994166000	-0.759558000	4.835855000
1	5.664991000	-0.230949000	3.950502000
6	1.816230000	-0.912842000	6.474104000
6	6.735293000	-2.049936000	7.124622000
1	6.988826000	-2.554391000	8.048982000
6	7.369215000	-2.377169000	5.930435000
1	8.139353000	-3.139128000	5.910668000
6	6.669351000	4.473706000	7.986600000
1	6.599165000	5.285049000	8.700270000
6	2.855945000	-1.346111000	7.472132000
1	3.539509000	-2.026456000	6.962685000
1	2.395959000	-1.914786000	8.284189000
6	7.749447000	4.395864000	7.116554000
1	8.524387000	5.153311000	7.131278000
6	0.610854000	-1.595269000	6.347037000
1	0.390598000	-2.426345000	7.005989000
6	1.238680000	0.534244000	4.767831000
1	1.514892000	1.401240000	4.185345000
6	3.181546000	3.904393000	8.560147000
1	2.557193000	4.133479000	9.424212000
1	3.193250000	4.788943000	7.921322000
6	2.969040000	0.408079000	9.181853000
1	3.717943000	0.941807000	9.758867000
1	2.565897000	-0.369065000	9.840732000
6	1.835835000	1.353624000	8.794794000
1	1.448311000	1.850372000	9.684319000
1	1.002915000	0.823461000	8.335052000
16	2.283964000	2.650345000	7.576176000
6	4.595673000	3.545754000	9.018254000
1	4.589386000	2.605142000	9.566381000
1	4.884636000	4.303979000	9.748984000
8	3.567713000	2.671223000	4.707990000
6	5.380510000	3.749037000	3.440562000
6	5.230269000	5.100046000	4.142835000
6	4.166503000	5.965340000	3.457699000
6	2.826298000	5.230978000	3.346416000
6	2.979726000	3.864468000	2.662393000
6	4.049444000	3.004363000	3.364486000
1	4.032618000	6.904413000	4.001525000
1	4.966624000	4.944518000	5.195302000

1	6.194820000	5.613520000	4.146446000
1	5.731968000	3.894149000	2.415617000
1	6.119613000	3.123039000	3.938168000
1	2.379281000	5.120060000	4.344470000
1	2.104634000	5.824013000	2.779155000
1	2.025456000	3.331077000	2.631820000
1	3.301657000	4.006376000	1.627310000
1	3.025344000	3.395756000	5.044702000
1	4.512091000	6.230018000	2.451223000
6	4.158412000	1.675121000	2.637802000
1	3.456662000	0.891991000	2.979756000
8	4.922829000	1.470541000	1.727011000

Cartesian coordinates of UBP86-GD3BJ/BS3 optimized geometries:

^{2}2a:

26	-0.001140000	0.002637000	-0.062983000
7	0.038681000	0.086398000	2.010881000
6	1.299736000	0.101058000	2.531356000
1	2.115263000	0.000728000	1.827234000
7	-0.034297000	-0.069924000	-2.028082000
6	1.594585000	0.247531000	3.881672000
1	2.638393000	0.236421000	4.200606000
6	0.479978000	-2.692257000	0.633311000
1	0.267074000	-2.472195000	1.690037000
1	0.419432000	-3.783206000	0.493981000
6	-0.170817000	-0.321715000	-4.796827000
1	-0.227219000	-0.422496000	-5.883045000
7	1.783295000	-0.830636000	-0.145480000
6	0.094740000	0.920824000	-4.207559000
1	0.252066000	1.816435000	-4.811156000
6	1.821045000	-2.141989000	0.228958000
6	4.120830000	-0.911045000	-0.665686000
1	5.007351000	-0.392398000	-1.034796000
7	-0.535339000	-1.975093000	-0.202071000
6	-0.995265000	0.283304000	2.889773000
6	2.904196000	-0.232441000	-0.608461000
1	2.802173000	0.812485000	-0.899703000
6	-0.286125000	-1.278113000	-2.588878000
6	3.005979000	-2.873683000	0.192902000
1	3.004356000	-3.923147000	0.494427000
6	4.177493000	-2.245304000	-0.247588000
1	5.118723000	-2.798569000	-0.281605000
6	-0.750096000	0.443315000	4.264612000
1	-1.602912000	0.597535000	4.928601000
6	-0.413174000	-2.431775000	-1.636479000
1	0.483140000	-3.064289000	-1.729736000
1	-1.268175000	-3.073772000	-1.896506000
6	0.542881000	0.416076000	4.781255000
1	0.720134000	0.538786000	5.852278000
6	-0.367422000	-1.433965000	-3.974716000
1	-0.577008000	-2.419503000	-4.396102000
6	0.149515000	1.013377000	-2.820143000
1	0.336751000	1.950108000	-2.293741000
6	-2.804904000	1.180657000	1.264828000
1	-3.896553000	1.272475000	1.165920000
1	-2.386941000	2.195299000	1.332957000
6	-1.925609000	-2.178838000	0.302689000
1	-1.891224000	-2.055880000	1.390530000
1	-2.255135000	-3.212386000	0.098725000
6	-2.874687000	-1.183967000	-0.352632000
1	-3.859293000	-1.177914000	0.135405000
1	-3.040616000	-1.408922000	-1.415747000
16	-2.192491000	0.541461000	-0.328472000
6	-2.450073000	0.334820000	2.476539000
1	-2.858483000	-0.682591000	2.366257000
1	-3.008113000	0.754761000	3.327226000
8	0.416054000	1.751891000	-0.098087000

8	1.618639000	2.106616000	0.639647000
6	1.528005000	3.506656000	1.089916000
6	2.867064000	3.677132000	1.812855000
1	2.942938000	4.703915000	2.202621000
1	2.949353000	2.979813000	2.660564000
1	3.711001000	3.505518000	1.127376000
6	1.410835000	4.428526000	-0.125605000
1	2.250655000	4.264996000	-0.818864000
1	0.468452000	4.250138000	-0.663598000
1	1.427535000	5.481502000	0.196517000
6	0.345088000	3.657860000	2.048543000
1	-0.603412000	3.481124000	1.522165000
1	0.426103000	2.950532000	2.887462000
1	0.319372000	4.679037000	2.459125000

^{4}2a:

26	0.064195000	-0.060691000	-0.109289000
7	0.056681000	-0.072834000	2.261586000
6	1.332940000	-0.058302000	2.716223000
1	2.108445000	-0.275418000	1.984275000
7	-0.251476000	-0.134087000	-2.246081000
6	1.683022000	0.223620000	4.035117000
1	2.733553000	0.205506000	4.331678000
6	0.541712000	-2.819997000	0.472052000
1	0.377166000	-2.636663000	1.544822000
1	0.450499000	-3.903282000	0.293572000
6	-1.156712000	-0.404857000	-4.853479000
1	-1.521187000	-0.511451000	-5.878021000
7	1.903184000	-0.958811000	-0.241180000
6	-0.721145000	0.841160000	-4.381845000
1	-0.731195000	1.725829000	-5.021158000
6	1.889056000	-2.289814000	0.057423000
6	4.239790000	-1.088308000	-0.752433000
1	5.148644000	-0.582600000	-1.082868000
7	-0.502537000	-2.055858000	-0.286422000
6	-0.940798000	0.220913000	3.140105000
6	3.049035000	-0.370798000	-0.652133000
1	2.984073000	0.694153000	-0.875022000
6	-0.654882000	-1.342627000	-2.697081000
6	3.046891000	-3.061238000	-0.020756000
1	3.010462000	-4.125370000	0.220368000
6	4.240474000	-2.448713000	-0.423418000
1	5.159978000	-3.034551000	-0.492856000
6	-0.653572000	0.536344000	4.478817000
1	-1.475958000	0.778449000	5.155624000
6	-0.474178000	-2.486356000	-1.734843000
1	0.513277000	-2.934192000	-1.930161000
1	-1.217714000	-3.281080000	-1.897695000
6	0.664590000	0.535896000	4.938663000
1	0.889113000	0.778312000	5.980369000
6	-1.128211000	-1.513876000	-4.000906000
1	-1.464095000	-2.496090000	-4.340280000
6	-0.284872000	0.940023000	-3.061812000
1	0.040245000	1.882922000	-2.615637000
6	-2.793926000	1.043918000	1.518850000

1	-3.889568000	1.123679000	1.466519000
1	-2.389888000	2.063877000	1.594615000
6	-1.857841000	-2.259432000	0.317650000
1	-1.736002000	-2.144266000	1.400688000
1	-2.197223000	-3.292709000	0.128064000
6	-2.872735000	-1.266542000	-0.230036000
1	-3.824248000	-1.332557000	0.315833000
1	-3.096064000	-1.440245000	-1.292190000
16	-2.270135000	0.484130000	-0.149375000
6	-2.390141000	0.181165000	2.713215000
1	-2.717137000	-0.859939000	2.560172000
1	-2.993607000	0.536045000	3.563306000
8	0.488438000	1.675690000	-0.108067000
8	1.721434000	1.974187000	0.609521000
6	1.656299000	3.349289000	1.139704000
6	2.995705000	3.448665000	1.874630000
1	3.098902000	4.455309000	2.308353000
1	3.052053000	2.713059000	2.691310000
1	3.838391000	3.282583000	1.186181000
6	1.565358000	4.333240000	-0.028574000
1	2.412759000	4.197128000	-0.718489000
1	0.629058000	4.187420000	-0.586935000
1	1.585586000	5.369013000	0.345083000
6	0.473584000	3.480608000	2.100210000
1	-0.476849000	3.344591000	1.565639000
1	0.539042000	2.738376000	2.908480000
1	0.468188000	4.484131000	2.552847000

[6]2a:

26	0.100260000	-0.033490000	-0.022198000
7	0.176231000	-0.049449000	2.209399000
6	1.467434000	0.071289000	2.614315000
1	2.230605000	-0.142952000	1.870213000
7	-0.249246000	-0.115613000	-2.135140000
6	1.847051000	0.439484000	3.902393000
1	2.907267000	0.500957000	4.154369000
6	0.372579000	-2.964895000	0.458878000
1	0.194135000	-2.782535000	1.530754000
1	0.263183000	-4.048083000	0.281368000
6	-0.980312000	-0.269201000	-4.812479000
1	-1.276763000	-0.331788000	-5.862278000
7	1.871351000	-1.169836000	-0.181748000
6	-0.565714000	0.951106000	-4.261584000
1	-0.523102000	1.861939000	-4.861494000
6	1.760302000	-2.495686000	0.088428000
6	4.211317000	-1.433717000	-0.628506000
1	5.160465000	-0.981840000	-0.921646000
7	-0.626484000	-2.174988000	-0.310562000
6	-0.806644000	0.204420000	3.123522000
6	3.064928000	-0.646256000	-0.541469000
1	3.076609000	0.427762000	-0.741886000
6	-0.632767000	-1.300542000	-2.662868000
6	2.869584000	-3.339372000	0.022048000
1	2.757921000	-4.404118000	0.237111000
6	4.111362000	-2.799376000	-0.334102000

1	4.991973000	-3.443312000	-0.393120000
6	-0.485155000	0.607450000	4.428576000
1	-1.294697000	0.815293000	5.131296000
6	-0.543741000	-2.508484000	-1.763577000
1	0.431191000	-2.986672000	-1.954331000
1	-1.308711000	-3.255158000	-2.031602000
6	0.846545000	0.727990000	4.831159000
1	1.092787000	1.036145000	5.850190000
6	-1.016157000	-1.408281000	-4.003353000
1	-1.334504000	-2.374222000	-4.401817000
6	-0.216696000	0.990667000	-2.914476000
1	0.094076000	1.910043000	-2.413800000
6	-2.848923000	0.859654000	1.661376000
1	-3.946954000	0.802821000	1.677882000
1	-2.567828000	1.917159000	1.772672000
6	-1.999017000	-2.354385000	0.218962000
1	-1.930536000	-2.331452000	1.312555000
1	-2.394864000	-3.348778000	-0.059093000
6	-2.957811000	-1.280147000	-0.290445000
1	-3.936505000	-1.367900000	0.202306000
1	-3.133887000	-1.365353000	-1.372459000
16	-2.350553000	0.447327000	-0.053261000
6	-2.264147000	0.003209000	2.785118000
1	-2.462273000	-1.062441000	2.598486000
1	-2.846224000	0.241892000	3.688657000
8	0.520373000	1.821519000	-0.086647000
8	1.713706000	2.157244000	0.611878000
6	1.630305000	3.558344000	1.094652000
6	2.975866000	3.714843000	1.804085000
1	3.052665000	4.733288000	2.214552000
1	3.069227000	3.000906000	2.636289000
1	3.812960000	3.561512000	1.106030000
6	1.493494000	4.492635000	-0.106611000
1	2.336300000	4.360139000	-0.802765000
1	0.554781000	4.298481000	-0.645374000
1	1.485582000	5.540425000	0.231844000
6	0.452631000	3.673360000	2.062922000
1	-0.494422000	3.468448000	1.543678000
1	0.561471000	2.966026000	2.898338000
1	0.404048000	4.692221000	2.476917000

$^{2}TS_{FeO}$:

26	0.058021000	-0.269678000	-0.046267000
7	0.176958000	0.005922000	2.007877000
6	1.460446000	0.101429000	2.466827000
1	2.244474000	-0.077627000	1.738158000
7	0.089982000	-0.224069000	-1.975672000
6	1.811595000	0.407852000	3.776186000
1	2.868154000	0.464326000	4.044672000
6	0.347446000	-2.964381000	0.567072000
1	0.126979000	-2.748103000	1.623690000
1	0.209505000	-4.044608000	0.400254000
6	0.089188000	-0.316628000	-4.755632000
1	0.086304000	-0.354812000	-5.847328000
7	1.785451000	-1.178574000	-0.120075000

6	0.402602000	0.873323000	-4.084792000
1	0.650081000	1.786141000	-4.629714000
6	1.733802000	-2.501872000	0.208160000
6	4.130863000	-1.397445000	-0.590537000
1	5.058556000	-0.925374000	-0.919180000
7	-0.603580000	-2.154994000	-0.265753000
6	-0.820384000	0.254643000	2.916890000
6	2.960348000	-0.642935000	-0.530261000
1	2.939728000	0.416684000	-0.793983000
6	-0.211687000	-1.380949000	-2.616165000
6	2.867147000	-3.311353000	0.165639000
1	2.789081000	-4.368274000	0.428957000
6	4.088386000	-2.749181000	-0.227917000
1	4.990907000	-3.363238000	-0.265591000
6	-0.521601000	0.560191000	4.255027000
1	-1.348653000	0.748731000	4.942558000
6	-0.484926000	-2.559978000	-1.722593000
1	0.339619000	-3.282746000	-1.818416000
1	-1.397656000	-3.089995000	-2.032765000
6	0.795205000	0.633628000	4.705633000
1	1.017808000	0.872194000	5.748232000
6	-0.226098000	-1.457170000	-4.010017000
1	-0.478496000	-2.400729000	-4.499149000
6	0.386565000	0.887981000	-2.692751000
1	0.599842000	1.787103000	-2.111005000
6	-2.678255000	1.082027000	1.343749000
1	-3.773121000	1.149408000	1.263435000
1	-2.278678000	2.103900000	1.422516000
6	-2.014366000	-2.291711000	0.210364000
1	-1.986917000	-2.243835000	1.304557000
1	-2.409192000	-3.284843000	-0.065982000
6	-2.881198000	-1.188582000	-0.382971000
1	-3.861080000	-1.136668000	0.111671000
1	-3.062675000	-1.342816000	-1.456438000
16	-2.066084000	0.480209000	-0.268089000
6	-2.280004000	0.218920000	2.535681000
1	-2.616539000	-0.817736000	2.384663000
1	-2.855594000	0.574783000	3.403775000
8	-0.685368000	3.276798000	-0.231770000
8	0.628735000	3.156040000	-0.091073000
6	1.216912000	4.034807000	1.023572000
6	2.658134000	3.548100000	1.076719000
1	3.215596000	4.139534000	1.818763000
1	2.709191000	2.491088000	1.376341000
1	3.155576000	3.667475000	0.102095000
6	1.084220000	5.478738000	0.552056000
1	1.624900000	5.641116000	-0.392776000
1	0.025359000	5.742291000	0.410381000
1	1.508223000	6.152544000	1.312551000
6	0.439187000	3.752112000	2.301055000
1	-0.613819000	4.048173000	2.196666000
1	0.488300000	2.687075000	2.565262000
1	0.878979000	4.329355000	3.128359000

^{2}TS$_{OO}$:

26	-0.426983000	0.038160000	-0.149076000
7	-0.427030000	0.140710000	1.921150000
6	0.813230000	0.400640000	2.423596000
1	1.592771000	0.645513000	1.710183000
7	-0.418639000	-0.162669000	-2.112716000
6	1.115800000	0.405486000	3.778069000
1	2.138516000	0.624551000	4.089210000
6	0.497553000	-2.561577000	0.641900000
1	0.250719000	-2.348986000	1.693200000
1	0.630080000	-3.649515000	0.539713000
6	-0.481418000	-0.539273000	-4.864294000
1	-0.509913000	-0.690281000	-5.945693000
7	1.470645000	-0.526943000	-0.187204000
6	-0.416711000	0.752219000	-4.325310000
1	-0.391320000	1.636904000	-4.963714000
6	1.725786000	-1.799737000	0.222399000
6	3.788633000	-0.188767000	-0.662740000
1	4.575392000	0.477493000	-1.019241000
7	-0.626966000	-2.063752000	-0.211908000
6	-1.453167000	-0.002603000	2.814061000
6	2.470428000	0.263136000	-0.632013000
1	2.174283000	1.266187000	-0.939545000
6	-0.472552000	-1.416583000	-2.620031000
6	3.023386000	-2.307074000	0.215123000
1	3.205185000	-3.333341000	0.540133000
6	4.070229000	-1.486949000	-0.225645000
1	5.094571000	-1.865901000	-0.237775000
6	-1.193594000	-0.032690000	4.195060000
1	-2.035793000	-0.172964000	4.876170000
6	-0.435839000	-2.542586000	-1.627598000
1	0.539756000	-3.047048000	-1.703785000
1	-1.192004000	-3.305011000	-1.867057000
6	0.095089000	0.140273000	4.692913000
1	0.286737000	0.109936000	5.768025000
6	-0.511300000	-1.635376000	-3.999929000
1	-0.559613000	-2.657179000	-4.382161000
6	-0.392283000	0.907050000	-2.943523000
1	-0.353385000	1.878978000	-2.450042000
6	-3.333827000	0.737878000	1.201242000
1	-4.429391000	0.737281000	1.105343000
1	-2.995802000	1.782788000	1.262064000
6	-1.963203000	-2.465168000	0.313698000
1	-1.947436000	-2.301161000	1.396430000
1	-2.131487000	-3.544140000	0.152124000
6	-3.059791000	-1.660278000	-0.373936000
1	-4.037624000	-1.803222000	0.106101000
1	-3.174760000	-1.935681000	-1.431878000
16	-2.689858000	0.156935000	-0.404395000
6	-2.910135000	-0.076049000	2.410931000
1	-3.249290000	-1.120297000	2.317289000
1	-3.488370000	0.312649000	3.264106000
8	-0.177229000	1.671439000	-0.242929000
8	3.167663000	2.274815000	1.483569000
6	2.703067000	3.556894000	1.364437000
6	3.765543000	4.366008000	2.229029000

1	3.461370000	5.423163000	2.184070000
1	3.751110000	4.018360000	3.270336000
1	4.771617000	4.247979000	1.805695000
6	2.769473000	4.064853000	-0.092831000
1	3.771895000	3.898655000	-0.515335000
1	2.035336000	3.522267000	-0.710192000
1	2.533589000	5.139078000	-0.146590000
6	1.306208000	3.750056000	1.987931000
1	0.571629000	3.156559000	1.420062000
1	1.298022000	3.412963000	3.035278000
1	1.003776000	4.808679000	1.953853000

^{2}Re$_{DMS}$:

26	0.134389000	-0.783687000	-0.369865000
7	-1.577154000	-0.175824000	0.644586000
6	-1.277800000	0.523277000	1.778086000
1	-0.232940000	0.780009000	1.920812000
7	1.710696000	-1.424898000	-1.351942000
6	-2.213067000	0.901940000	2.729965000
1	-1.883164000	1.449577000	3.614541000
6	-0.323520000	-2.798281000	1.565737000
1	-1.325879000	-2.401502000	1.782220000
1	-0.272891000	-3.825719000	1.960160000
6	3.758163000	-2.577363000	-2.862487000
1	4.557049000	-3.030706000	-3.453734000
7	1.149740000	-0.912816000	1.324029000
6	3.460583000	-1.215312000	-2.984685000
1	4.013465000	-0.569832000	-3.669531000
6	0.744950000	-1.917393000	2.153250000
6	2.772300000	-0.230253000	2.951486000
1	3.578206000	0.452780000	3.225924000
7	-0.120549000	-2.775610000	0.082995000
6	-2.900055000	-0.361659000	0.365128000
6	2.158608000	-0.097894000	1.705791000
1	2.432745000	0.677993000	0.989433000
6	1.987356000	-2.750161000	-1.238303000
6	1.320624000	-2.102163000	3.408311000
1	0.979551000	-2.921709000	4.044187000
6	2.340326000	-1.236469000	3.821977000
1	2.802573000	-1.359975000	4.803974000
6	-3.886819000	-0.041570000	1.315860000
1	-4.930486000	-0.251246000	1.070491000
6	1.158920000	-3.505383000	-0.244512000
1	1.742050000	-3.620458000	0.682210000
1	0.932615000	-4.522385000	-0.597915000
6	-3.553472000	0.560894000	2.523637000
1	-4.322580000	0.800774000	3.261569000
6	3.003270000	-3.354957000	-1.980744000
1	3.190022000	-4.424974000	-1.866404000
6	2.425740000	-0.673963000	-2.226404000
1	2.150008000	0.377131000	-2.286789000
6	-2.593809000	-0.422796000	-2.190292000
1	-3.133565000	-0.665132000	-3.117191000
1	-2.392193000	0.658926000	-2.175590000
6	-1.279357000	-3.369545000	-0.647356000

1	-2.188201000	-3.013085000	-0.151450000
1	-1.266900000	-4.468550000	-0.548838000
6	-1.242831000	-2.975345000	-2.119216000
1	-2.175098000	-3.241227000	-2.636553000
1	-0.418734000	-3.465359000	-2.657762000
16	-0.935845000	-1.162607000	-2.342149000
6	-3.399566000	-0.850821000	-0.976454000
1	-3.565068000	-1.939861000	-0.982585000
1	-4.408861000	-0.428008000	-1.104659000
8	0.251142000	0.967528000	-0.734609000
8	1.551112000	1.625867000	-0.681351000
6	1.328806000	3.086448000	-0.733823000
6	2.763373000	3.618122000	-0.696837000
1	2.742657000	4.718452000	-0.727745000
1	3.278540000	3.310849000	0.226380000
1	3.340990000	3.260021000	-1.562794000
6	0.617733000	3.445543000	-2.040038000
1	1.189071000	3.080954000	-2.908094000
1	-0.390037000	3.008216000	-2.066238000
1	0.519710000	4.539055000	-2.125675000
6	0.525221000	3.513807000	0.496013000
1	-0.461809000	3.030406000	0.488274000
1	1.048010000	3.237086000	1.424909000
1	0.377828000	4.604843000	0.493541000
16	-3.176778000	2.950880000	-0.642305000
6	-3.513827000	3.745899000	0.966803000
1	-2.545550000	3.920307000	1.455173000
1	-4.028272000	4.707740000	0.827388000
1	-4.118073000	3.084793000	1.604347000
6	-4.896659000	2.701084000	-1.210435000
1	-4.852973000	2.236864000	-2.205392000
1	-5.441233000	2.035679000	-0.524157000
1	-5.425966000	3.661621000	-1.290805000

$^{2}TS_{direct}$:

26	-0.558428000	-0.816162000	-0.279924000
7	-1.980203000	-0.302964000	1.116540000
6	-1.523131000	0.669982000	1.953768000
1	-0.498390000	0.992245000	1.799585000
7	0.858187000	-1.191154000	-1.613465000
6	-2.294999000	1.263495000	2.944372000
1	-1.850210000	2.035330000	3.574703000
6	-0.325104000	-3.004083000	1.552035000
1	-1.342593000	-2.801528000	1.918908000
1	-0.032815000	-4.006866000	1.903795000
6	2.791672000	-1.837485000	-3.506574000
1	3.543158000	-2.097260000	-4.255797000
7	0.773994000	-0.893279000	1.169592000
6	2.214052000	-0.563027000	-3.485690000
1	2.502349000	0.205842000	-4.204676000
6	0.650841000	-1.951680000	2.019376000
6	2.577362000	-0.026022000	2.465568000
1	3.349410000	0.735331000	2.593641000
7	-0.334808000	-2.919693000	0.057961000
6	-3.289694000	-0.669243000	1.223980000

6	1.735863000	0.035180000	1.353217000
1	1.818618000	0.814886000	0.578979000
6	1.430391000	-2.420287000	-1.608060000
6	1.455710000	-2.052977000	3.151506000
1	1.346433000	-2.910723000	3.818139000
6	2.418314000	-1.062599000	3.390595000
1	3.058859000	-1.123332000	4.273458000
6	-4.113860000	-0.105025000	2.211914000
1	-5.155213000	-0.427940000	2.267858000
6	1.010612000	-3.319065000	-0.485210000
1	1.756822000	-3.237175000	0.319857000
1	0.999011000	-4.375792000	-0.790862000
6	-3.623356000	0.857574000	3.091764000
1	-4.268825000	1.293237000	3.857912000
6	2.396063000	-2.777289000	-2.550329000
1	2.829270000	-3.779442000	-2.525196000
6	1.248585000	-0.270794000	-2.525600000
1	0.774558000	0.703513000	-2.424916000
6	-3.760978000	-1.344087000	-1.206038000
1	-4.337956000	-2.054872000	-1.815487000
1	-4.145793000	-0.340189000	-1.426980000
6	-1.397090000	-3.741730000	-0.590492000
1	-2.274989000	-3.743853000	0.059588000
1	-1.068572000	-4.790625000	-0.679516000
6	-1.725610000	-3.182906000	-1.967791000
1	-2.602052000	-3.677405000	-2.409069000
1	-0.891406000	-3.293238000	-2.674763000
16	-2.068043000	-1.366663000	-1.914604000
6	-3.909782000	-1.666130000	0.280734000
1	-3.563338000	-2.682441000	0.510128000
1	-4.990864000	-1.690068000	0.486259000
8	-0.458573000	0.807203000	-0.654985000
8	1.962531000	2.196256000	-0.796128000
6	1.958940000	3.482809000	-0.277822000
6	3.468864000	3.843133000	-0.082023000
1	3.543785000	4.856604000	0.344717000
1	3.948389000	3.127765000	0.602633000
1	3.994675000	3.822402000	-1.048402000
6	1.311659000	4.493789000	-1.247711000
1	1.778211000	4.418999000	-2.242872000
1	0.235888000	4.287619000	-1.351564000
1	1.421009000	5.529977000	-0.887123000
6	1.255814000	3.529198000	1.101872000
1	0.216490000	3.183485000	0.998237000
1	1.770885000	2.886438000	1.831500000
1	1.242418000	4.557694000	1.497729000
16	-2.088108000	2.687150000	-0.662245000
6	-3.912459000	2.555563000	-0.622853000
1	-4.198244000	2.209647000	0.378999000
1	-4.372329000	3.535736000	-0.817562000
1	-4.257773000	1.837059000	-1.377975000
6	-1.818798000	2.626667000	-2.462512000
1	-0.734829000	2.616568000	-2.623585000
1	-2.257322000	1.713594000	-2.887956000
1	-2.256589000	3.513990000	-2.941583000

^{2}I1$_{DMS}$:

26	-0.556640000	-0.060880000	-0.246222000
7	-0.637352000	0.196799000	1.809164000
6	0.532823000	0.690751000	2.306509000
1	1.325322000	0.914612000	1.600051000
7	-0.479438000	-0.396296000	-2.191175000
6	0.756291000	0.948941000	3.651927000
1	1.729057000	1.338923000	3.955592000
6	0.693693000	-2.432021000	0.771701000
1	0.368258000	-2.169486000	1.789533000
1	0.982908000	-3.494525000	0.772077000
6	-0.457653000	-0.976125000	-4.907607000
1	-0.452615000	-1.207529000	-5.975143000
7	1.404583000	-0.359521000	-0.215048000
6	-0.612044000	0.343713000	-4.464376000
1	-0.728877000	1.171503000	-5.165827000
6	1.819886000	-1.540746000	0.318398000
6	3.663329000	0.218116000	-0.747415000
1	4.359653000	0.927677000	-1.195034000
7	-0.446203000	-2.173385000	-0.164139000
6	-1.677747000	0.032094000	2.683069000
6	2.296713000	0.499382000	-0.750729000
1	1.876006000	1.410157000	-1.178825000
6	-0.322559000	-1.675592000	-2.606170000
6	3.174270000	-1.871159000	0.362068000
1	3.484540000	-2.827017000	0.788725000
6	4.108713000	-0.976450000	-0.171627000
1	5.173340000	-1.219569000	-0.153927000
6	-1.494820000	0.269911000	4.056792000
1	-2.344431000	0.115384000	4.724887000
6	-0.105500000	-2.701676000	-1.532557000
1	0.954518000	-2.998925000	-1.544220000
1	-0.680740000	-3.617577000	-1.735579000
6	-0.274428000	0.709063000	4.561727000
1	-0.144755000	0.887060000	5.631760000
6	-0.313852000	-1.997222000	-3.965965000
1	-0.191563000	-3.038226000	-4.272605000
6	-0.624978000	0.599964000	-3.097322000
1	-0.753029000	1.597368000	-2.675313000
6	-3.617165000	0.234483000	0.984953000
1	-4.681760000	-0.016810000	0.864776000
1	-3.527922000	1.336575000	0.986425000
6	-1.732669000	-2.742053000	0.323804000
1	-1.800658000	-2.518197000	1.393130000
1	-1.731536000	-3.841162000	0.219884000
6	-2.898260000	-2.146160000	-0.455147000
1	-3.866446000	-2.413968000	-0.009475000
1	-2.915428000	-2.484945000	-1.500715000
16	-2.807839000	-0.295779000	-0.555376000
6	-3.072177000	-0.379006000	2.262908000
1	-3.172917000	-1.475582000	2.244733000
1	-3.749484000	-0.062985000	3.071414000
8	-0.519688000	1.582743000	-0.452827000
8	3.338199000	1.997029000	1.479682000

6	3.222043000	3.348161000	1.253250000
6	3.919123000	3.965388000	2.534529000
1	3.848746000	5.060225000	2.438017000
1	3.396329000	3.644046000	3.445445000
1	4.973251000	3.661751000	2.581291000
6	4.028983000	3.785878000	0.008215000
1	5.064207000	3.418572000	0.066775000
1	3.560815000	3.381919000	-0.903197000
1	4.043153000	4.883521000	-0.074947000
6	1.758336000	3.820694000	1.195652000
1	1.219440000	3.314107000	0.378809000
1	1.243403000	3.584048000	2.138265000
1	1.694373000	4.907710000	1.030085000
16	-3.303391000	3.822230000	1.352286000
6	-1.713443000	3.598437000	2.223804000
1	-1.936691000	3.291084000	3.254304000
1	-1.128444000	2.814865000	1.722869000
1	-1.140124000	4.535978000	2.240795000
6	-2.659906000	4.090412000	-0.338751000
1	-3.524514000	4.159902000	-1.013389000
1	-2.083359000	5.025455000	-0.392176000
1	-2.022097000	3.242287000	-0.631000000

^{2}TS$_{SO}$:

26	-0.648333000	-0.053754000	-0.278790000
7	-0.673900000	0.226189000	1.767257000
6	0.502898000	0.740012000	2.228615000
1	1.221892000	1.089856000	1.493410000
7	-0.598937000	-0.438202000	-2.213957000
6	0.814416000	0.864676000	3.575052000
1	1.788870000	1.267345000	3.855570000
6	0.723092000	-2.351831000	0.761798000
1	0.420903000	-2.081447000	1.784721000
1	1.051973000	-3.402631000	0.773488000
6	-0.556965000	-1.079432000	-4.916238000
1	-0.543435000	-1.335176000	-5.978120000
7	1.325186000	-0.280617000	-0.296624000
6	-0.759540000	0.243724000	-4.503506000
1	-0.906524000	1.050311000	-5.223727000
6	1.801451000	-1.428590000	0.258083000
6	3.545790000	0.377919000	-0.892202000
1	4.200201000	1.103277000	-1.377175000
7	-0.456345000	-2.155761000	-0.139827000
6	-1.649431000	-0.047216000	2.684710000
6	2.168001000	0.599839000	-0.873279000
1	1.700641000	1.482334000	-1.310120000
6	-0.395288000	-1.720126000	-2.599222000
6	3.169216000	-1.699182000	0.282948000
1	3.529060000	-2.628333000	0.729160000
6	4.053663000	-0.780102000	-0.293746000
1	5.127976000	-0.976437000	-0.291011000
6	-1.374464000	0.047486000	4.061126000
1	-2.171092000	-0.203032000	4.764998000
6	-0.149684000	-2.716586000	-1.503892000
1	0.907772000	-3.021188000	-1.535053000

1	-0.733016000	-3.634900000	-1.669267000
6	-0.131811000	0.469169000	4.523445000
1	0.075782000	0.525839000	5.594479000
6	-0.374819000	-2.072225000	-3.951261000
1	-0.213994000	-3.114821000	-4.233662000
6	-0.779682000	0.531042000	-3.142820000
1	-0.940193000	1.533332000	-2.743799000
6	-3.644582000	0.199070000	1.052945000
1	-4.717011000	-0.032430000	0.969545000
1	-3.530834000	1.298471000	1.047187000
6	-1.708615000	-2.744864000	0.412682000
1	-1.745114000	-2.486361000	1.476215000
1	-1.678040000	-3.846491000	0.346181000
6	-2.918979000	-2.210776000	-0.344188000
1	-3.863060000	-2.497299000	0.139939000
1	-2.959311000	-2.580063000	-1.378729000
16	-2.892825000	-0.360747000	-0.506127000
6	-3.074054000	-0.407585000	2.320771000
1	-3.225941000	-1.498897000	2.341646000
1	-3.704090000	-0.037358000	3.145607000
8	-0.677178000	1.583851000	-0.524687000
8	3.207073000	2.059230000	1.389115000
6	3.292773000	3.420386000	1.212503000
6	3.053038000	3.951362000	2.688623000
1	3.144519000	5.047902000	2.646778000
1	2.046726000	3.679240000	3.032815000
1	3.809219000	3.543559000	3.372530000
6	4.691188000	3.860612000	0.738905000
1	5.469469000	3.467648000	1.410411000
1	4.880551000	3.476563000	-0.276146000
1	4.769912000	4.958725000	0.709289000
6	2.163875000	3.973994000	0.316339000
1	2.302708000	3.632244000	-0.720769000
1	1.185477000	3.622820000	0.674940000
1	2.168661000	5.074680000	0.316886000
16	-3.113824000	3.728075000	1.654429000
6	-1.365816000	3.540679000	2.154654000
1	-1.344342000	3.075102000	3.149290000
1	-0.862948000	2.890136000	1.424853000
1	-0.858447000	4.515069000	2.196508000
6	-2.844391000	4.064580000	-0.124697000
1	-3.832311000	4.098364000	-0.605194000
1	-2.338416000	5.029116000	-0.276230000
1	-2.247225000	3.248973000	-0.560125000

$^2PC_{DMS}$:

26	-0.776996000	-0.194266000	-0.200188000
7	-0.995850000	0.361713000	1.758516000
6	0.056471000	1.114754000	2.204513000
1	0.835296000	1.372858000	1.490628000
7	-0.580501000	-0.636190000	-2.101056000
6	0.172925000	1.602511000	3.500094000
1	1.049389000	2.198868000	3.756552000
6	0.628729000	-2.232999000	1.093074000
1	0.218528000	-1.905706000	2.059893000

1	1.010464000	-3.259888000	1.210741000
6	-0.415083000	-1.484301000	-4.755895000
1	-0.356003000	-1.819690000	-5.793920000
7	1.178488000	-0.204635000	-0.059467000
6	-0.714137000	-0.151111000	-4.449839000
1	-0.892245000	0.587343000	-5.233861000
6	1.690068000	-1.286529000	0.597840000
6	3.400827000	0.513585000	-0.594582000
1	4.043863000	1.243133000	-1.088517000
7	-0.478176000	-2.175761000	0.080142000
6	-2.027685000	0.159869000	2.642618000
6	2.015090000	0.683535000	-0.639099000
1	1.538475000	1.528238000	-1.135235000
6	-0.280793000	-1.926961000	-2.392137000
6	3.062222000	-1.493339000	0.702415000
1	3.440070000	-2.372494000	1.228513000
6	3.933776000	-0.578524000	0.092931000
1	5.014476000	-0.728522000	0.147417000
6	-1.941367000	0.612811000	3.969394000
1	-2.779843000	0.412992000	4.640160000
6	0.005629000	-2.801159000	-1.209475000
1	1.094210000	-2.947840000	-1.136937000
1	-0.434688000	-3.802122000	-1.330169000
6	-0.832237000	1.321730000	4.424876000
1	-0.774015000	1.668489000	5.459402000
6	-0.196257000	-2.383965000	-3.708731000
1	0.040398000	-3.432641000	-3.901830000
6	-0.794068000	0.237174000	-3.114982000
1	-1.025973000	1.255703000	-2.807440000
6	-3.925782000	-0.077187000	0.921921000
1	-4.940556000	-0.482330000	0.798180000
1	-3.992253000	1.018301000	0.841465000
6	-1.715436000	-2.860430000	0.554596000
1	-1.852496000	-2.585619000	1.605278000
1	-1.581645000	-3.955445000	0.517559000
6	-2.901895000	-2.447725000	-0.306909000
1	-3.857602000	-2.780546000	0.121784000
1	-2.835124000	-2.859673000	-1.324065000
16	-2.966528000	-0.605531000	-0.541255000
6	-3.332768000	-0.501894000	2.255858000
1	-3.264487000	-1.598624000	2.310954000
1	-4.068079000	-0.238051000	3.031603000
8	-0.771063000	1.704352000	-0.706987000
8	2.182271000	2.976318000	0.985011000
6	3.267134000	3.532320000	1.643792000
6	4.276467000	4.128267000	0.631998000
1	5.113176000	4.632365000	1.143957000
1	4.694777000	3.328814000	-0.000045000
1	3.772827000	4.858357000	-0.021032000
6	2.703187000	4.696347000	2.522262000
1	2.230703000	5.459297000	1.885133000
1	1.953452000	4.316428000	3.232585000
1	3.525210000	5.162847000	3.089678000
6	3.963802000	2.495251000	2.554708000
1	3.258423000	2.092376000	3.298581000

1	4.331633000	1.653130000	1.948024000
1	4.819896000	2.935287000	3.092891000
16	-1.647956000	2.808872000	0.044363000
6	-0.533442000	4.235478000	0.070731000
1	0.381246000	3.904554000	0.592417000
1	-0.300405000	4.530643000	-0.960854000
1	-1.040083000	5.045511000	0.614168000
6	-2.820218000	3.348900000	-1.238194000
1	-3.490395000	2.505075000	-1.446964000
1	-3.395213000	4.194032000	-0.834561000
1	-2.265090000	3.641617000	-2.138731000

^{4}I1$_{DMS}$:

26	-0.776996000	-0.194266000	-0.200188000
7	-0.995850000	0.361713000	1.758516000
6	0.056471000	1.114754000	2.204513000
1	0.835296000	1.372858000	1.490628000
7	-0.580501000	-0.636190000	-2.101056000
6	0.172925000	1.602511000	3.500094000
1	1.049389000	2.198868000	3.756552000
6	0.628729000	-2.232999000	1.093074000
1	0.218528000	-1.905706000	2.059893000
1	1.010464000	-3.259888000	1.210741000
6	-0.415083000	-1.484301000	-4.755895000
1	-0.356003000	-1.819690000	-5.793920000
7	1.178488000	-0.204635000	-0.059467000
6	-0.714137000	-0.151111000	-4.449839000
1	-0.892245000	0.587343000	-5.233861000
6	1.690068000	-1.286529000	0.597840000
6	3.400827000	0.513585000	-0.594582000
1	4.043863000	1.243133000	-1.088517000
7	-0.478176000	-2.175761000	0.080142000
6	-2.027685000	0.159869000	2.642618000
6	2.015090000	0.683535000	-0.639099000
1	1.538475000	1.528238000	-1.135235000
6	-0.280793000	-1.926961000	-2.392137000
6	3.062222000	-1.493339000	0.702415000
1	3.440070000	-2.372494000	1.228513000
6	3.933776000	-0.578524000	0.092931000
1	5.014476000	-0.728522000	0.147417000
6	-1.941367000	0.612811000	3.969394000
1	-2.779843000	0.412992000	4.640160000
6	0.005629000	-2.801159000	-1.209475000
1	1.094210000	-2.947840000	-1.136937000
1	-0.434688000	-3.802122000	-1.330169000
6	-0.832237000	1.321730000	4.424876000
1	-0.774015000	1.668489000	5.459402000
6	-0.196257000	-2.383965000	-3.708731000
1	0.040398000	-3.432641000	-3.901830000
6	-0.794068000	0.237174000	-3.114982000
1	-1.025973000	1.255703000	-2.807440000
6	-3.925782000	-0.077187000	0.921921000
1	-4.940556000	-0.482330000	0.798180000
1	-3.992253000	1.018301000	0.841465000
6	-1.715436000	-2.860430000	0.554596000

1	-1.852496000	-2.585619000	1.605278000
1	-1.581645000	-3.955445000	0.517559000
6	-2.901895000	-2.447725000	-0.306909000
1	-3.857602000	-2.780546000	0.121784000
1	-2.835124000	-2.859673000	-1.324065000
16	-2.966528000	-0.605531000	-0.541255000
6	-3.332768000	-0.501894000	2.255858000
1	-3.264487000	-1.598624000	2.310954000
1	-4.068079000	-0.238051000	3.031603000
8	-0.771063000	1.704352000	-0.706987000
8	2.182271000	2.976318000	0.985011000
6	3.267134000	3.532320000	1.643792000
6	4.276467000	4.128267000	0.631998000
1	5.113176000	4.632365000	1.143957000
1	4.694777000	3.328814000	-0.000045000
1	3.772827000	4.858357000	-0.021032000
6	2.703187000	4.696347000	2.522262000
1	2.230703000	5.459297000	1.885133000
1	1.953452000	4.316428000	3.232585000
1	3.525210000	5.162847000	3.089678000
6	3.963802000	2.495251000	2.554708000
1	3.258423000	2.092376000	3.298581000
1	4.331633000	1.653130000	1.948024000
1	4.819896000	2.935287000	3.092891000
16	-1.647956000	2.808872000	0.044363000
6	-0.533442000	4.235478000	0.070731000
1	0.381246000	3.904554000	0.592417000
1	-0.300405000	4.530643000	-0.960854000
1	-1.040083000	5.045511000	0.614168000
6	-2.820218000	3.348900000	-1.238194000
1	-3.490395000	2.505075000	-1.446964000
1	-3.395213000	4.194032000	-0.834561000
1	-2.265090000	3.641617000	-2.138731000

$^4PC_{DMS}$:

26	-0.764421000	-0.101170000	-0.144773000
7	-0.972529000	0.386914000	1.934648000
6	0.008431000	1.239070000	2.327896000
1	0.834515000	1.432679000	1.636638000
7	-0.618041000	-0.572683000	-2.190375000
6	-0.003883000	1.914133000	3.543845000
1	0.818920000	2.591188000	3.778428000
6	0.745621000	-2.417120000	0.928076000
1	0.329274000	-2.144694000	1.910994000
1	1.161111000	-3.436279000	1.005483000
6	-0.629465000	-1.348461000	-4.868307000
1	-0.639209000	-1.658328000	-5.916090000
7	1.320173000	-0.261968000	-0.001411000
6	-0.939282000	-0.030527000	-4.509355000
1	-1.193937000	0.720373000	-5.259830000
6	1.804785000	-1.426720000	0.506891000
6	3.551130000	0.439351000	-0.524326000
1	4.205575000	1.193651000	-0.963962000
7	-0.356995000	-2.341776000	-0.070586000
6	-2.025257000	0.179444000	2.781342000

6	2.171047000	0.653694000	-0.504916000
1	1.722656000	1.567635000	-0.895804000
6	-0.306839000	-1.844356000	-2.529577000
6	3.172575000	-1.688643000	0.547871000
1	3.532690000	-2.633890000	0.960019000
6	4.061312000	-0.739129000	0.022611000
1	5.137039000	-0.930755000	0.027496000
6	-2.088779000	0.839859000	4.018434000
1	-2.947562000	0.659177000	4.668228000
6	0.110191000	-2.777325000	-1.418099000
1	1.211224000	-2.821215000	-1.411958000
1	-0.234615000	-3.803023000	-1.627305000
6	-1.076697000	1.716288000	4.412900000
1	-1.132285000	2.233277000	5.374171000
6	-0.312352000	-2.266073000	-3.863755000
1	-0.067466000	-3.303801000	-4.102897000
6	-0.930819000	0.317681000	-3.160808000
1	-1.174829000	1.321212000	-2.810158000
6	-3.930174000	-0.508746000	1.167554000
1	-4.838022000	-1.129152000	1.142110000
1	-4.245554000	0.544069000	1.118113000
6	-1.569294000	-3.078758000	0.349281000
1	-1.702176000	-2.917115000	1.424697000
1	-1.434816000	-4.167025000	0.204700000
6	-2.797880000	-2.622350000	-0.435366000
1	-3.711515000	-3.098590000	-0.052503000
1	-2.717664000	-2.879568000	-1.501556000
16	-3.064938000	-0.793942000	-0.421999000
6	-3.137410000	-0.785566000	2.445431000
1	-2.756852000	-1.817218000	2.450734000
1	-3.861291000	-0.751621000	3.274271000
8	-0.907537000	1.834501000	-0.654702000
8	2.096589000	3.019507000	1.062822000
6	3.172480000	3.572710000	1.740408000
6	4.194020000	4.068575000	0.670504000
1	5.054717000	4.551271000	1.162546000
1	4.559642000	3.219625000	0.074476000
1	3.715083000	4.794504000	-0.004170000
6	2.704676000	4.802343000	2.564109000
1	2.241013000	5.552377000	1.904214000
1	1.957920000	4.498317000	3.315463000
1	3.551231000	5.272276000	3.092363000
6	3.854091000	2.533412000	2.658595000
1	3.147992000	2.181640000	3.428854000
1	4.173527000	1.659227000	2.069826000
1	4.736847000	2.950514000	3.172017000
16	-1.506920000	3.064502000	0.161486000
6	-0.353443000	4.397574000	-0.236511000
1	0.616420000	4.087645000	0.199705000
1	-0.301910000	4.513954000	-1.327648000
1	-0.725009000	5.313288000	0.245742000
6	-2.940476000	3.562140000	-0.836998000
1	-3.690253000	2.765502000	-0.757197000
1	-3.334304000	4.495020000	-0.409105000
1	-2.622650000	3.705811000	-1.877686000

$^4Re_{DMS}$:

26	-0.350413000	-0.519612000	-0.107981000
7	-0.296526000	-0.476820000	2.237662000
6	0.841067000	0.137069000	2.644144000
1	1.617109000	0.261206000	1.891054000
7	-0.698319000	-0.767808000	-2.227696000
6	1.048020000	0.606248000	3.939574000
1	1.996707000	1.080287000	4.198559000
6	1.411611000	-2.726663000	0.411556000
1	1.243905000	-2.625435000	1.495127000
1	1.831752000	-3.727699000	0.224752000
6	-1.668405000	-1.512611000	-4.714902000
1	-2.059986000	-1.810612000	-5.690516000
7	1.700493000	-0.453750000	-0.345436000
6	-1.794027000	-0.187717000	-4.275115000
1	-2.277352000	0.572684000	-4.891292000
6	2.327074000	-1.632498000	-0.070842000
6	3.793812000	0.522197000	-0.980065000
1	4.341000000	1.392124000	-1.347241000
7	0.086951000	-2.558462000	-0.269023000
6	-1.299168000	-0.638129000	3.144703000
6	2.413003000	0.599104000	-0.804556000
1	1.844502000	1.507482000	-1.003926000
6	-0.557636000	-2.044897000	-2.648876000
6	3.704932000	-1.773987000	-0.225924000
1	4.183647000	-2.729605000	-0.003126000
6	4.449069000	-0.677414000	-0.678887000
1	5.530344000	-0.765263000	-0.808010000
6	-1.164065000	-0.162257000	4.459654000
1	-1.992877000	-0.293213000	5.158923000
6	0.219218000	-2.946174000	-1.724297000
1	1.284461000	-2.861610000	-1.993299000
1	-0.057930000	-4.003328000	-1.852068000
6	0.014960000	0.463258000	4.868731000
1	0.121826000	0.831148000	5.892254000
6	-1.042363000	-2.457360000	-3.893202000
1	-0.933111000	-3.496777000	-4.210267000
6	-1.301499000	0.146104000	-3.014404000
1	-1.390990000	1.149176000	-2.590057000
6	-3.365950000	-0.855290000	1.587230000
1	-4.370331000	-1.303340000	1.583236000
1	-3.484842000	0.236911000	1.609192000
6	-0.966301000	-3.368718000	0.422541000
1	-0.856211000	-3.172960000	1.495718000
1	-0.771768000	-4.443542000	0.258141000
6	-2.361482000	-3.005772000	-0.062962000
1	-3.131461000	-3.488553000	0.555259000
1	-2.534552000	-3.327302000	-1.099900000
16	-2.675482000	-1.177405000	-0.080281000
6	-2.567122000	-1.374943000	2.780956000
1	-2.356709000	-2.449664000	2.658563000
1	-3.240582000	-1.322657000	3.650617000
8	-0.741795000	1.216444000	-0.170756000
8	0.195555000	2.065528000	0.560457000

6	-0.528951000	3.252657000	1.051789000
6	0.572349000	3.986917000	1.820512000
1	0.167443000	4.928034000	2.223669000
1	0.938892000	3.380208000	2.662150000
1	1.419281000	4.231843000	1.161222000
6	-1.030137000	4.063707000	-0.145417000
1	-0.192780000	4.339407000	-0.805566000
1	-1.762041000	3.485192000	-0.727945000
1	-1.517892000	4.988698000	0.200067000
6	-1.671106000	2.814289000	1.968685000
1	-2.388557000	2.198700000	1.407910000
1	-1.292713000	2.234263000	2.822774000
1	-2.202593000	3.696514000	2.357567000
16	-3.971872000	1.745474000	-1.059520000
6	-5.106557000	0.566049000	-1.872324000
1	-4.501473000	-0.041388000	-2.559248000
1	-5.576779000	-0.097581000	-1.131889000
1	-5.878426000	1.099890000	-2.445223000
6	-5.164860000	2.637581000	0.000764000
1	-4.603385000	3.413288000	0.538919000
1	-5.946357000	3.117277000	-0.606210000
1	-5.624913000	1.955131000	0.730382000

$^{4}TS_{direct}$:

26	-0.350413000	-0.519612000	-0.107981000
7	-0.296526000	-0.476820000	2.237662000
6	0.841067000	0.137069000	2.644144000
1	1.617109000	0.261206000	1.891054000
7	-0.698319000	-0.767808000	-2.227696000
6	1.048020000	0.606248000	3.939574000
1	1.996707000	1.080287000	4.198559000
6	1.411611000	-2.726663000	0.411556000
1	1.243905000	-2.625435000	1.495127000
1	1.831752000	-3.727699000	0.224752000
6	-1.668405000	-1.512611000	-4.714902000
1	-2.059986000	-1.810612000	-5.690516000
7	1.700493000	-0.453750000	-0.345436000
6	-1.794027000	-0.187717000	-4.275115000
1	-2.277352000	0.572684000	-4.891292000
6	2.327074000	-1.632498000	-0.070842000
6	3.793812000	0.522197000	-0.980065000
1	4.341000000	1.392124000	-1.347241000
7	0.086951000	-2.558462000	-0.269023000
6	-1.299168000	-0.638129000	3.144703000
6	2.413003000	0.599104000	-0.804556000
1	1.844502000	1.507482000	-1.003926000
6	-0.557636000	-2.044897000	-2.648876000
6	3.704932000	-1.773987000	-0.225924000
1	4.183647000	-2.729605000	-0.003126000
6	4.449069000	-0.677414000	-0.678887000
1	5.530344000	-0.765263000	-0.808010000
6	-1.164065000	-0.162257000	4.459654000
1	-1.992877000	-0.293213000	5.158923000
6	0.219218000	-2.946174000	-1.724297000
1	1.284461000	-2.861610000	-1.993299000

1	-0.057930000	-4.003328000	-1.852068000
6	0.014960000	0.463258000	4.868731000
1	0.121826000	0.831148000	5.892254000
6	-1.042363000	-2.457360000	-3.893202000
1	-0.933111000	-3.496777000	-4.210267000
6	-1.301499000	0.146104000	-3.014404000
1	-1.390990000	1.149176000	-2.590057000
6	-3.365950000	-0.855290000	1.587230000
1	-4.370331000	-1.303340000	1.583236000
1	-3.484842000	0.236911000	1.609192000
6	-0.966301000	-3.368718000	0.422541000
1	-0.856211000	-3.172960000	1.495718000
1	-0.771768000	-4.443542000	0.258141000
6	-2.361482000	-3.005772000	-0.062962000
1	-3.131461000	-3.488553000	0.555259000
1	-2.534552000	-3.327302000	-1.099900000
16	-2.675482000	-1.177405000	-0.080281000
6	-2.567122000	-1.374943000	2.780956000
1	-2.356709000	-2.449664000	2.658563000
1	-3.240582000	-1.322657000	3.650617000
8	-0.741795000	1.216444000	-0.170756000
8	0.195555000	2.065528000	0.560457000
6	-0.528951000	3.252657000	1.051789000
6	0.572349000	3.986917000	1.820512000
1	0.167443000	4.928034000	2.223669000
1	0.938892000	3.380208000	2.662150000
1	1.419281000	4.231843000	1.161222000
6	-1.030137000	4.063707000	-0.145417000
1	-0.192780000	4.339407000	-0.805566000
1	-1.762041000	3.485192000	-0.727945000
1	-1.517892000	4.988698000	0.200067000
6	-1.671106000	2.814289000	1.968685000
1	-2.388557000	2.198700000	1.407910000
1	-1.292713000	2.234263000	2.822774000
1	-2.202593000	3.696514000	2.357567000
16	-3.971872000	1.745474000	-1.059520000
6	-5.106557000	0.566049000	-1.872324000
1	-4.501473000	-0.041388000	-2.559248000
1	-5.576779000	-0.097581000	-1.131889000
1	-5.878426000	1.099890000	-2.445223000
6	-5.164860000	2.637581000	0.000764000
1	-4.603385000	3.413288000	0.538919000
1	-5.946357000	3.117277000	-0.606210000
1	-5.624913000	1.955131000	0.730382000

^{4}TS$_{SO}$:

26	-0.875912000	-0.012614000	-0.230587000
7	-1.111898000	0.465798000	1.767854000
6	-0.148295000	1.355111000	2.145653000
1	0.714515000	1.462294000	1.488535000
7	-0.719948000	-0.593388000	-2.190706000
6	-0.234738000	2.143208000	3.287129000
1	0.572888000	2.840125000	3.509974000
6	1.028731000	-1.947218000	0.939604000
1	0.676953000	-1.593574000	1.921013000

1	1.503494000	-2.931720000	1.081491000
6	-0.725778000	-1.560562000	-4.802614000
1	-0.733557000	-1.945585000	-5.825039000
7	1.370962000	0.136056000	-0.203709000
6	-1.144703000	-0.252406000	-4.528343000
1	-1.484454000	0.415971000	-5.321688000
6	1.981386000	-0.952884000	0.321910000
6	3.484724000	0.928151000	-1.015885000
1	4.039487000	1.685607000	-1.572013000
7	-0.169173000	-2.060064000	0.043058000
6	-2.209300000	0.321571000	2.575836000
6	2.101841000	1.065607000	-0.846797000
1	1.547974000	1.911141000	-1.256723000
6	-0.294747000	-1.854943000	-2.448319000
6	3.360071000	-1.142978000	0.222440000
1	3.824661000	-2.034332000	0.650124000
6	4.122222000	-0.185510000	-0.463947000
1	5.201238000	-0.318721000	-0.573168000
6	-2.347618000	1.116370000	3.726199000
1	-3.241600000	0.991926000	4.340289000
6	0.244481000	-2.635458000	-1.283541000
1	1.344040000	-2.619177000	-1.344833000
1	-0.047240000	-3.695509000	-1.342628000
6	-1.369477000	2.040274000	4.091109000
1	-1.492110000	2.658145000	4.983858000
6	-0.298122000	-2.370989000	-3.747739000
1	0.038219000	-3.395451000	-3.922675000
6	-1.136161000	0.192010000	-3.209093000
1	-1.469571000	1.189683000	-2.915388000
6	-4.067537000	-0.682766000	1.033689000
1	-4.943489000	-1.343018000	1.115707000
1	-4.432285000	0.332553000	0.817405000
6	-1.224163000	-2.895370000	0.680055000
1	-1.343201000	-2.528949000	1.705953000
1	-0.876496000	-3.942605000	0.747855000
6	-2.545195000	-2.858601000	-0.087212000
1	-3.323873000	-3.396981000	0.472145000
1	-2.457564000	-3.352775000	-1.065760000
16	-3.161939000	-1.164378000	-0.479611000
6	-3.275995000	-0.723311000	2.340534000
1	-2.853564000	-1.727935000	2.495137000
1	-4.012949000	-0.610684000	3.150520000
8	-1.283258000	1.532132000	-0.747457000
8	2.294348000	3.059215000	1.027966000
6	3.387095000	3.212768000	1.863343000
6	4.677080000	3.534151000	1.073418000
1	5.506292000	3.787335000	1.753779000
1	4.982279000	2.663158000	0.474368000
1	4.507623000	4.383584000	0.393635000
6	3.038048000	4.460338000	2.750425000
1	2.905085000	5.350833000	2.119317000
1	2.117031000	4.289860000	3.325633000
1	3.869333000	4.633658000	3.452432000
6	3.586626000	1.975239000	2.766991000
1	2.696370000	1.788571000	3.386623000

1	3.762814000	1.084520000	2.144081000
1	4.453103000	2.104395000	3.435749000
16	-1.949006000	3.230152000	0.470610000
6	-0.543145000	4.228700000	-0.078181000
1	0.338393000	3.910880000	0.498012000
1	-0.378752000	4.048427000	-1.148846000
1	-0.763343000	5.289790000	0.107877000
6	-3.186772000	3.639590000	-0.785185000
1	-4.072215000	3.025008000	-0.581516000
1	-3.441563000	4.705968000	-0.705801000
1	-2.779536000	3.408927000	-1.778076000

^{6}I1$_{DMS}$:

26	-0.496053000	-0.016571000	-0.277037000
7	-0.691695000	0.206599000	1.901409000
6	0.470255000	0.717578000	2.388625000
1	1.292713000	0.854459000	1.688626000
7	-0.564601000	-0.394302000	-2.329664000
6	0.652698000	1.091369000	3.714577000
1	1.623763000	1.478538000	4.027242000
6	0.812831000	-2.417872000	0.678232000
1	0.505477000	-2.172920000	1.706482000
1	1.080480000	-3.485756000	0.651179000
6	-0.909513000	-1.105412000	-4.985021000
1	-1.052855000	-1.389143000	-6.030268000
7	1.571264000	-0.332946000	-0.248925000
6	-1.012901000	0.236488000	-4.593211000
1	-1.232255000	1.024870000	-5.315228000
6	1.956820000	-1.538383000	0.245566000
6	3.850192000	0.226872000	-0.715218000
1	4.570012000	0.938903000	-1.119881000
7	-0.345497000	-2.132054000	-0.230761000
6	-1.755859000	0.098232000	2.750432000
6	2.488695000	0.533442000	-0.725749000
1	2.093893000	1.468419000	-1.126421000
6	-0.444361000	-1.691201000	-2.698486000
6	3.303193000	-1.898680000	0.302366000
1	3.591811000	-2.874203000	0.698753000
6	4.261425000	-1.000370000	-0.182854000
1	5.321009000	-1.264674000	-0.158916000
6	-1.631503000	0.487084000	4.095594000
1	-2.502190000	0.397730000	4.748391000
6	-0.024487000	-2.642833000	-1.615194000
1	1.067137000	-2.771850000	-1.686269000
1	-0.466099000	-3.641084000	-1.755523000
6	-0.424492000	0.973856000	4.594893000
1	-0.334710000	1.267038000	5.643625000
6	-0.623609000	-2.083329000	-4.027377000
1	-0.535743000	-3.136104000	-4.303686000
6	-0.843704000	0.555900000	-3.248797000
1	-0.932061000	1.572056000	-2.859604000
6	-3.707320000	0.080709000	1.033764000
1	-4.755694000	-0.242900000	0.954781000
1	-3.685520000	1.184602000	1.021852000
6	-1.598824000	-2.752247000	0.292834000

1	-1.647029000	-2.515494000	1.361089000
1	-1.534266000	-3.851250000	0.204664000
6	-2.828536000	-2.245371000	-0.449171000
1	-3.751002000	-2.609622000	0.024993000
1	-2.848548000	-2.582848000	-1.495254000
16	-2.915657000	-0.399394000	-0.542750000
6	-3.090671000	-0.464073000	2.316600000
1	-3.056092000	-1.565428000	2.297284000
1	-3.808727000	-0.234518000	3.119227000
8	-0.489172000	1.620131000	-0.484459000
8	3.365584000	1.875362000	1.601649000
6	3.356585000	3.241925000	1.442693000
6	3.958864000	3.753857000	2.813088000
1	3.964258000	4.854254000	2.767395000
1	3.330766000	3.428657000	3.653400000
1	4.982908000	3.381052000	2.947347000
6	4.310035000	3.683201000	0.305850000
1	5.304446000	3.231719000	0.435669000
1	3.901375000	3.370375000	-0.667912000
1	4.412271000	4.779281000	0.295203000
6	1.939312000	3.814186000	1.262800000
1	1.457588000	3.377093000	0.373996000
1	1.314730000	3.579233000	2.137062000
1	1.963026000	4.908291000	1.139034000
16	-3.291619000	3.667041000	1.398004000
6	-1.545241000	3.648497000	1.924801000
1	-1.521726000	3.403106000	2.994904000
1	-1.001089000	2.884623000	1.351047000
1	-1.080781000	4.632307000	1.766528000
6	-3.039823000	3.880164000	-0.400663000
1	-4.022827000	3.801126000	-0.884712000
1	-2.605668000	4.866472000	-0.620007000
1	-2.373673000	3.089460000	-0.777106000

$^6PC_{DMS}$:

26	-0.746441000	-0.043792000	-0.190882000
7	-0.993164000	0.413749000	1.959282000
6	-0.080196000	1.370758000	2.277919000
1	0.810826000	1.446418000	1.650546000
7	-0.726726000	-0.613544000	-2.271034000
6	-0.248089000	2.273095000	3.325072000
1	0.520237000	3.024138000	3.509198000
6	0.983850000	-2.173760000	0.873184000
1	0.593469000	-1.887050000	1.862987000
1	1.467182000	-3.160690000	0.966974000
6	-0.934627000	-1.589222000	-4.872089000
1	-1.021469000	-1.977930000	-5.889675000
7	1.360681000	-0.009934000	-0.122520000
6	-1.311402000	-0.272659000	-4.576796000
1	-1.695058000	0.396820000	-5.349185000
6	1.954314000	-1.132527000	0.366429000
6	3.504526000	0.814089000	-0.825109000
1	4.074886000	1.593513000	-1.331771000
7	-0.174780000	-2.212121000	-0.068352000
6	-2.110265000	0.310828000	2.738143000

6	2.118626000	0.952959000	-0.691896000
1	1.580934000	1.815775000	-1.083830000
6	-0.349942000	-1.881307000	-2.552049000
6	3.333996000	-1.311822000	0.307344000
1	3.782929000	-2.226468000	0.701042000
6	4.122009000	-0.323144000	-0.304265000
1	5.203634000	-0.455228000	-0.384477000
6	-2.338503000	1.202821000	3.797267000
1	-3.250420000	1.099100000	4.389504000
6	0.252586000	-2.680885000	-1.420648000
1	1.347868000	-2.583486000	-1.491776000
1	0.031825000	-3.754145000	-1.538982000
6	-1.409613000	2.201373000	4.095671000
1	-1.588107000	2.900658000	4.916342000
6	-0.450365000	-2.405284000	-3.845246000
1	-0.150327000	-3.437913000	-4.039148000
6	-1.200915000	0.174732000	-3.261618000
1	-1.492943000	1.181900000	-2.958376000
6	-3.947201000	-0.690540000	1.227930000
1	-4.774149000	-1.415061000	1.254845000
1	-4.385615000	0.313981000	1.127682000
6	-1.299761000	-3.020521000	0.458343000
1	-1.399107000	-2.788807000	1.525317000
1	-1.068026000	-4.099254000	0.382672000
6	-2.602298000	-2.738736000	-0.289339000
1	-3.437793000	-3.288657000	0.166496000
1	-2.543297000	-3.053801000	-1.341193000
16	-3.070604000	-0.952179000	-0.361529000
6	-3.105429000	-0.798113000	2.504113000
1	-2.601377000	-1.774015000	2.552096000
1	-3.812915000	-0.796098000	3.348133000
8	-1.024853000	1.850830000	-0.732399000
8	2.266694000	3.059016000	0.999179000
6	3.343833000	3.286738000	1.844953000
6	4.645182000	3.572616000	1.058338000
1	5.458461000	3.889609000	1.731975000
1	4.979668000	2.668070000	0.528412000
1	4.475017000	4.368669000	0.316246000
6	2.980118000	4.573284000	2.658390000
1	2.847712000	5.427871000	1.978155000
1	2.050647000	4.429752000	3.228490000
1	3.795745000	4.798639000	3.364447000
6	3.549204000	2.101874000	2.817319000
1	2.658311000	1.946480000	3.444692000
1	3.729791000	1.178390000	2.244629000
1	4.413401000	2.271080000	3.480713000
16	-1.713507000	2.989272000	0.149213000
6	-0.505834000	4.335393000	0.078916000
1	0.419604000	3.955602000	0.544024000
1	-0.335131000	4.616707000	-0.968485000
1	-0.915403000	5.175380000	0.658001000
6	-2.996569000	3.623665000	-0.963802000
1	-3.741899000	2.825693000	-1.072096000
1	-3.450140000	4.503418000	-0.485802000
1	-2.544338000	3.882286000	-1.929998000

$^{6}Re_{DMS}$:

26	-0.413149000	-0.463719000	-0.099112000
7	-0.324212000	-0.456528000	2.139207000
6	0.798573000	0.204634000	2.524115000
1	1.555543000	0.357306000	1.757990000
7	-0.712603000	-0.790035000	-2.171543000
6	1.017232000	0.680492000	3.814166000
1	1.952573000	1.192037000	4.048553000
6	1.397402000	-2.815155000	0.416698000
1	1.238540000	-2.697094000	1.501005000
1	1.837665000	-3.813201000	0.251675000
6	-1.552568000	-1.474426000	-4.726339000
1	-1.893246000	-1.750614000	-5.727160000
7	1.708687000	-0.547150000	-0.363667000
6	-1.700131000	-0.159650000	-4.263630000
1	-2.147915000	0.617193000	-4.885959000
6	2.317078000	-1.726201000	-0.080458000
6	3.814314000	0.405055000	-1.000318000
1	4.372475000	1.266377000	-1.371597000
7	0.079047000	-2.673347000	-0.253896000
6	-1.299412000	-0.658844000	3.072952000
6	2.434878000	0.496530000	-0.821212000
1	1.878645000	1.413451000	-1.025067000
6	-0.543620000	-2.056879000	-2.616474000
6	3.694373000	-1.886737000	-0.237120000
1	4.161060000	-2.847528000	-0.009541000
6	4.453125000	-0.803570000	-0.696876000
1	5.532925000	-0.907037000	-0.827827000
6	-1.147422000	-0.177004000	4.382955000
1	-1.951382000	-0.343569000	5.103147000
6	0.195494000	-3.010354000	-1.706370000
1	1.261316000	-2.962253000	-1.984571000
1	-0.122809000	-4.049631000	-1.887903000
6	0.014683000	0.494722000	4.767046000
1	0.130669000	0.863674000	5.789092000
6	-0.966459000	-2.434060000	-3.895086000
1	-0.835454000	-3.465494000	-4.230072000
6	-1.274036000	0.143663000	-2.973431000
1	-1.382590000	1.140280000	-2.539652000
6	-3.407085000	-1.005754000	1.581871000
1	-4.386547000	-1.503723000	1.626837000
1	-3.580396000	0.080124000	1.579340000
6	-0.960032000	-3.483471000	0.426838000
1	-0.860759000	-3.290869000	1.502080000
1	-0.773659000	-4.562705000	0.269417000
6	-2.363340000	-3.144924000	-0.066519000
1	-3.125594000	-3.649146000	0.544569000
1	-2.519213000	-3.472596000	-1.104453000
16	-2.750213000	-1.335120000	-0.096021000
6	-2.545481000	-1.455098000	2.759538000
1	-2.291262000	-2.522182000	2.656280000
1	-3.191707000	-1.411405000	3.650026000
8	-0.649822000	1.394354000	-0.070118000
8	0.206684000	2.284253000	0.624366000

6	-0.578099000	3.456333000	1.086822000
6	0.489081000	4.270471000	1.818715000
1	0.034799000	5.193084000	2.211368000
1	0.905682000	3.704637000	2.665779000
1	1.307775000	4.553284000	1.139336000
6	-1.139602000	4.189462000	-0.130502000
1	-0.328135000	4.505574000	-0.804481000
1	-1.827729000	3.538247000	-0.689835000
1	-1.693073000	5.085500000	0.190351000
6	-1.675336000	2.961482000	2.029022000
1	-2.348399000	2.275253000	1.495982000
1	-1.245024000	2.432717000	2.892564000
1	-2.266618000	3.811513000	2.402504000
16	-3.917971000	1.640763000	-0.995399000
6	-5.156618000	0.559419000	-1.793285000
1	-4.608098000	-0.133044000	-2.445874000
1	-5.706875000	-0.022910000	-1.039374000
1	-5.858436000	1.150514000	-2.399110000
6	-5.027977000	2.651286000	0.049186000
1	-4.402980000	3.387456000	0.572251000
1	-5.766792000	3.182577000	-0.567877000
1	-5.542095000	2.022581000	0.790854000

^{6}TS$_{direct}$:

26	-1.075876000	-0.374793000	-0.138823000
7	-0.661958000	-0.348329000	2.127302000
6	0.576406000	0.161730000	2.361551000
1	1.179587000	0.372215000	1.484721000
7	-0.940787000	-0.888774000	-2.277154000
6	1.084887000	0.422944000	3.630147000
1	2.098170000	0.816095000	3.732266000
6	1.092487000	-2.530052000	0.226450000
1	1.181487000	-2.338587000	1.308676000
1	1.587719000	-3.494454000	0.021960000
6	-0.015876000	-1.500932000	-4.840771000
1	0.352412000	-1.744078000	-5.840310000
7	1.060504000	-0.255764000	-0.599374000
6	-0.472720000	-0.212700000	-4.540879000
1	-0.475225000	0.583006000	-5.287957000
6	1.766867000	-1.409734000	-0.522246000
6	2.873385000	0.784085000	-1.776865000
1	3.274530000	1.672976000	-2.267314000
7	-0.354545000	-2.588173000	-0.102929000
6	-1.458606000	-0.584301000	3.204613000
6	1.595812000	0.818794000	-1.217596000
1	0.973282000	1.715077000	-1.230067000
6	-0.488573000	-2.132870000	-2.561336000
6	3.047635000	-1.517618000	-1.065745000
1	3.591090000	-2.462393000	-0.996125000
6	3.609633000	-0.403545000	-1.701216000
1	4.608868000	-0.466492000	-2.138882000
6	-1.015514000	-0.311219000	4.510844000
1	-1.691685000	-0.502937000	5.347087000
6	-0.562644000	-3.161004000	-1.461874000
1	0.153600000	-3.978359000	-1.648662000

1	-1.564394000	-3.612354000	-1.503584000
6	0.267026000	0.185315000	4.736992000
1	0.614835000	0.387952000	5.752967000
6	-0.018705000	-2.470517000	-3.833943000
1	0.348613000	-3.481574000	-4.023655000
6	-0.913280000	0.053416000	-3.246455000
1	-1.246574000	1.053441000	-2.959637000
6	-3.678528000	-0.872770000	1.858354000
1	-4.714080000	-1.197672000	2.027774000
1	-3.689782000	0.206240000	1.658112000
6	-1.086397000	-3.371976000	0.927841000
1	-0.816331000	-2.943101000	1.901108000
1	-0.756928000	-4.427455000	0.924908000
6	-2.597853000	-3.316801000	0.718599000
1	-3.135103000	-3.646311000	1.617988000
1	-2.941238000	-3.965506000	-0.097945000
16	-3.174516000	-1.629418000	0.253874000
6	-2.829265000	-1.206976000	3.072992000
1	-2.736278000	-2.301092000	3.186881000
1	-3.415903000	-0.896326000	3.952502000
8	-1.482987000	1.512722000	-0.085941000
8	-0.223860000	2.259087000	0.620347000
6	-0.663940000	3.419108000	1.347435000
6	0.665785000	3.991125000	1.876656000
1	0.463184000	4.894458000	2.474374000
1	1.177879000	3.259078000	2.518738000
1	1.334182000	4.266438000	1.046664000
6	-1.337367000	4.425235000	0.404012000
1	-0.659475000	4.681566000	-0.425148000
1	-2.262583000	4.019263000	-0.027085000
1	-1.592254000	5.351028000	0.943604000
6	-1.570316000	3.008584000	2.516342000
1	-2.427029000	2.426450000	2.152065000
1	-1.020372000	2.384393000	3.235401000
1	-1.953376000	3.893955000	3.047105000
16	-3.053546000	1.332028000	-1.236944000
6	-4.297249000	0.387490000	-2.258082000
1	-3.735508000	-0.230185000	-2.970616000
1	-4.928965000	-0.253685000	-1.628766000
1	-4.916168000	1.112883000	-2.807096000
6	-4.290734000	2.084007000	-0.126035000
1	-3.764230000	2.589878000	0.687957000
1	-4.867300000	2.819150000	-0.705208000
1	-4.975099000	1.322890000	0.271819000

^{3}I1$_{CCA,C\alpha}$:

26	3.520884000	1.096577000	6.485876000
7	5.090375000	2.378422000	6.824371000
6	6.180393000	2.110496000	6.042885000
1	6.052235000	1.366431000	5.263309000
7	2.029708000	-0.122117000	6.113650000
6	7.414463000	2.731486000	6.181467000
1	8.230489000	2.441782000	5.517500000
6	5.130752000	-0.417486000	8.265188000
1	5.742003000	0.468731000	8.493347000

1	5.306094000	-1.163802000	9.055674000
6	-0.110119000	-1.868245000	5.776191000
1	-0.952499000	-2.551995000	5.648708000
7	4.740403000	-0.338390000	5.906889000
6	0.132719000	-0.851880000	4.843779000
1	-0.505675000	-0.717053000	3.968739000
6	5.449688000	-0.948910000	6.895360000
6	5.743430000	-1.769570000	4.276687000
1	5.828204000	-2.070640000	3.231288000
7	3.692913000	0.006505000	8.224714000
6	5.207036000	3.414379000	7.715896000
6	4.863657000	-0.746145000	4.625303000
1	4.249800000	-0.211764000	3.900245000
6	1.809386000	-1.108987000	7.016417000
6	6.340841000	-1.980361000	6.609741000
1	6.891255000	-2.462374000	7.420031000
6	6.499687000	-2.387649000	5.278658000
1	7.195413000	-3.192462000	5.031041000
6	6.440491000	4.060645000	7.905900000
1	6.493699000	4.866601000	8.640975000
6	2.811251000	-1.218414000	8.129658000
1	3.450261000	-2.095222000	7.943259000
1	2.318203000	-1.397584000	9.096719000
6	7.566004000	3.709531000	7.165736000
1	8.521554000	4.213269000	7.327781000
6	0.739383000	-1.995799000	6.878315000
1	0.581921000	-2.775757000	7.626373000
6	1.207835000	0.009745000	5.046026000
1	1.453590000	0.831033000	4.372004000
6	2.680100000	3.987500000	7.865925000
1	1.961197000	4.527404000	8.499489000
1	2.699513000	4.485904000	6.886097000
6	3.327201000	0.829855000	9.414910000
1	4.139607000	1.546595000	9.573629000
1	3.272812000	0.191134000	10.313314000
6	1.993212000	1.524004000	9.180835000
1	1.776559000	2.271882000	9.956200000
1	1.152581000	0.815715000	9.167568000
16	1.935910000	2.357792000	7.525977000
6	4.048528000	3.961639000	8.523241000
1	3.994216000	3.478793000	9.511951000
1	4.299532000	5.008407000	8.757254000
8	3.278301000	1.867572000	4.863136000
6	6.149031000	4.529399000	3.333402000
6	5.546470000	5.388176000	4.475506000
6	4.305229000	6.156865000	4.004183000
6	3.246327000	5.214466000	3.416265000
6	3.835551000	4.337884000	2.277593000
6	5.102861000	3.674285000	2.703571000
1	3.874173000	6.727371000	4.844657000
1	5.274837000	4.727822000	5.317899000
1	6.317463000	6.075899000	4.858824000
1	6.548454000	5.218895000	2.561203000
1	6.990743000	3.915747000	3.685057000
1	2.846690000	4.553156000	4.206938000

1	2.387240000	5.783349000	3.024976000
1	3.099277000	3.598940000	1.920737000
1	4.060610000	5.006192000	1.420398000
1	3.899839000	2.613043000	4.722330000
1	4.602731000	6.897135000	3.238296000
6	5.294484000	2.262763000	2.509862000
1	4.451030000	1.724941000	2.017361000
8	6.319778000	1.626740000	2.850540000

^{3}I1$_{CCA,ald}$:

26	0.563765000	-0.051233000	-0.673017000
7	1.204303000	0.395419000	1.232677000
6	2.485799000	-0.015887000	1.475295000
1	3.012175000	-0.469357000	0.642095000
7	-0.014816000	-0.444905000	-2.508308000
6	3.130587000	0.097918000	2.701562000
1	4.155055000	-0.265550000	2.798269000
6	-0.032265000	-2.460561000	0.712967000
1	0.120239000	-1.954875000	1.677924000
1	-0.591407000	-3.391188000	0.899034000
6	-1.034235000	-1.021047000	-5.033782000
1	-1.440336000	-1.244980000	-6.022924000
7	1.705646000	-1.657394000	-0.721718000
6	-0.115509000	0.020864000	-4.857514000
1	0.220330000	0.631977000	-5.697309000
6	1.283401000	-2.711053000	0.028787000
6	3.615920000	-2.908344000	-1.432645000
1	4.527651000	-2.952827000	-2.030752000
7	-0.796210000	-1.531785000	-0.179142000
6	0.565124000	1.052587000	2.252134000
6	2.836144000	-1.753191000	-1.456322000
1	3.096197000	-0.865351000	-2.034971000
6	-0.895175000	-1.462945000	-2.670799000
6	2.019275000	-3.892771000	0.091872000
1	1.656387000	-4.726143000	0.696810000
6	3.209941000	-3.988074000	-0.639118000
1	3.804804000	-4.903348000	-0.602088000
6	1.165832000	1.174072000	3.519039000
1	0.612651000	1.689752000	4.306492000
6	-1.197574000	-2.263529000	-1.437928000
1	-0.631073000	-3.206146000	-1.487903000
1	-2.260127000	-2.544031000	-1.386632000
6	2.442864000	0.678710000	3.768139000
1	2.898729000	0.776555000	4.755731000
6	-1.430029000	-1.771705000	-3.923273000
1	-2.142892000	-2.593390000	-4.019644000
6	0.369505000	0.288003000	-3.579795000
1	1.075835000	1.088821000	-3.357916000
6	-1.031427000	2.468341000	0.795118000
1	-1.966281000	3.044664000	0.853888000
1	-0.219992000	3.172235000	0.559557000
6	-1.993490000	-0.967591000	0.508786000
1	-1.689201000	-0.708517000	1.528129000
1	-2.780533000	-1.737088000	0.591627000
6	-2.521834000	0.240714000	-0.250856000

1	-3.283351000	0.785410000	0.324114000
1	-2.975847000	-0.038527000	-1.212419000
16	-1.167246000	1.426268000	-0.697463000
6	-0.783722000	1.720840000	2.094983000
1	-1.604372000	1.014666000	2.296748000
1	-0.864724000	2.462927000	2.904441000
8	1.776729000	1.104407000	-1.338063000
6	4.185100000	3.850208000	2.400464000
6	3.497574000	4.803815000	3.390851000
6	2.842150000	5.991087000	2.667694000
6	1.854299000	5.516413000	1.590824000
6	2.526940000	4.570930000	0.582540000
6	3.174434000	3.381156000	1.337905000
1	2.326290000	6.642158000	3.393782000
1	2.728123000	4.246970000	3.958651000
1	4.234793000	5.160316000	4.130572000
1	5.024703000	4.363088000	1.898459000
1	4.607456000	2.977591000	2.925611000
1	1.008611000	4.991518000	2.074934000
1	1.424016000	6.377257000	1.051797000
1	1.801498000	4.201052000	-0.161343000
1	3.309619000	5.112666000	0.021971000
1	2.353659000	2.831206000	1.830748000
1	3.628366000	6.608831000	2.193533000
6	3.767599000	2.431375000	0.323779000
1	2.384757000	1.537304000	-0.662267000
8	4.909548000	2.211592000	0.044027000

$^{3}PC_{CCA,C\alpha}$:

26	3.885621000	1.114257000	6.537090000
7	5.445996000	2.399035000	6.894668000
6	6.540446000	2.183442000	6.111415000
1	6.465750000	1.371972000	5.394493000
7	2.391590000	-0.081000000	5.948154000
6	7.710979000	2.928297000	6.174779000
1	8.534731000	2.684441000	5.501471000
6	5.133866000	-0.540282000	8.538560000
1	5.686485000	0.352446000	8.873929000
1	5.261352000	-1.323465000	9.306138000
6	0.299795000	-1.784780000	5.225220000
1	-0.523406000	-2.448670000	4.951162000
7	5.152505000	-0.345054000	6.119772000
6	0.682607000	-0.733360000	4.383791000
1	0.178810000	-0.547423000	3.433503000
6	5.660160000	-1.005733000	7.199760000
6	6.382011000	-1.803169000	4.654337000
1	6.629577000	-2.094383000	3.631964000
7	3.713824000	-0.154826000	8.344007000
6	5.511830000	3.440702000	7.783629000
6	5.492516000	-0.752959000	4.873353000
1	5.041885000	-0.202615000	4.045163000
6	2.040865000	-1.112010000	6.756742000
6	6.559957000	-2.060346000	7.045837000
1	6.946365000	-2.571136000	7.930512000
6	6.937724000	-2.459512000	5.758352000

1	7.640614000	-3.284205000	5.620355000
6	6.672631000	4.223444000	7.891730000
1	6.682823000	5.043476000	8.612451000
6	2.867043000	-1.327633000	8.002060000
1	3.515222000	-2.202640000	7.833155000
1	2.216322000	-1.593653000	8.851217000
6	7.788393000	3.974442000	7.095433000
1	8.687012000	4.589339000	7.184153000
6	0.988671000	-1.970771000	6.426212000
1	0.723194000	-2.780523000	7.109952000
6	1.723733000	0.099333000	4.782008000
1	2.056939000	0.940836000	4.176615000
6	2.998213000	3.968710000	8.028693000
1	2.273621000	4.401292000	8.733514000
1	3.055368000	4.637371000	7.157461000
6	3.127360000	0.649829000	9.438816000
1	3.913630000	1.301069000	9.836544000
1	2.798013000	0.002412000	10.272970000
6	1.927706000	1.460945000	8.944622000
1	1.578397000	2.166730000	9.711476000
1	1.078523000	0.811338000	8.687543000
16	2.246860000	2.428969000	7.387097000
6	4.360130000	3.802198000	8.692531000
1	4.295382000	3.092529000	9.530686000
1	4.606977000	4.765280000	9.164989000
8	3.453487000	2.725994000	4.465392000
6	5.361822000	3.857896000	3.435170000
6	5.328736000	5.066875000	4.377773000
6	4.385672000	6.157405000	3.847123000
6	2.973480000	5.604766000	3.599929000
6	3.001838000	4.369988000	2.678238000
6	3.964097000	3.287767000	3.205875000
1	4.341081000	7.004020000	4.552792000
1	4.990883000	4.740316000	5.375845000
1	6.348405000	5.464811000	4.506642000
1	5.768810000	4.153168000	2.452340000
1	6.022151000	3.065243000	3.814885000
1	2.497106000	5.353888000	4.566111000
1	2.327353000	6.371798000	3.141842000
1	1.990544000	3.942543000	2.550115000
1	3.346307000	4.669067000	1.672590000
1	2.564701000	3.096159000	4.626798000
1	4.789079000	6.557594000	2.897642000
6	3.953969000	2.099406000	2.244829000
1	2.958079000	1.594071000	2.159857000
8	4.915275000	1.688193000	1.618322000

^{3}I1$_{CCA,ald}$:

26	-0.191941000	-0.654587000	0.328886000
7	0.408856000	-0.122307000	2.150551000
6	1.556102000	0.598783000	2.236245000
1	2.037945000	0.856327000	1.293613000
7	-0.387977000	0.100106000	-1.537218000
6	2.087428000	1.028565000	3.447545000
1	3.008913000	1.613518000	3.450646000

6	-0.190450000	-3.461438000	-0.072156000
1	-0.422432000	-3.635079000	0.991368000
1	-0.371342000	-4.407547000	-0.609492000
6	-0.826584000	0.970245000	-4.143851000
1	-1.004109000	1.309477000	-5.167198000
7	1.427169000	-1.651279000	-0.114569000
6	-0.384756000	1.865574000	-3.161307000
1	-0.206955000	2.918478000	-3.388574000
6	1.239000000	-3.001203000	-0.230176000
6	3.755944000	-1.947023000	-0.634101000
1	4.729806000	-1.486380000	-0.810442000
7	-1.078188000	-2.363005000	-0.557919000
6	-0.263320000	-0.433250000	3.297307000
6	2.658314000	-1.136349000	-0.347559000
1	2.743889000	-0.048228000	-0.325996000
6	-0.812877000	-0.773032000	-2.480545000
6	2.296908000	-3.861893000	-0.518439000
1	2.109152000	-4.933631000	-0.612099000
6	3.580113000	-3.333003000	-0.703293000
1	4.422015000	-3.992206000	-0.926978000
6	0.235860000	-0.040791000	4.545147000
1	-0.320139000	-0.310815000	5.445511000
6	-0.964928000	-2.212022000	-2.044069000
1	-0.074278000	-2.766456000	-2.379748000
1	-1.829046000	-2.682961000	-2.539100000
6	1.422050000	0.692666000	4.630875000
1	1.814039000	1.001561000	5.602920000
6	-1.048978000	-0.366008000	-3.795822000
1	-1.402170000	-1.090974000	-4.533021000
6	-0.184181000	1.398217000	-1.864096000
1	0.138114000	2.049581000	-1.050546000
6	-2.656793000	-0.303291000	2.490936000
1	-3.626222000	-0.820525000	2.484014000
1	-2.787309000	0.655101000	3.012381000
6	-2.495634000	-2.506821000	-0.118609000
1	-2.494424000	-2.885635000	0.913074000
1	-3.021120000	-3.253092000	-0.740786000
6	-3.204903000	-1.163332000	-0.195576000
1	-4.227498000	-1.216353000	0.202725000
1	-3.267299000	-0.782599000	-1.225713000
16	-2.277442000	0.146179000	0.735634000
6	-1.580288000	-1.152512000	3.182625000
1	-1.454118000	-2.118610000	2.671157000
1	-1.944399000	-1.392185000	4.193635000
8	1.082058000	3.309566000	0.527437000
6	3.831414000	4.578820000	1.628921000
6	4.685890000	5.844571000	1.796232000
6	5.750811000	5.958235000	0.694874000
6	5.115009000	5.900806000	-0.702930000
6	4.263593000	4.634598000	-0.881268000
6	3.186900000	4.537215000	0.214075000
1	6.324771000	6.892863000	0.816578000
1	4.027060000	6.732987000	1.765981000
1	5.158503000	5.837342000	2.793364000
1	4.462842000	3.680059000	1.752478000

1	3.051013000	4.534029000	2.407174000
1	4.480559000	6.794875000	-0.855473000
1	5.894716000	5.935137000	-1.482834000
1	3.787347000	4.622485000	-1.876183000
1	4.906552000	3.738752000	-0.824250000
1	2.506637000	5.406242000	0.125544000
1	6.474885000	5.127745000	0.800523000
6	2.376061000	3.264202000	0.089603000
1	0.840282000	4.214098000	0.813872000
8	2.804334000	2.195773000	-0.323070000

$^3Re_{CCA}$:

26	0.575867000	-0.004181000	0.215524000
7	0.267253000	-0.350236000	2.236376000
6	1.355220000	-0.911559000	2.837916000
1	2.253939000	-0.984266000	2.234352000
7	0.845686000	0.248794000	-1.719056000
6	1.365294000	-1.368326000	4.148466000
1	2.276710000	-1.814040000	4.550632000
6	-0.200928000	-2.761624000	0.287100000
1	-0.493096000	-2.672051000	1.344705000
1	-0.651351000	-3.685390000	-0.108601000
6	1.113573000	0.571120000	-4.465050000
1	1.212391000	0.698454000	-5.545388000
7	1.849213000	-1.516230000	0.093748000
6	1.757861000	1.446104000	-3.581019000
1	2.375523000	2.270659000	-3.940919000
6	1.298790000	-2.760039000	0.150245000
6	4.024845000	-2.469232000	-0.167476000
1	5.097157000	-2.314625000	-0.299381000
7	-0.699124000	-1.553044000	-0.441495000
6	-0.829875000	-0.107137000	3.014316000
6	3.181434000	-1.362411000	-0.078236000
1	3.554400000	-0.334806000	-0.117362000
6	0.229877000	-0.613763000	-2.561909000
6	2.093548000	-3.901846000	0.066005000
1	1.629152000	-4.888966000	0.107317000
6	3.477581000	-3.754620000	-0.084728000
1	4.118699000	-4.636420000	-0.151660000
6	-0.881322000	-0.579212000	4.337660000
1	-1.786549000	-0.392000000	4.919421000
6	-0.519946000	-1.748274000	-1.924664000
1	0.043471000	-2.678907000	-2.093794000
1	-1.500517000	-1.894647000	-2.401963000
6	0.200676000	-1.241012000	4.910029000
1	0.149961000	-1.609341000	5.937208000
6	0.340157000	-0.470290000	-3.947440000
1	-0.173576000	-1.175348000	-4.604437000
6	1.599074000	1.260539000	-2.212028000
1	2.065157000	1.901251000	-1.463523000
6	-1.698748000	1.886807000	1.623606000
1	-2.585144000	2.526375000	1.503613000
1	-0.872746000	2.512525000	1.992966000
6	-2.116566000	-1.228448000	-0.112187000
1	-2.231750000	-1.341843000	0.970769000

1	-2.796694000	-1.954612000	-0.589893000
6	-2.458436000	0.182279000	-0.575772000
1	-3.433228000	0.514210000	-0.191639000
1	-2.499598000	0.262172000	-1.671489000
16	-1.178705000	1.424578000	-0.064265000
6	-2.005783000	0.728411000	2.556669000
1	-2.815407000	0.101964000	2.149314000
1	-2.446271000	1.169596000	3.464911000
8	1.657014000	1.183433000	0.603149000
6	3.965372000	4.394821000	-0.671799000
6	3.654742000	5.899653000	-0.640439000
6	2.299538000	6.181908000	0.027026000
6	2.239839000	5.581619000	1.440866000
6	2.530854000	4.073565000	1.419352000
6	3.888300000	3.782385000	0.732602000
1	2.109728000	7.268733000	0.064519000
1	4.452842000	6.424538000	-0.080989000
1	3.671864000	6.307940000	-1.665746000
1	3.233720000	3.883832000	-1.325188000
1	4.957765000	4.208429000	-1.114297000
1	2.981839000	6.089807000	2.086388000
1	1.250263000	5.762078000	1.895681000
1	2.529973000	3.658834000	2.442374000
1	1.734523000	3.544826000	0.867467000
1	4.672171000	4.245183000	1.375111000
1	1.491075000	5.742353000	-0.588828000
6	4.205079000	2.308950000	0.768721000
1	4.074463000	1.823037000	1.768104000
8	4.602643000	1.645340000	-0.183113000

^{3}TS1$_{CCA,C\alpha}$:

26	3.639196000	1.199106000	6.374827000
7	5.264409000	2.375222000	6.806349000
6	6.391753000	2.014904000	6.124949000
1	6.268537000	1.291887000	5.325826000
7	2.091168000	0.020111000	6.020257000
6	7.655093000	2.526937000	6.383840000
1	8.501596000	2.173013000	5.793308000
6	5.167173000	-0.408511000	8.178933000
1	5.822697000	0.443340000	8.413954000
1	5.305560000	-1.166721000	8.965074000
6	-0.119138000	-1.633073000	5.688662000
1	-0.989683000	-2.280915000	5.563492000
7	4.799175000	-0.304674000	5.810013000
6	0.171583000	-0.637457000	4.747273000
1	-0.455840000	-0.482579000	3.867761000
6	5.464609000	-0.950297000	6.807191000
6	5.745635000	-1.788261000	4.191085000
1	5.823632000	-2.095861000	3.147104000
7	3.753278000	0.084633000	8.140882000
6	5.377783000	3.400638000	7.708147000
6	4.912974000	-0.724086000	4.531482000
1	4.325716000	-0.172127000	3.797626000
6	1.826038000	-0.945742000	6.932491000
6	6.307792000	-2.024161000	6.530086000

1	6.824884000	-2.531836000	7.346519000
6	6.462211000	-2.439459000	5.201487000
1	7.121613000	-3.276533000	4.961877000
6	6.638369000	3.940617000	8.016195000
1	6.690372000	4.740862000	8.757475000
6	2.814207000	-1.092384000	8.053084000
1	3.407787000	-2.003473000	7.880698000
1	2.306137000	-1.236884000	9.018389000
6	7.794066000	3.490032000	7.385302000
1	8.769693000	3.909073000	7.641502000
6	0.718465000	-1.785952000	6.796000000
1	0.525221000	-2.550568000	7.551260000
6	1.281244000	0.178223000	4.946870000
1	1.565442000	0.983810000	4.268374000
6	2.863738000	4.097737000	7.698949000
1	2.146241000	4.696854000	8.278430000
1	2.934362000	4.539187000	6.694336000
6	3.427375000	0.946688000	9.314520000
1	4.265449000	1.639289000	9.449011000
1	3.358171000	0.332654000	10.229200000
6	2.116083000	1.682191000	9.075550000
1	1.931853000	2.452732000	9.836829000
1	1.250127000	1.005192000	9.081596000
16	2.069845000	2.486731000	7.405791000
6	4.202772000	4.052800000	8.410426000
1	4.082506000	3.641059000	9.425945000
1	4.493757000	5.100940000	8.587358000
8	3.414478000	1.954912000	4.842232000
6	5.801781000	4.428799000	3.816934000
6	5.056789000	5.440766000	4.702503000
6	3.841858000	6.042009000	3.979646000
6	2.897066000	4.951504000	3.449155000
6	3.640010000	3.956463000	2.537443000
6	4.875317000	3.388900000	3.222086000
1	3.295943000	6.723337000	4.655014000
1	4.726736000	4.931376000	5.624174000
1	5.749510000	6.235834000	5.024830000
1	6.266731000	4.976040000	2.969577000
1	6.633690000	3.953806000	4.355921000
1	2.455769000	4.392414000	4.294617000
1	2.055900000	5.405128000	2.898775000
1	2.969627000	3.142185000	2.212066000
1	3.960758000	4.484431000	1.615672000
1	4.224755000	2.641986000	4.240175000
1	4.195641000	6.659303000	3.132072000
6	5.476459000	2.193792000	2.627626000
1	4.785715000	1.590184000	1.985685000
8	6.632552000	1.792211000	2.825031000

^{3}TS1$_{CCA,ald}$:

26	0.589161000	-0.020212000	-0.595035000
7	1.160882000	0.366067000	1.348778000
6	2.422016000	-0.082075000	1.625497000
1	2.946727000	-0.576150000	0.815096000
7	0.054027000	-0.380330000	-2.458512000

6	3.055137000	0.057471000	2.854613000
1	4.067530000	-0.331184000	2.977013000
6	-0.080937000	-2.474039000	0.694884000
1	0.066645000	-2.007053000	1.680262000
1	-0.656592000	-3.401697000	0.840824000
6	-0.879350000	-0.909477000	-5.027366000
1	-1.251851000	-1.115119000	-6.033552000
7	1.709093000	-1.645917000	-0.664964000
6	0.057791000	0.107444000	-4.805822000
1	0.442249000	0.716556000	-5.625929000
6	1.241640000	-2.723098000	0.022514000
6	3.604127000	-2.911792000	-1.391929000
1	4.529195000	-2.951218000	-1.969561000
7	-0.818333000	-1.502716000	-0.172257000
6	0.521710000	1.063862000	2.340406000
6	2.856007000	-1.735902000	-1.374549000
1	3.153857000	-0.828586000	-1.902445000
6	-0.843674000	-1.374600000	-2.664284000
6	1.944424000	-3.926427000	0.042344000
1	1.544695000	-4.778544000	0.595909000
6	3.149003000	-4.018794000	-0.665496000
1	3.717131000	-4.951648000	-0.662424000
6	1.109243000	1.209586000	3.610898000
1	0.558056000	1.760104000	4.375676000
6	-1.224530000	-2.180402000	-1.456466000
1	-0.718837000	-3.156833000	-1.514023000
1	-2.303885000	-2.395102000	-1.442513000
6	2.372480000	0.696238000	3.890684000
1	2.820844000	0.820014000	4.878803000
6	-1.335993000	-1.658744000	-3.940340000
1	-2.064848000	-2.461519000	-4.070938000
6	0.498911000	0.350756000	-3.508118000
1	1.218093000	1.129869000	-3.251633000
6	-1.013689000	2.506714000	0.846144000
1	-1.938479000	3.100719000	0.883796000
1	-0.183038000	3.195438000	0.634060000
6	-2.003986000	-0.925896000	0.522014000
1	-1.694600000	-0.682403000	1.543958000
1	-2.807168000	-1.679591000	0.596770000
6	-2.508243000	0.299772000	-0.226805000
1	-3.266310000	0.849354000	0.348086000
1	-2.959234000	0.037429000	-1.194476000
16	-1.132785000	1.471258000	-0.651591000
6	-0.813156000	1.749921000	2.148917000
1	-1.646695000	1.051581000	2.321559000
1	-0.909915000	2.488648000	2.959391000
8	1.797390000	1.107042000	-1.180702000
6	4.159673000	3.747328000	2.269525000
6	3.522470000	4.747831000	3.247452000
6	2.891341000	5.937256000	2.506023000
6	1.870553000	5.468591000	1.457303000
6	2.493768000	4.471050000	0.467203000
6	3.111286000	3.280606000	1.247315000
1	2.408245000	6.621564000	3.224136000
1	2.746058000	4.229632000	3.841926000

1	4.283118000	5.099493000	3.965352000
1	5.002810000	4.220834000	1.735856000
1	4.569014000	2.877779000	2.810164000
1	1.015295000	4.988360000	1.969963000
1	1.461972000	6.328945000	0.900841000
1	1.744286000	4.109036000	-0.257084000
1	3.287646000	4.967975000	-0.118584000
1	2.278212000	2.785491000	1.777550000
1	3.687985000	6.518575000	2.004144000
6	3.628649000	2.267717000	0.249976000
1	2.593323000	1.615903000	-0.469600000
8	4.773115000	2.005608000	-0.018180000

^{5}I1$_{CCA,C\alpha}$:

26	0.815074000	-0.217679000	-0.517469000
7	1.323207000	-0.087551000	1.664501000
6	2.674766000	-0.101662000	1.795560000
1	3.245311000	-0.323967000	0.895756000
7	0.053963000	-0.240692000	-2.529860000
6	3.333510000	0.168912000	2.990293000
1	4.423935000	0.136596000	3.023684000
6	-0.026656000	-3.001163000	0.322784000
1	-0.087524000	-2.668859000	1.371920000
1	-0.522535000	-3.985236000	0.259558000
6	-1.123592000	-0.275497000	-5.050400000
1	-1.594628000	-0.287870000	-6.036255000
7	1.967936000	-1.976573000	-0.607193000
6	-0.347781000	0.817945000	-4.645256000
1	-0.190419000	1.678238000	-5.298373000
6	1.422612000	-3.107305000	-0.092295000
6	4.043292000	-3.117127000	-0.977421000
1	5.068931000	-3.087295000	-1.348841000
7	-0.697660000	-1.983531000	-0.527733000
6	0.573199000	0.222989000	2.756722000
6	3.244131000	-1.979158000	-1.055441000
1	3.604079000	-1.036904000	-1.475152000
6	-0.687571000	-1.305401000	-2.918233000
6	2.170037000	-4.280430000	0.018234000
1	1.710826000	-5.181471000	0.430233000
6	3.499452000	-4.281513000	-0.419888000
1	4.100834000	-5.190280000	-0.341949000
6	1.179286000	0.517396000	3.988316000
1	0.547818000	0.770970000	4.842190000
6	-0.755156000	-2.450702000	-1.944250000
1	0.112421000	-3.103155000	-2.131677000
1	-1.654972000	-3.063974000	-2.113316000
6	2.567997000	0.488890000	4.115393000
1	3.043175000	0.719541000	5.071850000
6	-1.295150000	-1.352868000	-4.175144000
1	-1.895419000	-2.220741000	-4.456739000
6	0.225838000	0.801244000	-3.375529000
1	0.834766000	1.627298000	-3.004178000
6	-1.504736000	1.326209000	1.720734000
1	-2.588929000	1.424324000	1.875339000
1	-1.052214000	2.307345000	1.924019000

6	-2.056316000	-1.635339000	-0.036096000
1	-2.058375000	-1.711256000	1.056386000
1	-2.794696000	-2.372990000	-0.398319000
6	-2.497574000	-0.241366000	-0.477177000
1	-3.451645000	0.029691000	-0.003384000
1	-2.642739000	-0.174142000	-1.564426000
16	-1.283251000	1.088233000	-0.088977000
6	-0.928171000	0.253971000	2.653263000
1	-1.305659000	-0.742143000	2.387434000
1	-1.334909000	0.459397000	3.655484000
8	2.045836000	1.079508000	-0.917479000
6	3.353335000	4.321473000	-0.403940000
6	4.051000000	3.472253000	0.693065000
6	3.497131000	3.783263000	2.089255000
6	1.975106000	3.606936000	2.137200000
6	1.276554000	4.492737000	1.074190000
6	1.867263000	4.279235000	-0.277850000
1	3.973514000	3.125546000	2.835771000
1	3.902265000	2.401297000	0.465105000
1	5.136752000	3.655139000	0.649381000
1	3.671104000	5.377119000	-0.272578000
1	3.665134000	4.021703000	-1.416258000
1	1.720545000	2.552273000	1.940741000
1	1.575841000	3.850227000	3.134594000
1	0.185920000	4.332425000	1.059401000
1	1.433954000	5.553821000	1.360106000
1	1.959150000	2.036228000	-0.727163000
1	3.757386000	4.820674000	2.371035000
6	1.009157000	4.091342000	-1.421820000
1	-0.085514000	4.095707000	-1.209871000
8	1.413357000	3.927271000	-2.595641000

^{5}I1$_{CCA,ald}$:

26	0.146853000	-0.325973000	-0.650777000
7	-0.003397000	0.573110000	1.404544000
6	1.249682000	0.688000000	1.916961000
1	2.034035000	0.149330000	1.386938000
7	-0.016636000	-1.181189000	-2.611107000
6	1.557403000	1.423291000	3.059601000
1	2.584233000	1.445853000	3.429629000
6	-0.387313000	-2.839699000	0.939233000
1	-0.667178000	-2.215295000	1.803056000
1	-0.727144000	-3.869142000	1.147496000
6	-0.562863000	-2.224790000	-5.129208000
1	-0.786803000	-2.634191000	-6.117377000
7	1.573392000	-1.761421000	0.012803000
6	0.047392000	-0.969645000	-5.000641000
1	0.316998000	-0.375840000	-5.876119000
6	1.111641000	-2.796704000	0.755420000
6	3.822438000	-2.549058000	0.278049000
1	4.884915000	-2.424441000	0.061438000
7	-1.040672000	-2.269061000	-0.266374000
6	-1.013284000	1.236309000	2.032961000
6	2.897171000	-1.640864000	-0.232906000
1	3.186011000	-0.783610000	-0.845134000

6	-0.593044000	-2.399201000	-2.726654000
6	1.982999000	-3.742449000	1.299612000
1	1.587612000	-4.572367000	1.889461000
6	3.356470000	-3.610942000	1.064300000
1	4.056233000	-4.339646000	1.480558000
6	-0.761518000	2.017853000	3.174205000
1	-1.592027000	2.546460000	3.646778000
6	-0.824790000	-3.159007000	-1.444312000
1	0.074282000	-3.768442000	-1.256162000
1	-1.662000000	-3.868091000	-1.554859000
6	0.525402000	2.111329000	3.703026000
1	0.718159000	2.711243000	4.595610000
6	-0.888232000	-2.948919000	-3.978363000
1	-1.366103000	-3.929131000	-4.043862000
6	0.299190000	-0.477232000	-3.721993000
1	0.756385000	0.497096000	-3.538288000
6	-2.711687000	1.597174000	0.111010000
1	-3.794149000	1.715053000	-0.042407000
1	-2.238433000	2.570411000	-0.086646000
6	-2.482880000	-2.009818000	-0.038326000
1	-2.592795000	-1.623552000	0.981462000
1	-3.057112000	-2.954246000	-0.081481000
6	-3.072111000	-1.032643000	-1.055427000
1	-4.104227000	-0.769129000	-0.783356000
1	-3.105896000	-1.466143000	-2.065490000
16	-2.119219000	0.532697000	-1.261897000
6	-2.436022000	1.125885000	1.541656000
1	-2.804242000	0.100300000	1.687831000
1	-3.062661000	1.747049000	2.200407000
8	1.249896000	1.010657000	-1.240195000
6	2.950966000	4.678325000	1.666367000
6	4.384919000	5.224170000	1.765462000
6	5.429315000	4.098813000	1.735654000
6	5.277928000	3.235615000	0.473407000
6	3.859715000	2.657594000	0.355046000
6	2.817305000	3.791659000	0.402007000
1	6.445597000	4.524358000	1.786749000
1	4.567838000	5.923092000	0.928220000
1	4.474268000	5.814301000	2.693595000
1	2.718529000	4.065581000	2.554139000
1	2.215219000	5.496555000	1.630226000
1	5.498105000	3.847314000	-0.421664000
1	6.007242000	2.408827000	0.477622000
1	3.743377000	2.077898000	-0.573611000
1	3.675553000	1.966156000	1.193999000
1	2.949351000	4.422610000	-0.504417000
1	5.308008000	3.461461000	2.632056000
6	1.404126000	3.317311000	0.240746000
1	1.354254000	1.893954000	-0.741637000
8	0.328125000	3.704821000	0.534292000

$^{5}PC_{CCA,C\alpha}$:

26	3.923434000	1.209053000	6.286795000
7	5.656728000	2.439882000	7.117348000
6	6.737261000	2.344165000	6.303157000

1	6.771395000	1.465127000	5.660867000
7	2.141613000	0.130177000	5.713318000
6	7.765528000	3.283057000	6.251281000
1	8.601397000	3.132412000	5.565219000
6	5.057417000	-0.592549000	8.362105000
1	5.559667000	0.289040000	8.792344000
1	5.084954000	-1.399621000	9.114394000
6	-0.280720000	-1.235540000	5.392683000
1	-1.225897000	-1.770385000	5.272293000
7	5.365271000	-0.378852000	5.965868000
6	0.020193000	-0.125364000	4.595971000
1	-0.675351000	0.239308000	3.837629000
6	5.769179000	-1.010011000	7.098032000
6	6.930320000	-1.697554000	4.694437000
1	7.355227000	-1.945015000	3.719807000
7	3.665412000	-0.191718000	8.027388000
6	5.605667000	3.503993000	7.972322000
6	5.930387000	-0.730295000	4.788437000
1	5.566672000	-0.209610000	3.900522000
6	1.844672000	-0.938533000	6.494649000
6	6.765534000	-1.988633000	7.082061000
1	7.056535000	-2.481051000	8.012782000
6	7.364549000	-2.330894000	5.864949000
1	8.147422000	-3.092195000	5.829158000
6	6.593433000	4.500971000	7.950342000
1	6.508785000	5.346760000	8.636421000
6	2.898208000	-1.353898000	7.489272000
1	3.604296000	-2.024406000	6.975332000
1	2.451966000	-1.935616000	8.312949000
6	7.680794000	4.403803000	7.079293000
1	8.451118000	5.178758000	7.062274000
6	0.643014000	-1.639904000	6.362264000
1	0.436408000	-2.487755000	7.019661000
6	1.233743000	0.531367000	4.795176000
1	1.498164000	1.421117000	4.223289000
6	3.106107000	3.914450000	8.512433000
1	2.444812000	4.151851000	9.358579000
1	3.116614000	4.789149000	7.844462000
6	2.977736000	0.423499000	9.191388000
1	3.725557000	0.987243000	9.760690000
1	2.594408000	-0.362990000	9.866971000
6	1.821359000	1.337472000	8.784743000
1	1.415494000	1.856600000	9.664771000
1	0.993470000	0.777871000	8.327366000
16	2.270913000	2.609128000	7.531853000
6	4.518150000	3.593211000	9.014437000
1	4.510712000	2.674460000	9.617623000
1	4.792464000	4.397937000	9.714748000
8	3.552753000	2.647372000	4.702602000
6	5.418823000	3.663736000	3.442709000
6	5.329676000	5.003368000	4.184833000
6	4.291689000	5.934761000	3.537543000
6	2.917895000	5.257233000	3.419086000
6	3.011622000	3.909461000	2.679782000
6	4.049728000	2.981336000	3.355282000

1	4.203135000	6.869379000	4.115401000
1	5.070867000	4.826106000	5.245079000
1	6.322385000	5.481167000	4.193151000
1	5.773684000	3.817481000	2.409758000
1	6.135623000	2.984444000	3.925255000
1	2.474552000	5.126349000	4.428377000
1	2.203325000	5.902117000	2.882470000
1	2.029114000	3.410562000	2.631053000
1	3.344205000	4.075668000	1.641731000
1	3.043184000	3.408129000	5.043112000
1	4.638710000	6.218229000	2.526431000
6	4.087457000	1.661391000	2.598859000
1	3.386445000	0.877232000	2.978930000
8	4.792427000	1.459962000	1.626597000

$^{5}PC_{CCA,ald}$:

26	0.062691000	-0.399295000	0.221146000
7	-0.068394000	0.322074000	2.200247000
6	1.055387000	0.893276000	2.697325000
1	1.942594000	0.841253000	2.064889000
7	0.357543000	0.077793000	-1.794927000
6	1.114816000	1.514466000	3.940171000
1	2.051555000	1.955068000	4.285514000
6	-0.262388000	-3.364143000	0.143850000
1	-0.862961000	-3.377281000	1.067359000
1	-0.408898000	-4.337232000	-0.356334000
6	0.438407000	0.526438000	-4.550487000
1	0.468372000	0.697707000	-5.629118000
7	1.601065000	-1.855164000	0.527256000
6	0.642945000	1.581467000	-3.653100000
1	0.832493000	2.598302000	-4.002215000
6	1.193765000	-3.148716000	0.488380000
6	3.828131000	-2.581988000	1.074091000
1	4.864429000	-2.314170000	1.288208000
7	-0.743255000	-2.235658000	-0.699225000
6	-1.197692000	0.325556000	2.963744000
6	2.896197000	-1.582650000	0.800541000
1	3.186734000	-0.530575000	0.779925000
6	0.137583000	-0.943229000	-2.661225000
6	2.072393000	-4.204220000	0.743049000
1	1.714567000	-5.234893000	0.688859000
6	3.406262000	-3.917205000	1.051329000
1	4.111827000	-4.726619000	1.253266000
6	-1.206319000	0.949439000	4.219675000
1	-2.126179000	0.945652000	4.808601000
6	-0.105154000	-2.302838000	-2.052498000
1	0.869694000	-2.802993000	-1.937316000
1	-0.708603000	-2.932488000	-2.726685000
6	-0.048811000	1.554321000	4.714910000
1	-0.052518000	2.038057000	5.694679000
6	0.169953000	-0.750042000	-4.044925000
1	-0.023675000	-1.591778000	-4.713886000
6	0.584660000	1.318426000	-2.286216000
1	0.686663000	2.109259000	-1.542556000
6	-3.177595000	0.371794000	1.329024000

1	-4.120683000	-0.138705000	1.086233000
1	-3.425969000	1.396826000	1.639917000
6	-2.231152000	-2.211392000	-0.772845000
1	-2.618846000	-2.438127000	0.230205000
1	-2.588356000	-3.012803000	-1.445883000
6	-2.778694000	-0.871379000	-1.250468000
1	-3.877706000	-0.891779000	-1.254870000
1	-2.456053000	-0.629028000	-2.273299000
16	-2.231157000	0.568547000	-0.239919000
6	-2.438934000	-0.367275000	2.455636000
1	-2.195970000	-1.396722000	2.152132000
1	-3.144601000	-0.463006000	3.296197000
8	0.142356000	3.166386000	0.579638000
6	2.729971000	3.967792000	-0.942922000
6	3.904032000	3.401288000	-1.753158000
6	5.186792000	3.344683000	-0.909938000
6	4.968419000	2.551418000	0.388623000
6	3.782210000	3.101516000	1.196676000
6	2.504668000	3.124252000	0.342173000
1	6.009808000	2.899775000	-1.494986000
1	3.645126000	2.384140000	-2.104587000
1	4.060616000	4.015845000	-2.655952000
1	2.946588000	5.009978000	-0.645208000
1	1.811240000	4.000455000	-1.550869000
1	4.783125000	1.488922000	0.139586000
1	5.880224000	2.569475000	1.009352000
1	3.627327000	2.498669000	2.107585000
1	4.000213000	4.128925000	1.536954000
1	2.256102000	2.094080000	0.024104000
1	5.500200000	4.375599000	-0.657820000
6	1.306676000	3.668414000	1.088766000
1	-0.607514000	3.576322000	1.063244000
8	1.325747000	4.477374000	2.003330000

$^{5}Re_{CCA}$:

26	-0.044136000	-0.239784000	0.410894000
7	-0.449989000	-0.798460000	2.595650000
6	0.732909000	-0.823696000	3.261718000
1	1.622696000	-0.612412000	2.672764000
7	-0.048093000	0.239072000	-1.641503000
6	0.847217000	-1.086052000	4.622578000
1	1.834629000	-1.099254000	5.087597000
6	0.253442000	-3.143841000	0.336987000
1	-0.148214000	-3.231110000	1.358295000
1	0.198118000	-4.137939000	-0.134337000
6	-0.338763000	0.708149000	-4.358517000
1	-0.458850000	0.891528000	-5.428854000
7	1.765623000	-1.267289000	0.420187000
6	-0.135670000	1.774198000	-3.472858000
1	-0.088628000	2.806431000	-3.824116000
6	1.665959000	-2.623239000	0.383721000
6	4.152705000	-1.419132000	0.470845000
1	5.117279000	-0.909851000	0.500806000
7	-0.582794000	-2.146508000	-0.405934000
6	-1.596258000	-0.978591000	3.309165000

6	2.977682000	-0.671966000	0.456798000
1	2.970053000	0.418133000	0.487539000
6	-0.239876000	-0.801965000	-2.488298000
6	2.803926000	-3.428163000	0.399309000
1	2.703726000	-4.514642000	0.363312000
6	4.062967000	-2.816579000	0.449150000
1	4.967651000	-3.428657000	0.460694000
6	-1.543195000	-1.243919000	4.688795000
1	-2.479014000	-1.386231000	5.233532000
6	-0.193512000	-2.166399000	-1.863562000
1	0.842046000	-2.536301000	-1.931810000
1	-0.823725000	-2.886724000	-2.406857000
6	-0.320640000	-1.316314000	5.354494000
1	-0.283310000	-1.527741000	6.425874000
6	-0.396519000	-0.598125000	-3.860629000
1	-0.561196000	-1.450072000	-4.523524000
6	-0.004052000	1.505536000	-2.112410000
1	0.134491000	2.300194000	-1.375669000
6	-3.225467000	0.316451000	1.769410000
1	-4.302817000	0.403242000	1.567068000
1	-2.896046000	1.254523000	2.240889000
6	-2.039468000	-2.430793000	-0.229153000
1	-2.187546000	-2.678556000	0.826916000
1	-2.315724000	-3.325584000	-0.813880000
6	-2.912900000	-1.255792000	-0.649830000
1	-3.967899000	-1.444555000	-0.405055000
1	-2.862558000	-1.067440000	-1.731614000
16	-2.418869000	0.350484000	0.122086000
6	-2.960477000	-0.890403000	2.665587000
1	-3.213581000	-1.826022000	2.144374000
1	-3.698128000	-0.831909000	3.481154000
8	0.520726000	1.228257000	0.890710000
6	2.920199000	3.018117000	-0.850125000
6	4.363847000	2.501014000	-0.756892000
6	5.368638000	3.539289000	-1.280748000
6	5.211036000	4.884081000	-0.553220000
6	3.772287000	5.415867000	-0.653051000
6	2.763101000	4.359751000	-0.126431000
1	6.400563000	3.164090000	-1.169366000
1	4.604087000	2.267580000	0.298803000
1	4.462239000	1.556247000	-1.319333000
1	2.639607000	3.145925000	-1.911703000
1	2.210738000	2.289652000	-0.425202000
1	5.476297000	4.758573000	0.513844000
1	5.910900000	5.630405000	-0.967054000
1	3.667096000	6.359685000	-0.091019000
1	3.533297000	5.641361000	-1.709213000
1	2.978625000	4.227708000	0.956797000
1	5.203082000	3.689761000	-2.364815000
6	1.372179000	4.931762000	-0.186439000
1	1.238575000	5.897838000	0.365146000
8	0.421768000	4.444741000	-0.787525000

^{5}TS1$_{CCA,ald}$:

26	0.109946000	-0.317922000	-0.617835000

7	-0.093016000	0.536932000	1.473850000
6	1.142720000	0.616705000	2.031519000
1	1.923299000	0.029738000	1.550554000
7	0.012866000	-1.119472000	-2.565240000
6	1.442217000	1.385717000	3.153010000
1	2.458153000	1.387785000	3.552531000
6	-0.274059000	-2.798894000	0.957089000
1	-0.538608000	-2.190227000	1.836540000
1	-0.597464000	-3.834656000	1.154932000
6	-0.470263000	-2.165979000	-5.092342000
1	-0.668705000	-2.576815000	-6.085199000
7	1.625178000	-1.667677000	-0.019556000
6	0.119620000	-0.902466000	-4.950511000
1	0.397537000	-0.303562000	-5.819722000
6	1.215219000	-2.727379000	0.721449000
6	3.899323000	-2.400594000	0.153776000
1	4.950164000	-2.244603000	-0.096348000
7	-0.973520000	-2.219210000	-0.224065000
6	-1.097937000	1.257765000	2.045726000
6	2.935180000	-1.507537000	-0.309382000
1	3.182330000	-0.633932000	-0.915513000
6	-0.545628000	-2.346589000	-2.691738000
6	2.127669000	-3.659513000	1.217802000
1	1.774870000	-4.508744000	1.806793000
6	3.488311000	-3.487872000	0.936000000
1	4.220699000	-4.205109000	1.314017000
6	-0.854126000	2.069426000	3.167181000
1	-1.679687000	2.642890000	3.593910000
6	-0.791178000	-3.109069000	-1.415708000
1	0.088532000	-3.748473000	-1.236216000
1	-1.651299000	-3.789532000	-1.519692000
6	0.418683000	2.139818000	3.732103000
1	0.606535000	2.770447000	4.604367000
6	-0.809266000	-2.896714000	-3.949826000
1	-1.272354000	-3.882990000	-4.026567000
6	0.339228000	-0.406922000	-3.667955000
1	0.772877000	0.575758000	-3.474980000
6	-2.727604000	1.635370000	0.069489000
1	-3.799500000	1.796245000	-0.115946000
1	-2.203835000	2.580481000	-0.138604000
6	-2.412424000	-1.980927000	0.065017000
1	-2.472227000	-1.554357000	1.072787000
1	-2.961242000	-2.940483000	0.089067000
6	-3.068033000	-1.058676000	-0.959989000
1	-4.092855000	-0.805073000	-0.653453000
1	-3.135625000	-1.529773000	-1.951399000
16	-2.148128000	0.513773000	-1.260884000
6	-2.509426000	1.184493000	1.515057000
1	-2.922998000	0.177416000	1.671236000
1	-3.131296000	1.842810000	2.141355000
8	1.071512000	1.057419000	-1.115061000
6	2.936342000	4.621562000	1.539343000
6	4.395620000	5.083369000	1.682956000
6	5.366074000	3.893668000	1.737180000
6	5.212949000	2.999015000	0.497148000

6	3.766451000	2.508053000	0.336497000
6	2.789029000	3.699010000	0.310320000
1	6.405453000	4.253625000	1.822136000
1	4.658373000	5.734152000	0.827990000
1	4.489153000	5.703612000	2.590954000
1	2.628704000	4.062385000	2.440819000
1	2.255824000	5.482507000	1.445783000
1	5.508489000	3.565864000	-0.405825000
1	5.888667000	2.129577000	0.558943000
1	3.650854000	1.909408000	-0.580724000
1	3.509508000	1.851755000	1.184901000
1	2.989024000	4.282883000	-0.615148000
1	5.162364000	3.296766000	2.646515000
6	1.353764000	3.261718000	0.117343000
1	1.237334000	2.099330000	-0.587513000
8	0.329749000	3.760126000	0.479889000

^{5}TS1$_{CCA,C\alpha}$:

26	0.611616000	-0.120115000	-0.410037000
7	0.957086000	-0.041944000	1.802014000
6	2.284183000	-0.232436000	2.028355000
1	2.873056000	-0.572396000	1.179514000
7	-0.062734000	-0.229127000	-2.427212000
6	2.902793000	-0.009742000	3.253252000
1	3.974881000	-0.186954000	3.354573000
6	0.356609000	-2.959335000	0.320489000
1	0.396682000	-2.678978000	1.384804000
1	-0.031144000	-3.989074000	0.258313000
6	-1.294693000	-0.342133000	-4.912930000
1	-1.788881000	-0.384323000	-5.886423000
7	2.060257000	-1.599651000	-0.719494000
6	-0.515207000	0.767293000	-4.558780000
1	-0.376081000	1.607573000	-5.241289000
6	1.730599000	-2.846415000	-0.292568000
6	4.193543000	-2.388417000	-1.462397000
1	5.154473000	-2.173759000	-1.933022000
7	-0.545020000	-1.993070000	-0.374025000
6	0.190465000	0.415821000	2.834387000
6	3.258471000	-1.368222000	-1.299944000
1	3.439959000	-0.338154000	-1.612850000
6	-0.793476000	-1.315813000	-2.769232000
6	2.622380000	-3.910395000	-0.424159000
1	2.337445000	-4.907096000	-0.081369000
6	3.872004000	-3.675013000	-1.011173000
1	4.585421000	-4.494532000	-1.124964000
6	0.762851000	0.664069000	4.093630000
1	0.123644000	1.034703000	4.897448000
6	-0.788404000	-2.450643000	-1.781999000
1	0.028175000	-3.133963000	-2.066122000
1	-1.719189000	-3.037053000	-1.833321000
6	2.122423000	0.450985000	4.316163000
1	2.563428000	0.650742000	5.295552000
6	-1.435446000	-1.398556000	-4.007887000
1	-2.031632000	-2.279443000	-4.255345000
6	0.080429000	0.792348000	-3.300691000

1	0.687564000	1.629133000	-2.953171000
6	-1.726673000	1.602150000	1.553345000
1	-2.780291000	1.885371000	1.691426000
1	-1.133134000	2.528114000	1.544781000
6	-1.824488000	-1.808722000	0.363457000
1	-1.574008000	-1.729403000	1.426804000
1	-2.459495000	-2.705300000	0.247551000
6	-2.598011000	-0.585472000	-0.119232000
1	-3.477671000	-0.407534000	0.515784000
1	-2.964124000	-0.709517000	-1.148401000
16	-1.601604000	0.971844000	-0.163556000
6	-1.290913000	0.665925000	2.679482000
1	-1.842392000	-0.284741000	2.626619000
1	-1.639595000	1.130895000	3.614591000
8	1.623561000	1.242331000	-0.721123000
6	3.707968000	3.628770000	-0.412362000
6	4.153205000	2.900362000	0.870446000
6	3.490667000	3.494865000	2.122261000
6	1.960026000	3.485479000	2.000948000
6	1.490518000	4.228843000	0.738240000
6	2.203961000	3.740412000	-0.503942000
1	3.799598000	2.930287000	3.018457000
1	3.881607000	1.832593000	0.783952000
1	5.251693000	2.941343000	0.955938000
1	4.123499000	4.658360000	-0.408419000
1	4.108701000	3.141863000	-1.315904000
1	1.610667000	2.441870000	1.942552000
1	1.490792000	3.929374000	2.893872000
1	0.396560000	4.157752000	0.605970000
1	1.712006000	5.310641000	0.857252000
1	1.808292000	2.388018000	-0.591917000
1	3.842456000	4.533738000	2.268875000
6	1.590545000	4.070344000	-1.798536000
1	0.499004000	4.311267000	-1.752181000
8	2.171449000	4.051494000	-2.889196000
